# The MicroRNA Expression Signature of Bladder Cancer by Deep Sequencing: The Functional Significance of the *miR-195/497* Cluster

**DOI:** 10.1371/journal.pone.0084311

**Published:** 2014-02-10

**Authors:** Toshihiko Itesako, Naohiko Seki, Hirofumi Yoshino, Takeshi Chiyomaru, Takeshi Yamasaki, Hideo Hidaka, Tomokazu Yonezawa, Nijiro Nohata, Takashi Kinoshita, Masayuki Nakagawa, Hideki Enokida

**Affiliations:** 1 Department of Urology, Graduate School of Medical and Dental Sciences, Kagoshima University, Kagoshima, Japan; 2 Department of Functional Genomics, Chiba University Graduate School of Medicine, Chiba, Japan; The University of Arizona, United States of America

## Abstract

Current genome-wide microRNA (miRNA) expression signature analysis using deep sequencing technologies can drive the discovery of novel cancer pathways regulated by oncogenic and/or tumor suppressive miRNAs. We determined the genome-wide miRNA expression signature in bladder cancer (BC) by deep sequencing technology. A total of ten small RNA libraries were sequenced (five BCs and five samples of histologically normal bladder epithelia (NBE)), and 13,190,619 to 18,559,060 clean small RNA reads were obtained. A total of 933 known miRNAs and 17 new miRNA candidates were detected in this analysis. Among the known miRNAs, a total of 60 miRNAs were significantly downregulated in BC compared with NBE. We also found that several miRNAs, such as *miR-1/133a*, *miR-206/133b*, *let-7c/miR-99a*, *miR-143/145* and *miR-195/497*, were located close together at five distinct loci and constituted clustered miRNAs. Among these clustered miRNAs, we focused on the *miR-195/497* cluster because this clustered miRNA had not been analyzed in BC. Transfection of mature *miR-195* or *miR-497* in two BC cell lines (BOY and T24) significantly inhibited cancer cell proliferation, migration and invasion, suggesting that the *miR-195/497* cluster functioned as tumor suppressors in BC. Regarding the genes targeted by the *miR-195/497* cluster, the TargetScan algorithm showed that 6,730 genes were putative *miR-195/497* targets, and 113 significantly enriched signaling pathways were identified in this analysis. The “Pathways in cancer” category was the most enriched, involving 104 candidate target genes. Gene expression data revealed that 27 of 104 candidate target genes were actually upregulated in BC clinical specimens. Luciferase reporter assays and Western blotting demonstrated that *BIRC5* and *WNT7A* were directly targeted by *miR-195/497*. In conclusion, aberrant expression of clustered miRNAs was identified by deep sequencing, and downregulation of *miR-195/497* contributed to BC progression and metastasis. Tumor suppressive miRNA-mediated cancer pathways provide new insights into the potential mechanisms of BC oncogenesis.

## Introduction

In developed countries, bladder cancer (BC) is the fifth most commonly diagnosed tumor and the second most common cause of death in patients with genitourinary tract malignancies. In the United States, there are more than 73,000 new cases and 14,000 deaths annually [1]. In Japan, the number of new BC patients was estimated at 17,461 in 2007 and the number of deaths was estimated at 7,008 in 2011 [2].

BCs can be classified into two categories, non-muscle-invasive tumors and muscle-invasive tumors. Although 70%–80% of patients are diagnosed with non-muscle-invasive tumors, high recurrence rates (50%–70%) are observed in these patients. Moreover, among recurrent cases, 15% of BCs progress to muscle-invasive disease [3]. The five-year survival rate for patients with non-muscle-invasive BC is close to 90%, whereas that of patients with muscle-invasive BC is approximately 60% [4]. Furthermore, nearly 80% of patients with lymph node metastases die within the first five years [5]. Since most clinical trials of chemotherapeutics for advanced BC have shown limited benefits, new prognostic markers and effective treatment strategies based on current cancer-genome analyses are necessary.

The discovery of non-coding RNA in the human genome was an important conceptual breakthrough in the post-genomic sequencing era [6]. Improved understanding of non-coding RNA is necessary for continued progress in cancer research. miRNAs constitute a class of small, non-coding RNA molecules, 19–22 nucleotides in length, that modulate gene expression. Regulation is achieved through imperfect pairing with target messenger RNAs (mRNAs) of protein-coding genes and transcriptional or post-transcriptional regulation of their expression [7]. Currently, 2,042 human mature miRNAs are registered at miRBase release 20.0 [http://microrna.sanger.ac.uk/]. A growing body of evidence indicates that miRNAs also contribute to the initiation, development and metastasis of various types of cancers. Many human cancers show aberrant expression of miRNAs that can function either as tumor suppressors or oncogenes [8]. Therefore, identification of aberrantly expressed miRNAs is the first step toward elucidating miRNA-mediated oncogenic pathways in human cancers.

The development of high-throughput, deep sequencing technology has rapidly uncovered novel information about miRNAs. Deep sequencing analysis seems to be superior to microarray- or PCR-based methods that are limited to known miRNAs and usually do not contain the full list of known miRNAs sequences. Deep sequencing analysis will become the gold standard method for comprehensive miRNA analysis in cancer genomics. miRNA expression signatures of BC obtained by deep sequencing technology have led to four recent publications [9]–[12] and this study constitutes the fifth report. A total of ten small RNA libraries were sequenced (five bladder carcinomas and five matched, histologically normal samples of urothelia), leading to 13,190,619 to 18,559,060 clean small RNA reads in this analysis. We detected 933 known miRNAs and 17 new miRNA candidates in our series of samples.

Some miRNAs are located in close proximity to one another on the human genome, and are therefore termed clustered miRNAs. In the human genome, 247 human miRNAs have been found to be clustered at 64 sites at inter-miRNA distances less than 5000 bp [13]. The *miR-15a/16* cluster, for example, is well known to act as a tumor suppressor by targeting multiple oncogenes, including *BCL2*, *MCL1*, *CCND1* and *WNT3A* [14]–[16], whereas the *miR-17/92* cluster is recognized as an oncogene [17]. We previously reported that *miR-1-1/133a-2* and *miR-1-2/133a-1* formed clusters in different chromosomal loci in the human genome, 20q13.33 and 18q11.2, respectively, and these clusters function as tumor suppressors, targeting several oncogenes in human cancers, including BC [18]–[24]. The *miR-143/145* cluster is located at the 5q32 locus and was also reported as a tumor suppressive miRNA cluster in BC [25]–[28]. We selected 60 downregulated miRNAs in the BC signature and investigated their chromosomal loci. Interestingly, among the 60 miRNAs, we found that several formed clusters, such as *miR-1/133a*, *miR-206/133b*, *let-7c/miR-99a*, *miR-143/145* and *miR-195/497*.

In this study, we hypothesized that the *miR-195/497* cluster functioned as a tumor suppressor through targeting of several oncogenic genes involved in specific cancer-related pathways in BC. Based on this hypothesis, we performed gain-of-function studies in microRNAs transfectants and also attempted to identify novel *miR-195/497* cluster-mediated molecular targets and pathways by *in silico* analysis. Understanding the role of miRNAs will provide an effective and promising strategy for miRNA-associated evidence-based therapeutics for the treatment of BC.

## Results

### Sequencing and annotation of small RNAs

Ten small RNA libraries (from five BCs and five NBEs) were sequenced by using deep sequencing technology. The patients' characteristics are shown in Table 1. Initially, 13,905,124 to 18,938,856 raw sequence reads were produced for the libraries. After filtering and trimming of the reads for low quality and adaptors, from 13,190,619 to 18,559,060 clean small RNA reads were obtained (Table 2). The distribution of nucleotide lengths of clean small RNA reads varied from 16 to 38 nucleotides in each library. The most abundant length was 22 nucleotides (Figure S1). All of the high-quality clean reads larger than 18 nucleotides were mapped to the human genome and these genome-matched reads were divided into different categories of small RNAs according to their biogenesis and annotation (Table 3). Both BCs and NBEs contained multiple and heterogeneous small RNAs species that included miRNA, degradation fragments of non-coding RNAs (tRNA, rRNA, snRNA, snoRNA, etc.), genomic repeats, mRNAs, or unclassified RNAs. The most abundant RNA category from each library was miRNA. The sequence data from this study were deposited in DDBJ Sequence Read Archive (accession number; DRA001043).

**Table 1:** Patient characteristics for deep sequencing.

No. of samples	Age	Gender	Pathological type	Stage	Grade
#1	65	M	UC	T2	3
#2	76	M	UC>SCC	T3	3
#3	91	M	UC	T2	2
#4	92	F	UC	T2	2
#5	83	M	UC	T2	3
#6	73	M	Normal	-	-
#7	56	M	Normal	-	-
#8	63	M	Normal	-	-
#9	69	M	Normal	-	-
#10	74	M	Normal	-	-

**Table 2:** Details of sequence reads in BCs (#1 – #5) and NBEs (#6 – #10).

BC samples	#1		#2		#3		#4		#5	
	Count	(%)	Count	(%)	Count	(%)	Count	(%)	Count	(%)
Total reads	15818359		14913503		14046476		14401456		13905124	
High quality reads	15591657	(100)	14650428	(100)	13789073	(100)	14147571	(100)	13639995	(100)
Clean reads	15322668	(98.27)	14462412	(98.72)	13358689	(96.88)	13493069	(95.37)	13190619	(96.71)
3′adapter null	14344	(0.09)	35072	(0.24)	14363	(0.10)	15721	(0.11)	19524	(0.14)
Insert null	16418	(0.11)	15790	(0.11)	14501	(0.11)	30140	(0.21)	31037	(0.23)
5′adapter contaminants	118787	(0.76)	74705	(0.51)	108819	(0.79)	265131	(1.87)	208178	(1.53)
Smaller than 18 nt	119154	(0.76)	61981	(0.42)	292234	(2.12)	343268	(2.43)	189949	(1.39)
Poly A	286	(0.00)	468	(0.00)	467	(0.00)	242	(0.00)	688	(0.01)

NBE samples	#6		#7		#8		#9		#10	
	Count	(%)	Count	(%)	Count	(%)	Count	(%)	Count	(%)
Total reads	16397994		17352333		17308263		17235561		18938856	
High quality reads	16145175	(100)	17105236	(100)	17042790	(100)	16987399	(100)	18695703	(100)
Clean reads	16004721	(99.13)	16985386	(99.30)	16886490	(99.08)	16897435	(99.47)	18559060	(99.27)
3′adapter null	7921	(0.05)	6758	(0.04)	7587	(0.04)	7708	(0.05)	11987	(0.06)
Insert null	6991	(0.04)	6070	(0.04)	10697	(0.06)	6330	(0.04)	15325	(0.08)
5′adapter contaminants	40675	(0.25)	46625	(0.27)	46521	(0.27)	34116	(0.20)	36165	(0.19)
Smaller than 18 nt	84612	(0.52)	60017	(0.35)	91274	(0.54)	41449	(0.24)	72922	(0.39)
Poly A	255	(0.00)	380	(0.00)	221	(0.00)	361	(0.00)	244	(0.00)

**Table 3:** Annotation of total small RNA reads in BCs (#1 – #5) and NBEs (#6 – #10).

BC samples	#1		#2		#3		#4		#5	
	Count	(%)	Count	(%)	Count	(%)	Count	(%)	Count	(%)
Total (high quality clean reads)	15322668	(100)	14462412	(100)	13358689	(100)	13493069	(100)	13190619	(100)
exon antisense	4282	(0.03)	4075	(0.03)	3935	(0.03)	3529	(0.03)	10096	(0.08)
exon sense	227411	(1.48)	336813	(2.33)	179263	(1.34)	187273	(1.39)	180167	(1.37)
intron antisense	9126	(0.06)	12959	(0.09)	5808	(0.04)	12527	(0.09)	16709	(0.13)
intron sense	53585	(0.35)	57953	(0.40)	59224	(0.44)	50310	(0.37)	121919	(0.92)
miRNA	10827789	(70.67)	7725323	(53.42)	7742465	(57.96)	8045134	(59.62)	8035424	(60.92)
piRNA	4192	(0.03)	20971	(0.15)	3855	(0.03)	9539	(0.07)	9834	(0.07)
rRNA	1527410	(9.97)	3071330	(21.24)	2367709	(17.72)	2199650	(16.30)	2327091	(17.64)
Repeat	56174	(0.37)	130790	(0.90)	72388	(0.54)	51759	(0.38)	168600	(1.28)
scRNA	20681	(0.13)	106886	(0.47)	19350	(0.14)	69649	(0.52)	43064	(0.33)
snRNA	193055	(1.26)	171246	(1.18)	107841	(0.81)	111721	(0.83)	132285	(1.00)
snoRNA	91822	(0.60)	461726	(3.19)	52568	(0.39)	98987	(0.73)	95634	(0.73)
srpRNA	974	(0.01)	7030	(0.05)	3282	(0.02)	1346	(0.01)	4946	(0.04)
tRNA	146656	(0.96)	307216	(2.12)	160142	(1.20)	240739	(1.78)	239702	(1.82)
Unann	2159511	(14.09)	2048094	(14.16)	2580859	(19.32)	2410906	(17.87)	1805148	(13.69)

NBE samples	#6		#7		#8		#9		#10	
	Count	(%)	Count	(%)	Count	(%)	Count	(%)	Count	(%)
Total (high quality clean reads)	16004721	(100)	16985386	(100)	16886490	(100)	16897435	(100)	18559060	(100)
exon antisense	1097	(0.01)	616	(0.00)	669	(0.00)	489	(0.00)	1156	(0.01)
exon sense	24983	(0.16)	16875	(0.10)	21968	(0.13)	16073	(0.10)	30165	(0.16)
intron antisense	2128	(0.01)	1762	(0.01)	1813	(0.01)	1735	(0.01)	2567	(0.01)
intron sense	11048	(0.07)	6994	(0.04)	9731	(0.06)	6824	(0.04)	14675	(0.08)
miRNA	12046532	(75.27)	12752715	(75.08)	12873067	(76.23)	12731851	(73.35)	13774337	(74.22)
piRNA	1167	(0.01)	1913	(0.01)	1263	(0.01)	2988	(0.02)	3423	(0.02)
rRNA	698130	(4.36)	506956	(2.98)	714262	(4.23)	444588	(2.63)	716902	(3.86)
Repeat	13966	(0.09)	10630	(0.06)	10912	(0.06)	9387	(0.06)	17032	(0.09)
scRNA	12274	(0.08)	14673	(0.09)	8937	(0.05)	21720	(0.13)	22200	(0.12)
snRNA	41453	(0.26)	45731	(0.27)	47270	(0.28)	42717	(0.25)	57247	(0.31)
snoRNA	11875	(0.07)	13100	(0.08)	14569	(0.09)	13230	(0.08)	19526	(0.11)
srpRNA	367	(0.00)	116	(0.00)	109	(0.00)	102	(0.00)	241	(0.00)
tRNA	71943	(0.45)	77194	(0.45)	67050	(0.40)	75340	(0.45)	98803	(0.53)
Unann	3067758	(19.17)	3536111	(20.82)	3114870	(8.45)	3530391	(20.89)	3800786	(20.48)

### Identification of downregulated miRNAs based on miRNA expression signatures of BC

We separated the high quality clean read sequences in each library into two categories, miRNAs or non-coding RNAs using NCBI GenBank data, Rfam data and miRBase. In this study, a total of 933 known miRNAs and 17 candidate new miRNAs were detected from high quality clean read sequences (Tables S1 and S2). The expression levels of the new miRNA candidates were low and the functional roles were not assessed enough (Table S2). For these reasons, we excluded them from the analysis. However, the functional significance of these new miRNA candidates is important and they will be studied in the future. Here, we focused on the 933 known miRNAs and established the miRNA expression signature of BC by deep sequencing. Differentially expressed miRNAs were identified by using the edgeR program with a general linear model. A total of 60 downregulated miRNAs were selected for this analysis (Table 4).

**Table 4:** Downregulated miRNAs in deep sequencing of BC.

MiRNAs	Location	Log2 fold change	FDR (false discovery rate)
*hsa-miR-1*	18q11.2/20q13.33	−6.987	2.179E-21
*hsa-miR-133a*	18q11.2/20q13.33	−6.786	1.731E-19
*hsa-miR-145*	5q32	−5.636	2.699E-16
*hsa-let-7c*	21q21.1	−5.315	3.277E-16
*hsa-miR-133b*	6p12.2	−7.311	1.412E-15
*hsa-miR-143*	5q32	−4.285	6.421E-11
*hsa-miR-139-5p*	11q13.4	−4.532	3.742E-10
hsa-miR-145*	5q32	−4.410	6.912E-10
*hsa-miR-490-3p*	7q33	−6.123	1.456E-09
*hsa-miR-3914*	7q11.23	−8.918	1.757E-09
*hsa-miR-204*	9q21.12	−5.702	3.096E-09
hsa-miR-99a*	21q21.1	−5.359	4.423E-09
*hsa-miR-206*	6p12.2	−5.634	5.317E-09
*hsa-miR-383*	8p22	−4.640	1.441E-08
*hsa-miR-125b*	11q24.1	−4.178	1.757E-08
*hsa-miR-99a*	21q21.1	−4.653	1.757E-08
*hsa-miR-451*	17q11.2	−3.377	8.659E-08
hsa-miR-144*	17q11.2	−3.466	3.562E-07
*hsa-miR-3656*	11q.23.3	−7.629	8.399E-07
*hsa-miR-486-5p*	8p11.21	−3.469	9.104E-07
hsa-miR-218-1*	4p15.31	−5.011	1.170E-06
*hsa-miR-195*	17p13.1	−3.218	1.968E-06
*hsa-miR-139-3p*	11q13.4	−3.343	2.472E-06
*hsa-miR-100*	11q24.1	−3.671	2.796E-06
*hsa-miR-1265*	10p13	−7.399	7.899E-06
hsa-miR-30a*	6q13	−2.753	5.103E-05
*hsa-miR-23b*	9q22.32	−2.517	6.480E-05
*hsa-miR-3622a-5p*	8p21.1	−6.577	7.196E-05
hsa-miR-143*	5q32	−2.852	7.511E-05
*hsa-miR-144*	17q11.2	−3.082	7.520E-05
hsa-miR-125b-2*	21q21.1	−3.622	7.520E-05
*hsa-miR-887*	5p15.1	−3.128	9.511E-05
*hsa-miR-1258*	2q31.3	−4.798	9.890E-05
*hsa-miR-490-5p*	7q33	−5.012	1.035E-04
hsa-miR-30c-2*	6q13	−2.600	1.253E-04
hsa-miR-129*	7q32.1	−3.687	1.265E-04
*hsa-miR-199b-5p*	9q34.11	−2.564	1.487E-04
*hsa-miR-1247*	14q32.31	−3.678	2.101E-04
*hsa-miR-873*	9p21.1	−3.974	2.374E-04
*hsa-miR-497*	17p13.1	−2.632	3.217E-04
*hsa-miR-338-5p*	17q25.3	−3.264	4.608E-04
*hsa-miR-223*	Xq12	−2.772	5.174E-04
*hsa-miR-202*	10q26.3	−3.426	8.948E-04
*hsa-miR-30a*	6q13	−2.197	1.416E-03
*hsa-miR-199a-3p*	19p13.2	−2.128	1.422E-03
*hsa-miR-1298*	Xq23	−3.211	1.566E-03
hsa-miR-202*	10q26.3	−3.196	1.762E-03
*hsa-miR-199b-3p*	9q34.11	−2.128	1.762E-03
hsa-miR-100*	11q24.1	−4.194	2.340E-03
*hsa-miR-130a*	11q12.1	−1.952	3.063E-03
*hsa-miR-3154*	9q34.11	−2.671	4.653E-03
hsa-miR-125b-1*	11q24.1	−2.847	4.859E-03
*hsa-miR-137*	1p21.3	−5.461	5.424E-03
*hsa-miR-337-3p*	14q32.2	−2.450	5.534E-03
*hsa-miR-299-5p*	14q32.31	−2.249	6.200E-03
*hsa-miR-152*	17q21.32	−1.784	7.191E-03
*hsa-miR-199a-5p*	1q24.3/19p13.2	−1.934	7.963E-03
hsa-miR-23b*	9q22.23	−1.818	8.491E-03
*hsa-miR-338-3p*	17q25.3	−2.152	8.952E-03
*hsa-miR-127-3p*	17q25.3	−1.854	9.570E-03

**Figure 1:**
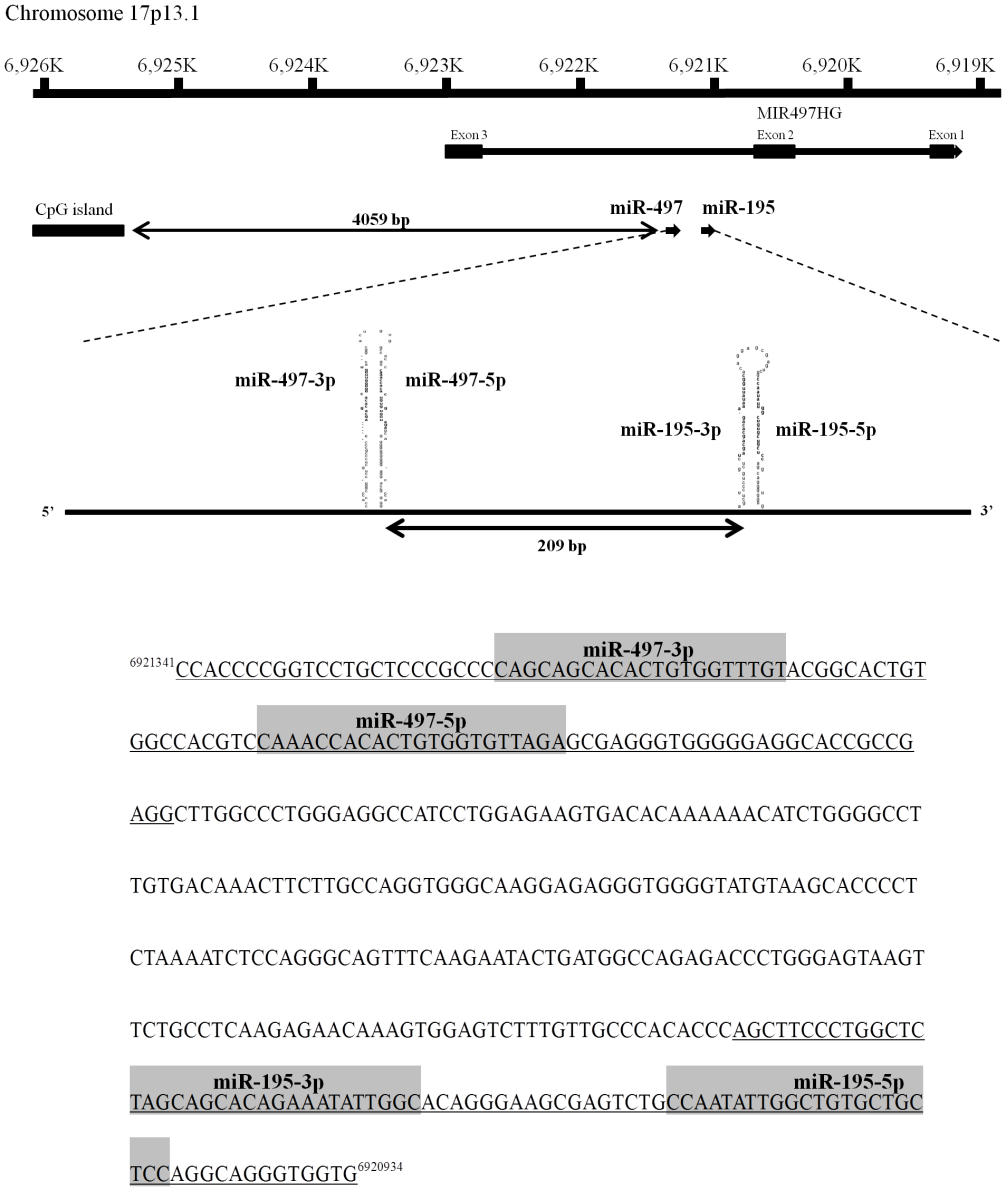
Schematic representation of the human *miR-195*/*497* cluster location. *miR-195* and *miR-497* are in an intron of the *MIR497HG* gene on human chromosome 17q13.1, where they are separated by 209 bp.

Next, we mapped the downregulated miRNAs in the human genome and found several miRNAs that were close together and were termed clustered miRNAs, such as *miR-1/133a, miR-206/133b, let-7c/miR-99a, miR-143/145* and *miR-195/497* (Table 5). Because the role of the *miR-195/497* cluster had not been reported in BC, we focused on it for further studies. As shown in Figure 1, *miR-195* and *miR-497* are located within the same chromosomal region (17p13.1), 209 bp apart.

**Table 5:** Differentially expressed clustered miRNAs, their target mRNAs, and associated pathways in BC.

MiRNA clusters	Expression	Target mRNA	Reference
*miR-195/497*	Down	*GLUT3*	[41]
	Down	*CDK4*	[42]
*miR-1/133a*	Down	*LASP1*	[18]
	Down	*TAGLN2*	[19]
	Down	*PNP/PTMA*	[20]
	Down	*KRT7*	[32]
	Down	*GSTP1*	[33]
	Down	*SRSF9*	[34]
	Down	*EGFR*	[53]
*miR-143/145*	Down	*FSCN1*	[25]
	Down	*ERK5*	[26]
	Down	*CBFB/PPP3CA/CLINT1*	[27]
	Down	*AKT/ILK*	[28]
*miR-99a/let-7c*	Down	*FGFR3*	[54]
*miR-133b/206*	Down	*EGFR*	[55]

### Expression levels of *miR-195* and *miR-497* in clinical samples

**Figure 2:**
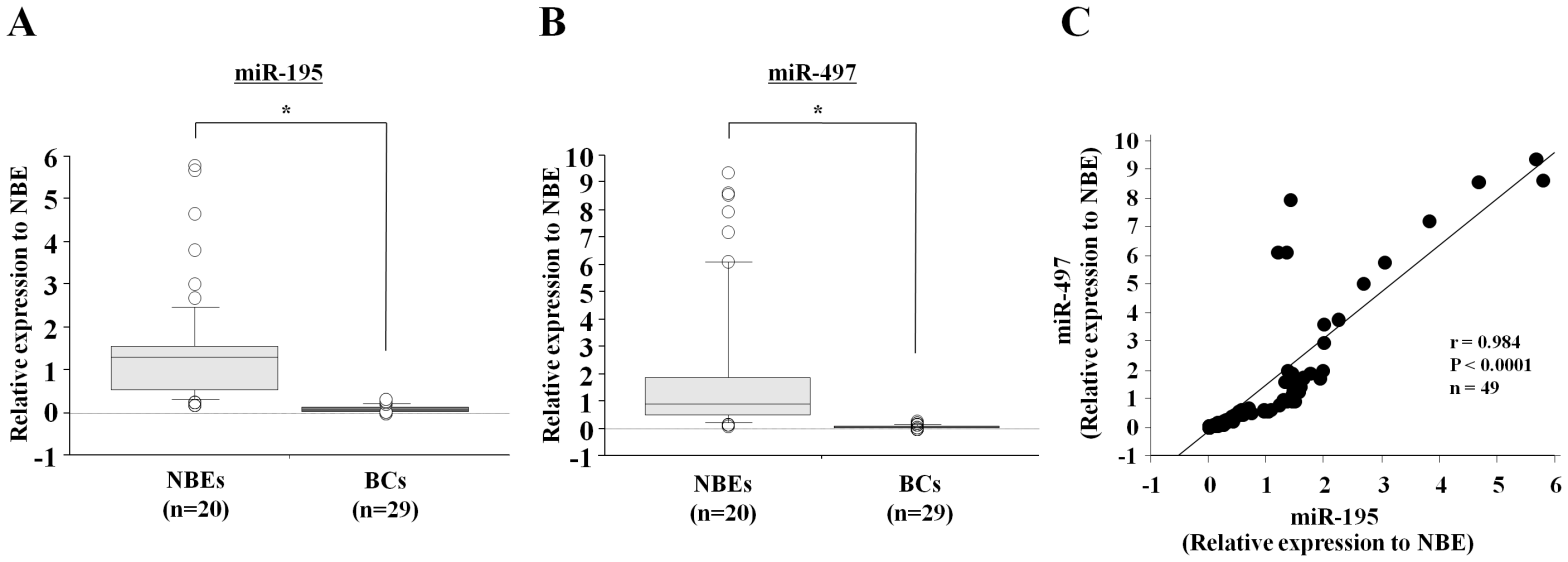
Expression levels of *miR-195* and *miR-497* in BC clinical specimens, and noncancerous bladder tissues. A, B) The expression of *miR-195* and *miR-497* was significantly lower in 20 clinical BC specimens than in 29 adjacent noncancerous specimens. *RNU48* was used as the internal control. *, P<0.0001. C) Significant positive correlation between miRNA expression patterns of *miR-195* and *miR-497* (rank test Spearman's correlation coefficient r = 0.984, P<0.0001).

To validate the expression levels of *miR-195* and *miR-497* in clinical samples, we subjected 29 BCs and 20 NBEs to stem-loop RT-PCR. We found that the expression levels of *miR-195* and *miR-497* in BCs were significantly lower than those in NBEs (*miR-195*: 0.083±0.078 and 1.367±1.178, P<0.0001; *miR-497*: 0.056±0.058 and 1.928±2.425, P<0.0001) (Figure 2A, B). Interestingly the Spearman correlation coefficient obtained with the rank test revealed a significant positive correlation between the expression levels of *miR-195* and *miR-497* in the clinical samples (r = 0.984, P<0.0001) (Figure 2C).

### Effect of *miR-195* and *miR-497* transfections on cell proliferation, invasion, and migration activities of BC cell lines

**Figure 3:**
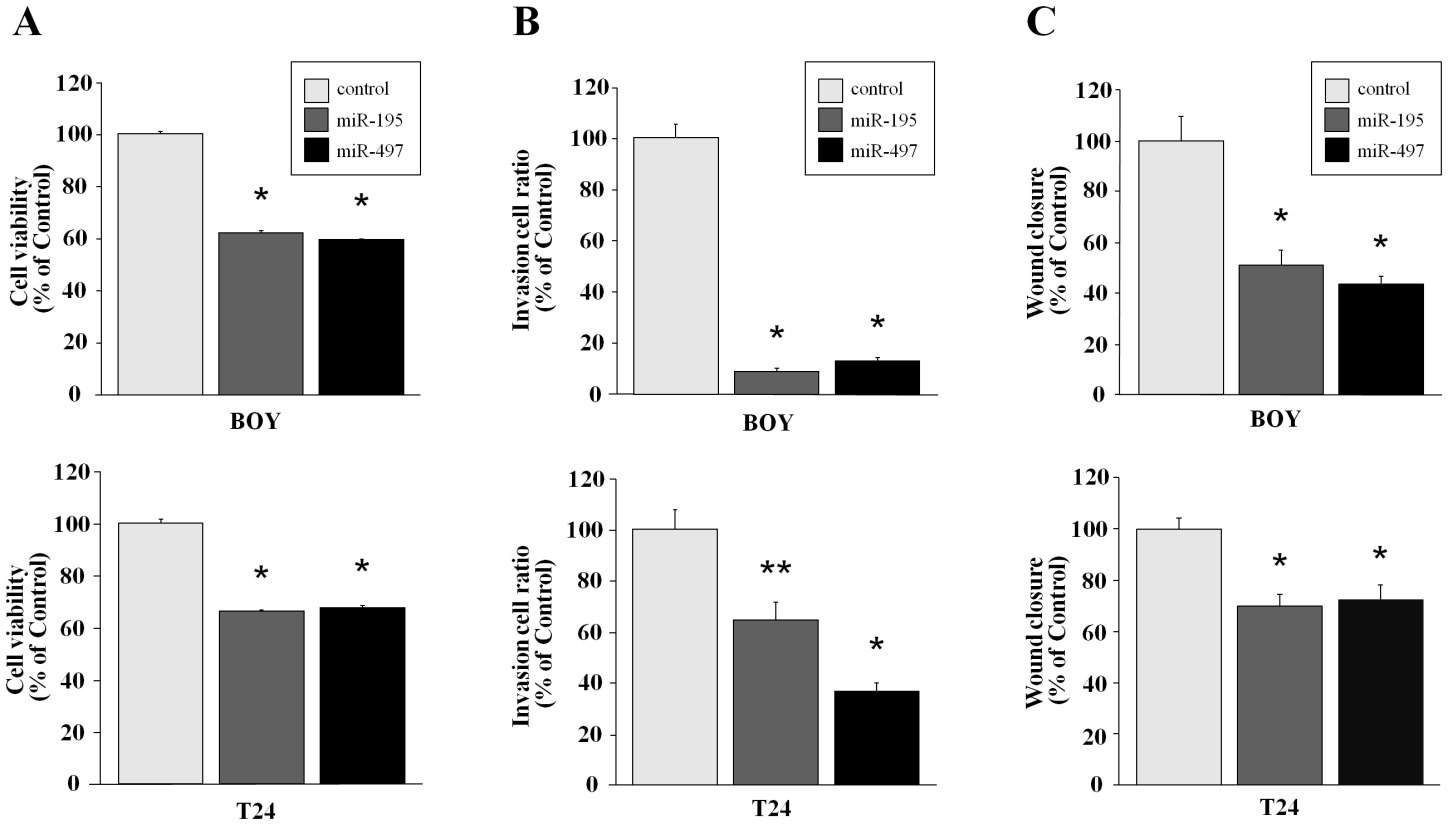
Effect of mature *miR-195* and *miR-497* transfection of BC cell lines (BOY, T24). Gain-of-function studies were performed by using *miR-195* and *miR-497*-transfected BOY and T24 in comparison with the miR-control transfectants. A) Cell proliferation determined by the XTT assay. B) Cell invasion activity demonstrated by the Matrigel invasion assay. C) Cell migration activity determined by the wound healing assay. *, P<0.0001; **, P = 0.009.

To investigate the functional roles of these miRNAs, we performed gain-of-function studies using miRNA transfectants. The XTT assay showed significant inhibition of cell proliferation in *miR-195* and *miR-497* transfectants (BOY and T24) in comparison with the miR-control transfectants (percentage of cell viability for BOY: 61.7±1.4, 59.1±0.9, and 100.0±2.2, respectively, P<0.0001; and, for T24: 66.0±0.9, 67.7±1.2, and 100.0±2.8, respectively, P<0.0001) (Figure 3A). The Matrigel invasion assay demonstrated that invading cell numbers were significantly decreased in both *miR-195-* and *miR-497*-transfected BOY and T24 cell lines in comparison with the controls (percentage of cell invasion for BOY: 8.7±3.1, 13.4±1.7, and 100±12.3, respectively, each P<0.0001; and, for T24: 64.7±16.2, 36.5±7.5, and 100±18.7, respectively, P = 0.009 for the *miR-195* transfectant vs. control, P<0.0001 for the *miR-497* transfectant vs. control) (Figure 3B). The wound healing assay demonstrated significant inhibition of cell migration in both *miR-195-* and *miR-497*-transfected BOY and T24 cells in comparison with the controls (percentage of wound closure for BOY: 51.2±15.5, 43.9±8.3, and 100±28.7, respectively, each P<0.0001; and for T24: 70.1±11.4, 72.3±18.8, and 100±12.6, respectively, each P<0.0001) (Figure 3C).

### Predicted target genes and cancer pathways of the *miR-195/497* cluster

**Figure 4:**
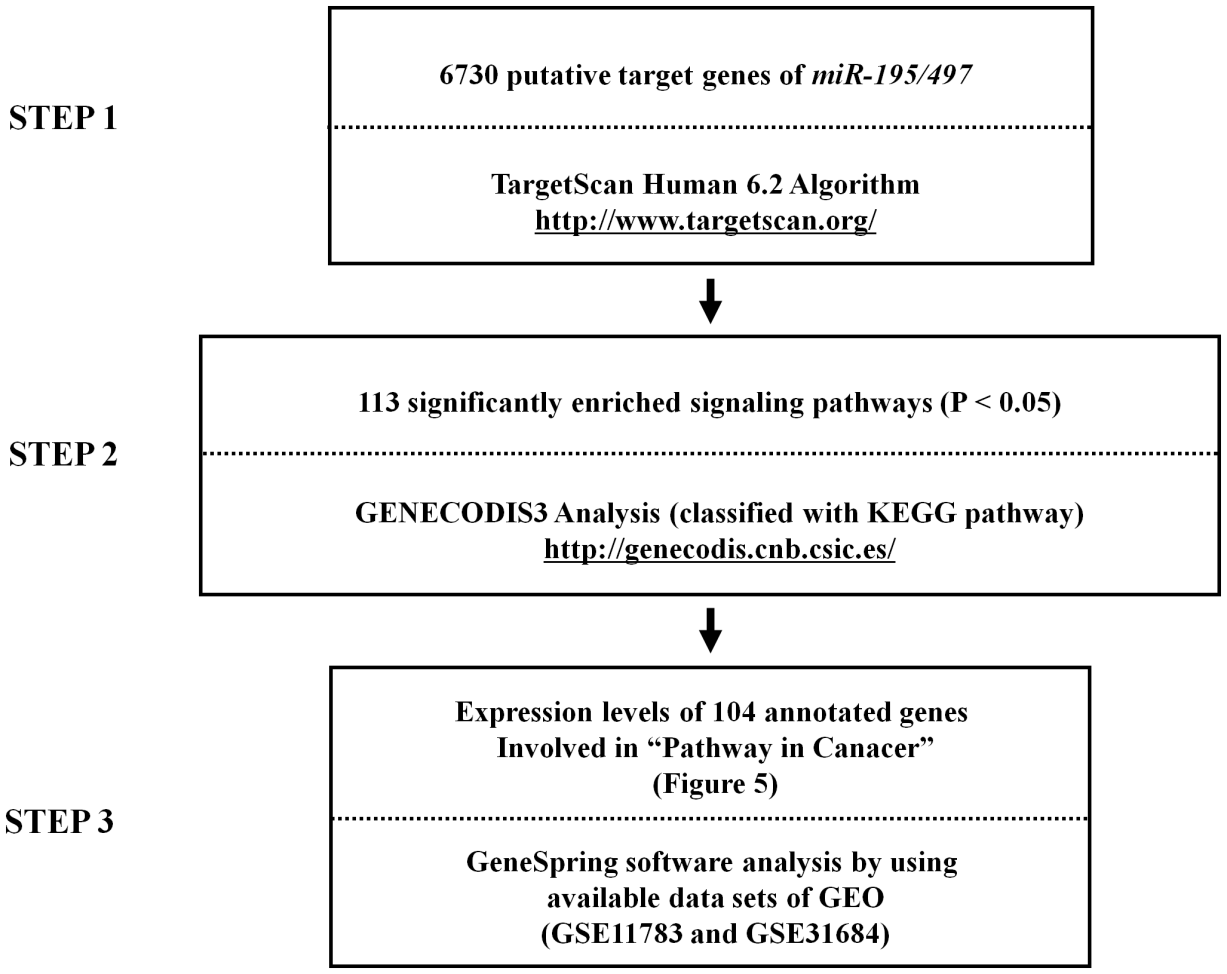
Identification of *miR-195/497* cluster-mediated molecular pathways and putative molecular targets. A total of 6730 genes were identified by the TargetScan algorithm. Then, 113 significantly enriched signaling pathways were identified by using the KEGG and GeneCodis3 programs. Among them, the top 21 enriched pathways are shown in Table S3. The expression levels of 104 genes in the top enriched pathway (Pathways in cancer) were finally evaluated. Among them, 27 putative target genes were upregulated in BC clinical specimens based on expression data of GEO database (accession numbers; GSE11783 and GSE31684) (Table S4).

To explore the biological functions of the *miR-195/497* cluster, we performed *in silico* analyses using two independent algorithms, TargetScan, and GeneCodis3, and public microarray expression data approved by GEO. The workflow for *in silico* analysis is shown in Figure 4. At first, the TargetScan database indicated that 6,730 genes were predicted targets of the *miR-195/497* cluster, which shared the same seed sequence ‘GCUGCU’ in their 3′-untranslated regions (3′UTR).

**Figure 5:**
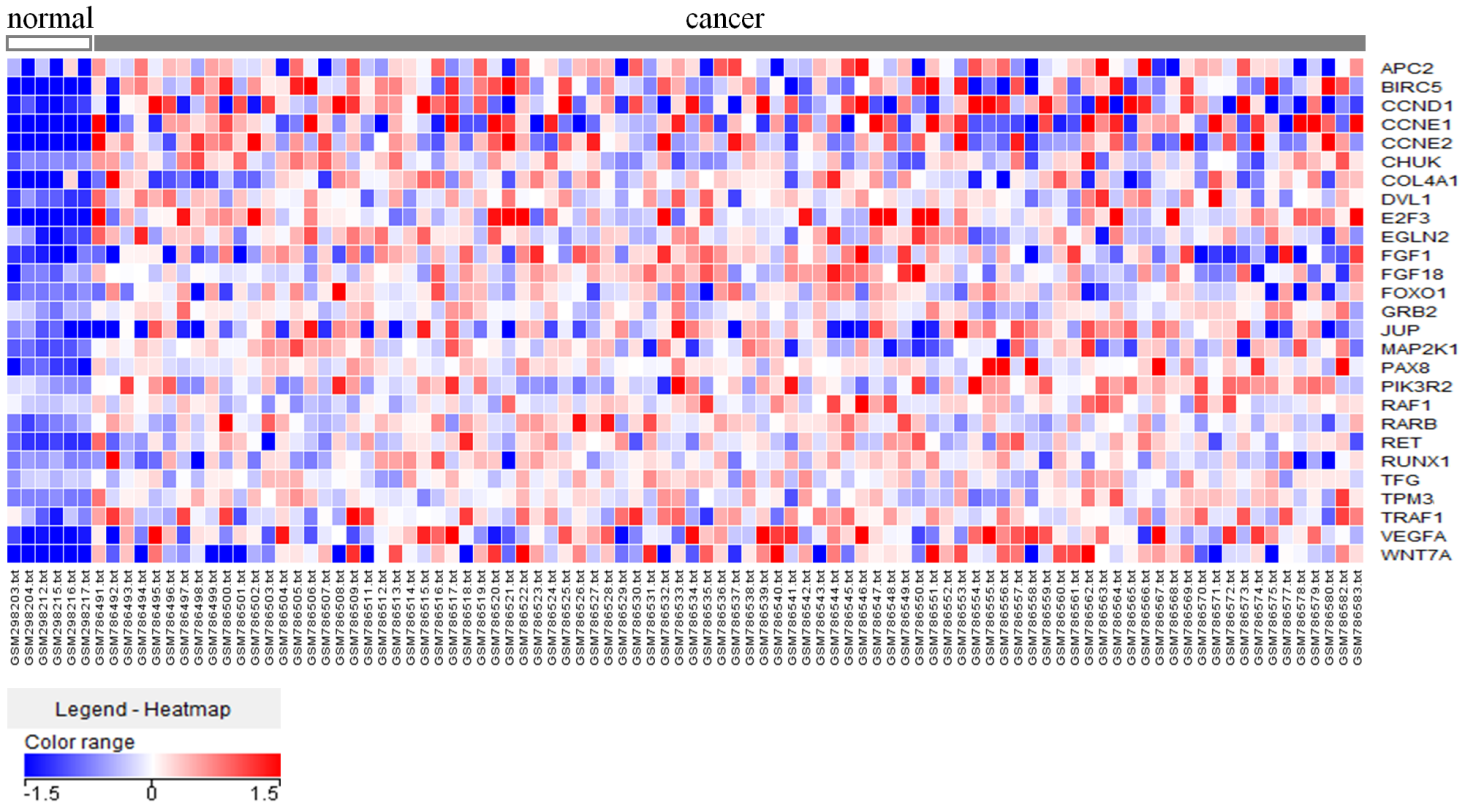
Genes upregulated by the *miR-195/497* cluster in BC specimens. Heat map diagram shows the expression of 27 candidate genes involved in “Pathways in cancer” in 90 BC specimens and six adjacent noncancerous bladder specimens.

Based upon the KEGG pathway classification, these genes were implicated in 113 pathways, and “Pathways in cancer” was the most significantly enriched (Table S3). A total of 104 genes were involved in this pathway. We searched the oncogenes targeted by the *miR-195/497* cluster based on the hypothesis that putative oncogenes would be upregulated in clinical specimens due to downregulation of tumor suppressive *miR-195/497* miRNAs. By using GEO database analysis, 27 genes were selected as *miR-195/497*-regulated oncogenic genes in BC (Figure 5 and Table S4). To confirm this analysis, we selected the top two upregulated genes (*BIRC5* and *WNT7A*), and validated whether these miRNAs were regulated by *miR-195/497* (below).

### *BIRC5* and *WNT7A* were directly regulated by the *miR-195/497* cluster in BC cells

**Figure 6:**
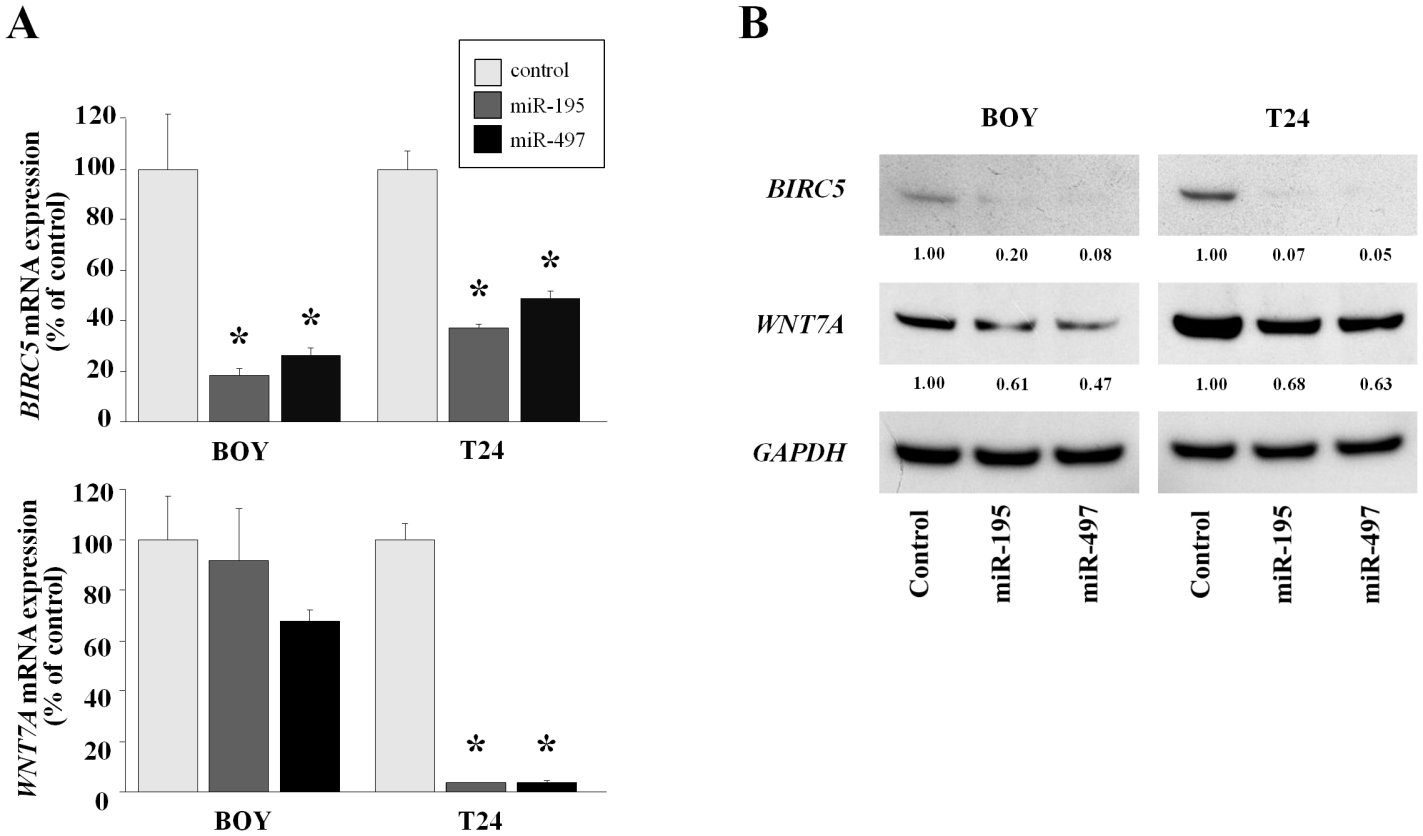
*BIRC5* and *WNT7A* mRNA and protein expression levels were suppressed by *miR-195* and *miR-497* transfection in comparison with the miR-control transfection in BC cell lines (BOY and T24). A) *BIRC5* and *WNT7A* mRNA expression 72 h after transfection with *miR-195*, *miR-497*, and the miR-control. *GUSB* expression was used for normalization. B) *BIRC5* and *WNT7A* protein expression 72 h after transfection with *miR-195*, *miR-497*, and the miR-control. *GAPDH* was used as a loading control. The ratio of *BIRC5* or *WNT7A* to *GAPDH* expression was evaluated using ImageJ software (ver. 1.43; http://rsbweb.nih.gov/ij/index.htmL). *P<0.01.

The mRNA and protein expression levels of *BIRC5* and *WNT7A* were downregulated in *miR-195-* and *miR-497-*transfected BOY and T24 cells in comparison with control transfectants (Figure 6A, B).

**Figure 7:**
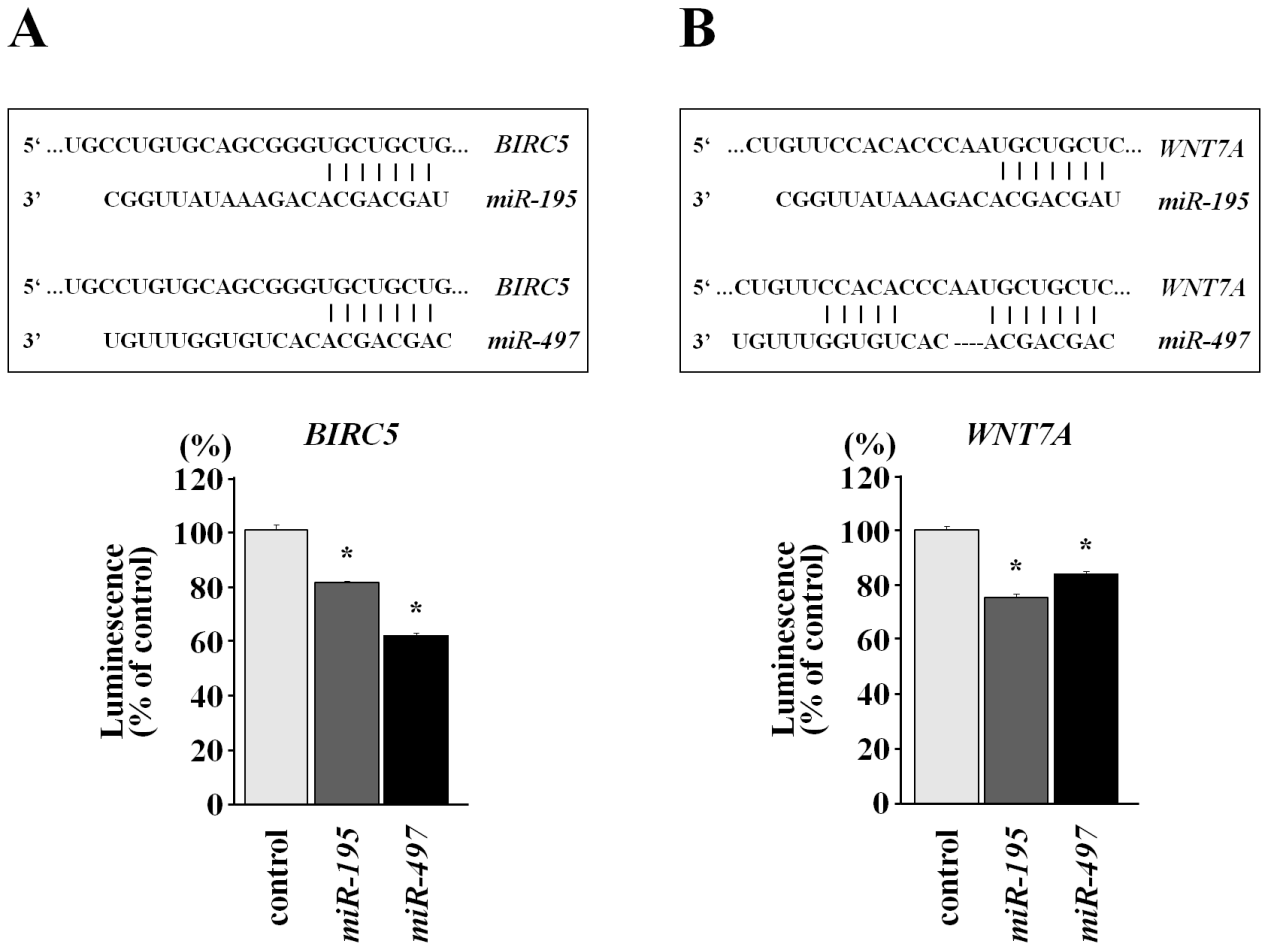
*BIRC5* and *WNT7A* as target genes of the *miR-195/497* cluster in BC. Luminescence intensity was measured in *miR-195-* and *miR-497-*transfectants in comparison with the miR-control transfectant. A) *miR-195/497* cluster binding sites in the 3′-UTR of *BIRC5* mRNA. Luciferase reporter assay vector used the encoding 3′-UTR region of *BIRC5* including putative *miR-195/497* target sites. *Renilla* luciferase values were normalized to firefly luciferase values. *P<0.01. B) *miR-195/497* cluster binding sites in the 3′-UTR of *WNT7A* mRNA. Luciferase reporter assay vector is the encoding 3′-UTR region of *WNT7A* including putative *miR-195/497* target site. *P<0.01.

We next performed luciferase reporter assays to determine whether *BIRC5* and *WNT7A* mRNAs had functional target sites for *miR-195* and *miR-497* in BC. We used a vector encoding the 3-′UTRs of *BIRC5* and *WNT7A* mRNAs, including putative target sites for *miR-195* and *miR-497* (positions 198–204 and 197–203, respectively; Table S5). We found that the luminescence intensity was significantly decreased in the presence of the target site of *BRIC5* by *miR-195-* and *miR-497-*transfectants (Figure 7A). We obtained the same result with a luciferase reporter assay for the *WNT7A* gene (Figure 7B). These data suggested that *miR-195* and *miR-497* bound directly to specific sites in the 3′-UTR of both *BRIC5* and *WNT7A* mRNAs.

## Discussion

It is believed that miRNAs regulate the expression of approximately 30% of all genes in the human genome [29]. In normal cells, the interaction of miRNAs and target messenger RNAs (mRNAs) is tightly regulated, whereas this regulation is often missing in cancer cells. A growing body of evidence suggests that miRNAs are aberrantly expressed in many human cancers and that they play significant roles in the initiation, development, and metastasis of these cancers [30]. Therefore, identification of aberrantly expressed miRNAs is an important step in the analysis of human oncogenesis. The latest analytical method in genomics, deep sequencing technology, has the potential to identify novel tissue-specific miRNAs including those in human cancer tissues [31]. Deep sequencing technology is currently the gold standard for comprehensive analysis of human miRNAs. In this study, deep sequencing identified a large set of miRNAs that are differentially expressed between BC and normal epithelia.

Determining the expression signatures of miRNAs can rapidly and reliably reveal miRNAs that are aberrantly expressed in cancers. Therefore, we previously analyzed miRNA expression signatures through the use of PCR-based arrays and searched for tumor suppressive miRNAs in BC. In this way, we successfully identified tumor suppressive miRNAs such as *miR-1*, *miR-133a*, *miR-145* and *miR-218* [18]–[20], [25], [32]–[36]. We compared our previous results with the deep sequencing signatures described herein, allowing us to include the original data set with the new signature. To date, there have been four studies of BC miRNA expression signatures by deep sequencing analyses [9]–[12]. In published data, *miR-1*, *miR-145*, *miR-143*, *miR-125b*, and *miR-100* have been listed as the most frequently downregulated miRNAs [9]–[12], data confirmed by our own signature. This fact supports the effectiveness of the deep sequencing signature, and it suggests that there are novel tumor suppressive miRNAs in these new data. The advantage of this strategy is to be able to identify new miRNA candidates in BC that have not been previously reported. We found 17 such sequences that might constitute new miRNA candidates. However, they were detected at very low levels of expression in comparison with known miRNAs in both cancer and benign tissues. The functional significance of these new miRNA candidates is unknown, and it will be important in the future to examine the relationship between these candidate miRNAs and BC oncogenesis. In the current study, we did not use tissue micro-dissection, therefore the precise proportion of epithelial cells in the normal samples and the precise proportion of tumor cells in the tumor samples are unknown. However, the thin-flap of normal bladder mucosa was consisted mostly of NBE cells, whereas tumor samples were constituted primarily of cancerous epithelial cells. Therefore, we believe that minor contamination of other cells was not significant and did not affect expression levels of the microRNAs.

MicroRNAs can be categorized into families based on their sequences or into clusters based upon their genomic loci. Clustered miRNAs are transcribed coordinately and have similar functions regulating the same targets [37]. For example, expression of the *miR-1/133a* cluster frequently reduced several types of cancers including BC. This cluster functions as a tumor suppressor targeting the same oncogenic genes such as *TAGLN2* and *PNP* [18]–[23]. Similar examples have been reported with other clustered miRNAs such as *miR-15a/16* and *miR-143/145* [14]–[16], [25]–[28]. Thus, these studies showed that clustered miRNAs might work in combination to accomplish their function throughout the same biological processes and the same targeted genes. With regard to the *miR-195/497* cluster, it has been shown that *miR-195* was downregulated in several cancers [38]–[40] and inhibited glucose uptake, proliferation, and cell cycle by targeting *GLUT3* and *CDK4* in BC [41], [42]. It has also been demonstrated that m*iR-497* was downregulated and functioned as a tumor suppressor by targeting *BCL2* in several cancers [43], [44]. The *miR-195/497* cluster also functioned as a tumor suppressor by targeting *RAF1/CCND1* in breast cancer [45]. To validate these past reports, we evaluated the expression levels of *miR-195* and *miR-497* in BC clinical specimens and we confirmed that *miR-195* and *miR-497* were significantly downregulated in tumor tissues. Next, we investigated the functional significance of *miR-195* and *miR-497* in BC using two cell lines, BOY and T24. Restoration of mature *miR-195* or *miR-497* in cancer cells revealed significant inhibition of cancer cell proliferation, invasion and migration. Our present data and previous studies suggest that the *miR-195/497* cluster does indeed function as a tumor suppressor in BC.

miRNAs are unique in their ability to regulate many protein coding genes. Bioinformatic predictions indicate that miRNAs regulate more than 30% of protein coding genes [29]. In cancer cells, normal miRNA - mRNA networks are disrupted by aberrantly expressed miRNAs. We have taken the position that identification of novel cancer pathways and responsible target genes regulated by the tumor suppressive *miR-195/497* cluster is an important first step in understanding BC oncogenesis. Based on this view, we identified molecular pathways and target oncogenes that were regulated by the *miR-195/497* cluster using genome-wide gene expression data and *in silico* analysis. The *miR-195/497* cluster belongs to the *miR-15/16/195/424/497* family that shares the same 3′-UTR binding seed sequence (AGCAGCA). The TargetScan database identified 6,730 genes that were candidate targets of the *miR-195/497* cluster. KEGG pathway analysis indicated that those genes were implicated in 113 pathways, including “Pathways in cancer”, “MAPK signaling”, “Endocytosis”, “Insulin signaling” and “Wnt signaling”.

Among these pathways, we focused on “Pathways in Cancer” because of their direct relevance. According to our hypothesis, target genes of *miR-195/497* should be upregulated in cancer cells. When we examined the expression levels of 104 genes in the “Pathways in cancer” group, 27 genes were upregulated in BC clinical specimens, suggesting that these genes were candidate *miR-195/497* targets and functioned as oncogenes in BC. To confirm the reliability of our *in silico* analysis of potential target genes, we checked whether *BIRC5* and *WNT7A* genes were in fact regulated by the *miR-195/497* cluster in BC. Our data demonstrated that *BIRC5* and *WNT7A* were directly regulated by the *miR-195/497* cluster in BC. *BIRC5* is a member of the inhibitor of apoptosis (IAP) family preferentially expressed by many cancers including BC [46]. It can be measured in urine at mRNA and protein levels, and urine *BIRC5* was reported as a diagnostic biomarker for BC [47]. Recent studies from other groups have shown that several microRNAs such as *miR-542-3p*, *miR-218*, *miR-708* and *miR-203* directly inhibited *BIRC5* expression in different types of cancer [48]. Our study is the first to suggest that *BIRC5* might be regulated by the *miR-195/497* cluster in BC cells. Therefore, our strategy of identifying novel tumor-suppressive, miRNA-regulated molecular targets/pathways in BC is an effective approach for miRNA-cancer research.

## Conclusions

In summary, we constructed miRNA expression signatures of clinical BC specimens using deep sequencing as a gold standard method. A total of 60 miRNAs were significantly reduced in BC specimens. We identified several downregulated miRNAs that were located within the same genomic region constituting a miRNA cluster, including *miR-1/133a*, *miR-143/145*, *miR-99a/let-7c*, *miR-195/497 and miR-144/451a*. Downregulation of the *miR-195/497* cluster was a frequent event in BC clinical specimens. Restoration of these miRNAs significantly inhibited cancer cell proliferation, migration and invasion, suggesting that *miR-195/497* function as tumor suppressors in BC. Our analysis of the *miR-195/497* cluster suggested that it regulated putative cancer pathways and oncogenic genes. These findings will contribute to the analysis of BC oncogenesis. Identification of the tumor-suppressive *miR-195/497* cluster in human BC could provide new information on potential therapeutic targets in the treatment of BC.

## Materials and Methods

### Clinical specimens and cell culture

Tissue specimens for miRNA screening using deep sequencing were obtained from five BC patients and five NBEs. BC patients had undergone cystectomy or transurethral resection of bladder tumors (TUR-BT) at Kagoshima University Hospital between 2003 and 2010. Five NBEs were derived from patients with non-BC diseases. No contamination of the cancer cells was detected by pathologists. Another series of 29 BCs and 20 NBEs were obtained from Kagoshima University Hospital between 2003 and 2010. This group was subjected to real-time RT-PCR to evaluate miRNA expression levels. The samples were staged in accordance with the tumor-node-metastasis classification system of the American Joint Committee on Cancer/Union Internationale Contre le Cancer (UICC) and histologically graded [49]. Written informed consent was obtained from all patients and our study was approved by the Bioethics Committee of Kagoshima University. The patients' backgrounds and clinicopathological characteristics are summarized in Tables 1 and 6.

**Table 6:** Patient characteristics for real-time RT-PCR.

Bladder cancer (BC)	
Total number	29
Median age (range)	74 (56–91) years
Gender	
Male	23
Female	6
Stage	
pTa	3
pT1	12
pT2	6
pT3	2
pT4	2
Unknown	4
Grade	
G1	0
G2	15
G3	14
Unknown	0
Operation	
TUR-BT	18
Total cystectomy	7
Partial cystectomy	1
Nephroureterectomy	2
Ureterectomy	1
Normal bladder epithelium (NBE)	
Total number	20

We used two human BC cell lines: BOY, which was established in our laboratory from an Asian male patient, 66-years old, who was diagnosed with stage III BC with lung metastasis; and, T24, which was invasive and obtained from the American Type Culture Collection. These cell lines were maintained in minimum essential medium (MEM) supplemented with 10% fetal bovine serum in a humidified atmosphere of 5% CO_2_ and 95% air at 37°C.

### Tissue collection and RNA extraction

Tissue samples were immersed in RNAlater (QIAGEN, Valencia, CA, USA) and stored at −20°C until RNA extraction was conducted. Total RNA, including miRNA, was extracted from frozen fresh tissues using the mirVana™ miRNA isolation kit (Ambion, Austin, TX, USA) following the manufacturer's protocol. The integrity of the RNA was checked with the RNA 6000 Nano Assay Kit and a 2100 Bioanalyzer™ (Agilent Technologies, Santa Clara, CA, USA).

### Small RNA library preparation and profiling by deep sequencing

Total RNAs from five BCs and five NBEs were subjected to deep sequencing. The experimental process included the following steps: small RNA isolation, cDNA library preparation, and sequencing. Total RNA of each sample was size-fractionated on a 15% PAGE gel, and an 18–30 nucleotide fraction was collected. The 5′-RNA adapter was ligated to the RNA with T4 RNA ligase. Ligated RNA was size-fractionated, and the 36–50 nucleotide fraction was excised. The 3′-RNA adapter was subsequently ligated to precipitated RNA using T4 RNA ligase. Ligated RNA was size-fractionated, and the 62–75 nucleotide fraction (small RNA+adaptors) was excised. Small RNAs ligated with adaptors were subjected to RT-PCR to produce sequencing libraries. PCR products were purified and cDNA libraries, 106–118 bp in length, were collected. Then, cDNA libraries were sequenced by Genome Analyzer IIx (GAIIx) (Illumina Inc., CA, USA) at Beijing Genomics Institute at Shenzhen.

### Construction of the miRNA expression signature in BC

The 50 nucleotide sequence tags were produced using HiSeq sequencing. Then, the low quality reads were filtered out according to base quality value, the adaptor sequence was trimmed at the 3′-primer terminus, and the 5′-adaptor contaminants formed by ligation were removed. The high-quality clean reads that were larger than 18 nucleotides were mapped to the human genome using the SOAP program [50]. Small RNA tags were aligned to the miRNA precursor/mature miRNA of corresponding species in miRBase release 20.0. If there were no miRNA information for a particular species in miRBase release 20.0, small RNA tags were aligned to the miRNA precursor/mature miRNA of all plants/animals in miRBase release 20.0. To identify small RNA tags originating from coding exons, repeats, rRNA, tRNA, snRNA, and snoRNA, NCBI GenBank data and Rfam data were used. To identify new miRNA candidate genes, all hairpin-like RNA structures encompassing small RNA tags were identified using Mireap (http://sourceforge.net/projects/mireap/).

Assessment of differentially expressed miRNAs was determined with the edgeR program, version 3.0.8 [51] with the general linear model (GML) method. P-values are adjusted for multiple testing using the Benjamini and Hochberg method [52] and P-values with false discovery rate (FDR)<0.01 are considered as significant.

### Predicting target genes and cancer pathways of *miR-195* and *miR-497*

We applied *in silico* analysis to investigate the biological significance of differentially expressed miRNAs obtained by deep sequencing. The workflow for *in silico* analysis of target genes is shown in Figure 4. First, we identified predicted target genes of *miR-195* and *miR-497* using the TargetScan database [http://www.targetscan.org/]. We performed GeneCodis3 [http://genecodis.cnb.csic.es/] analysis using all candidate genes to identify the biological processes or pathways potentially regulated by *miR-195* and *miR-497* [53]. The GeneCodis3 software assigned a number of the putative target genes to known pathways in KEGG [http://www.genome.jp/kegg/pathway.html]. These data facilitated understanding of miRNA-regulated molecular networks in human cells. We performed gene expression analyses of all candidate genes involved in each of the pathways identified by GeneCodis3 software analysis using microarray expression data, which were approved by the Gene Expression Omnibus (GEO) and were assigned GEO accession numbers (GSE11783 and GSE31684). In the microarray expression data, we examined 90 BCs and six NBEs collected from patients, none of whom had been exposed to chemotherapy before surgery. Statistical analyses were conducted using the Mann Whitney U-test.

### Expression levels of the *miR-195* and *miR-497* cluster in BC specimens

To validate the differentially expressed miRNA and target genes, stem-loop RT-PCR (TaqMan MicroRNA Assay; Applied Biosystems, Foster City, CA, USA) was applied using BC clinical specimens. The procedure for PCR analysis was described previously [32], [34]. TaqMan probes and primers for *miR-195* (assay ID: 000494), *miR-497* (assay ID: 001043), *RNU48* (assay ID: 001006), *BIRC5* (assay ID: Hs04194392_s1), *WNT7A* (assay ID: Hs01114990_m1) and *GUSB* (assay ID: 99999908_m1) were obtained from Applied Biosystems. *RNU48* and *GUSB* were used as internal controls.

### Mature miRNA transfection

As described previously [32], the BC cell lines were transfected with Lipofectamine RNAiMAX transfection reagent (Invitrogen) containing Opti-MEM (Invitrogen) with ten nM Pre-miR™ or *mir*Vana™ miRNA Mimic Negative Control (Applied Biosystems) that are mature miRNA molecules for gain-of function experiments. Cell were seeded in six-well plates for wound healing assays (20×10^4^ per well), in 24-well plates for Matrigel invasion assays (5×10^4^ per well), and in 96-well plates for XTT assays (3,000 per well).

### Cell proliferation, Matrigel invasion, and wound healing assays

Cell proliferation was determined by using an XTT assay (Roche Applied Sciences, Tokyo, Japan) performed according to the manufacturer's instructions. A cell invasion assay was carried out using modified Boyden chambers consisting of Transwell-pre-coated Matrigel membrane filter inserts with eight-µm pores in 24-well tissue culture plates (BD Biosciences, Bedford, MA, USA). MEM containing 10% fetal bovine serum in the lower chamber served as the chemoattractant, as described previously [32]. Cell migration activity was evaluated by wound healing assay. Cells were plated in six-well dishes, and the cell monolayer was scraped using a P-20 micropipette tip. The initial gap length (zero h) and the residual gap length 24 h after wounding were calculated from photomicrographs. All experiments were performed in triplicate.

### Western Blotting

Three days after transfection, protein lysates (50 µg) were separated by NuPAGE on 4%–12% bis-tris gels (Invitrogen) and transferred into polyvinylidene fluoride membranes. Immunoblotting was conducted with diluted (1∶5000) monoclonal anti-*BIRC5* antibodies (catalog number, SAB4100131; Sigma-Aldrich, St Louis, MO, USA), or with diluted (1∶250) monoclonal anti-*WNT7A* (catalog number, HPA015719; Sigma-Aldrich) and anti-*GAPDH* antibodies (catalog number, MAB374; Chemicon, Temecula, CA, USA). The membrane was washed and then incubated with goat anti-rabbit IgG (H+L)-HRP conjugate (Bio-Rad, Hercules, CA, USA). Specific complexes were visualized with an echochemiluminescence (ECL) detection system (GE Healthcare, Little Chalfont, UK), and the protein expression levels of these genes were evaluated by ImageJ software (ver. 1.43; http://rsbweb.nih.gov/ij/index.html).

### Plasmid construction and dual-luciferase reporter assay

miRNA target sequences were inserted between the *Xho*I-*Pme*I restriction sites in the 3′-UTR of the *hRluc* gene in the psiCHECK-2 vector (C8021; Promega, Madison, WI, USA). BOY cells were transfected with 15 ng vector, ten nM miRNAs (Pre-miR™ or *mir*Vana™ miRNA Mimic Negative Control), and one µL Lipofectamine 2000 (Invitrogen) in 100 µL Opti-MEM (Invitrogen). The activities of firefly and *Renilla* luciferases in cell lysates were determined with a dual-luciferase assay system (E1910; Promega). Normalized data were calculated as the ratio of *Renilla*/firefly luciferase activities. Detailed vector sequences are shown in Table S5.

### Statistical analysis

The relationships between two variables and numerical values were analyzed using the Mann-Whitney U test, and the relationship between three variables and numerical values was analyzed using the Bonferroni-adjusted Mann-Whitney U test. The relationship between *miR-195* and *miR-497* expression was analyzed by the Spearman rank correlation. Expert Stat View analysis software (ver. 4; SAS institute Inc., Cary NC) was used in these analyses. In the comparison of three variables, a nonadjusted statistical level of significance of P<0.05 corresponded to the Bonferroni-adjusted level of P<0.0167.

## Supporting Information

File S1 **Tables S1–S5.** **Table S1.** The number of total reads and expressed reads of ‘known miRNAs’ in deep sequencing. **Table S2.** The number of total reads and expressed reads of ‘new miRNA candidates’ in deep sequencing. **Table S3.** Top 21 enriched pathways regulated by *miR-195/497*. **Table S4.** Upregulated target genes involved in the “Pathways in cancer”. **Table S5.** Insert 3′UTR sequence of *BIRC5* and *WNT7A*.
(DOCX)

Figure S1 **The distribution of nucleotide lengths of clean small RNA reads in BC (A-E) and NBE (F-J) libraries.** The distribution of nucleotide lengths of clean small RNA reads varied from 16 to 38 nucleotides in each library. The most abundant length was 22 nucleotides.
(TIF)
