# Supplementary material for: The MicroRNA Expression Signature of Bladder Cancer by Deep Sequencing: The Functional Significance of the miR-195/497 Cluster
Source: PLoS One. 2014 Feb 10;9(2):e84311. doi: 10.1371/journal.pone.0084311 (PMC3919700; doi:10.1371/journal.pone.0084311)
Supplement: File S1 — Tables S1–S5. Table S1. The number of total reads and expressed reads of ‘known miRNAs’ in deep sequencing. Table S2. The number of total reads and expressed reads of ‘new miRNA candidates’ in deep sequencing. Table S3. Top 21 enriched pathways regulated by miR-195/497. Table S4. Upregulated target genes involved in the “Pathways in cancer”. Table S5. Insert 3′UTR sequence of BIRC5 and WNT7A. (DOCX) [file pone.0084311.s001.docx]

| Table S1. The number of total reads and expressed reads of 'known miRNAs' in deep sequencing | | | | | | | | | | |
| --- | --- | --- | --- | --- | --- | --- | --- | --- | --- | --- |
| Sample | #1 | #2 | #3 | #4 | #5 | #6 | #7 | #8 | #9 | #10 |
| Clean reads | 15322668 | 14462412 | 13358689 | 13493069 | 13190619 | 16004721 | 16985386 | 16886490 | 16897435 | 18559060 |
|  |  |  |  |  |  |  |  |  |  |  |
| Number of unique 'known miRNAs' | 776 | 708 | 974 | 760 | 786 | 660 | 659 | 686 | 644 | 693 |
|  |  |  |  |  |  |  |  |  |  |  |
| Expressed reads |  |  |  |  |  |  |  |  |  |  |
| *hsa-let-7a* | 2024912 | 1488768 | 2056014 | 1714011 | 1711408 | 2230744 | 2551635 | 2571824 | 2606289 | 2602907 |
| *hsa-let-7a** | 52 | 30 | 75 | 78 | 105 | 42 | 47 | 52 | 44 | 45 |
| *hsa-let-7a-2** | 0 | 1 | 0 | 0 | 0 | 3 | 3 | 0 | 0 | 1 |
| *hsa-let-7b* | 1248190 | 1066215 | 1599285 | 1295862 | 1213050 | 1356984 | 1679301 | 1408392 | 1753209 | 1642217 |
| *hsa-let-7b** | 32 | 68 | 97 | 245 | 291 | 38 | 56 | 55 | 23 | 34 |
| *hsa-let-7c* | 25014 | 41302 | 7079 | 23714 | 52371 | 955611 | 940322 | 1076343 | 991849 | 1021069 |
| *hsa-let-7c** | 0 | 0 | 0 | 0 | 0 | 1 | 0 | 1 | 0 | 7 |
| *hsa-let-7d* | 66003 | 49379 | 25104 | 50378 | 27459 | 60431 | 62303 | 62029 | 62374 | 67093 |
| *hsa-let-7d** | 734 | 457 | 272 | 1183 | 610 | 516 | 767 | 580 | 575 | 438 |
| *hsa-let-7e* | 168463 | 34363 | 137362 | 257629 | 122633 | 198722 | 225028 | 201353 | 245582 | 219996 |
| *hsa-let-7e** | 43 | 15 | 51 | 103 | 75 | 67 | 92 | 92 | 70 | 90 |
| *hsa-let-7f* | 3094584 | 2278415 | 1845549 | 1193993 | 1798195 | 2419896 | 2306206 | 2666999 | 2523940 | 2688734 |
| *hsa-let-7f-1** | 10 | 10 | 14 | 31 | 27 | 28 | 16 | 20 | 18 | 14 |
| *hsa-let-7f-2** | 6 | 0 | 2 | 0 | 3 | 0 | 0 | 0 | 0 | 0 |
| *hsa-let-7g* | 508427 | 180210 | 207096 | 127900 | 150619 | 210824 | 226276 | 231870 | 186315 | 278772 |
| *hsa-let-7g** | 1 | 0 | 3 | 5 | 3 | 3 | 0 | 0 | 0 | 3 |
| *hsa-let-7i* | 80995 | 46945 | 37441 | 59285 | 86002 | 34701 | 59941 | 50142 | 49628 | 49857 |
| *hsa-let-7i** | 13 | 0 | 5 | 15 | 44 | 14 | 12 | 10 | 0 | 20 |
| *hsa-miR-1* | 35714 | 7539 | 3384 | 15121 | 10242 | 1662377 | 1340627 | 1453457 | 1291553 | 1859136 |
| *hsa-miR-100* | 542 | 2127 | 27 | 515 | 2409 | 9758 | 11841 | 13942 | 11192 | 13204 |
| *hsa-miR-100** | 0 | 0 | 0 | 0 | 1 | 6 | 3 | 7 | 1 | 3 |
| *hsa-miR-101* | 103594 | 22488 | 50238 | 41113 | 43521 | 87226 | 77161 | 85829 | 60364 | 76275 |
| *hsa-miR-101** | 3 | 0 | 0 | 0 | 3 | 4 | 5 | 10 | 1 | 7 |
| *hsa-miR-103* | 646245 | 343293 | 531097 | 589592 | 327197 | 183131 | 234574 | 180850 | 218215 | 228402 |
| *hsa-miR-103-2** | 67 | 38 | 37 | 55 | 90 | 25 | 34 | 23 | 24 | 29 |
| *hsa-miR-105* | 2 | 0 | 0 | 12 | 25 | 0 | 0 | 0 | 0 | 0 |
| *hsa-miR-105** | 0 | 0 | 0 | 21 | 32 | 0 | 0 | 0 | 0 | 0 |
| *hsa-miR-106a* | 46 | 23 | 5 | 63 | 128 | 69 | 100 | 129 | 117 | 94 |
| *hsa-miR-106a** | 1 | 0 | 1 | 3 | 8 | 4 | 1 | 1 | 1 | 4 |
| *hsa-miR-106b* | 3838 | 3100 | 5357 | 6806 | 3509 | 1231 | 1362 | 2014 | 1103 | 1653 |
| *hsa-miR-106b** | 863 | 940 | 216 | 924 | 477 | 376 | 601 | 439 | 478 | 341 |
| *hsa-miR-107* | 210147 | 122692 | 157737 | 166859 | 98353 | 96435 | 123342 | 102042 | 114238 | 100899 |
| *hsa-miR-10a* | 288208 | 4312 | 3699 | 54347 | 13731 | 7085 | 10242 | 8824 | 9597 | 11257 |
| *hsa-miR-10a** | 566 | 6 | 9 | 163 | 36 | 12 | 17 | 20 | 12 | 14 |
| *hsa-miR-10b* | 4193 | 1975 | 700 | 2244 | 4789 | 3156 | 5084 | 6851 | 4377 | 4637 |
| *hsa-miR-10b** | 15 | 3 | 0 | 5 | 14 | 6 | 11 | 14 | 10 | 6 |
| *hsa-miR-1179* | 6 | 1 | 3 | 1 | 6 | 9 | 6 | 9 | 3 | 4 |
| *hsa-miR-1180* | 3 | 14 | 1 | 0 | 0 | 22 | 15 | 3 | 9 | 23 |
| *hsa-miR-1185* | 6 | 26 | 0 | 6 | 2 | 24 | 21 | 21 | 17 | 18 |
| *hsa-miR-1193* | 5 | 10 | 0 | 0 | 1 | 6 | 4 | 3 | 2 | 4 |
| *hsa-miR-1197* | 0 | 0 | 0 | 1 | 0 | 0 | 0 | 0 | 1 | 0 |
| *hsa-miR-122* | 1225 | 2447 | 10 | 11 | 13 | 44 | 182 | 226 | 197 | 1458 |
| *hsa-miR-122** | 0 | 7 | 0 | 0 | 0 | 0 | 0 | 0 | 0 | 0 |
| *hsa-miR-1224-5p* | 10 | 9 | 4 | 13 | 12 | 10 | 21 | 7 | 13 | 15 |
| *hsa-miR-1226* | 8 | 2 | 0 | 1 | 2 | 1 | 0 | 1 | 1 | 0 |
| *hsa-miR-1226** | 2 | 0 | 1 | 2 | 1 | 0 | 0 | 0 | 0 | 0 |
| *hsa-miR-1227* | 2 | 0 | 1 | 2 | 0 | 0 | 1 | 1 | 0 | 2 |
| *hsa-miR-1228* | 4 | 3 | 1 | 3 | 0 | 0 | 1 | 0 | 1 | 0 |
| *hsa-miR-1228** | 23 | 11 | 0 | 2 | 1 | 1 | 2 | 0 | 2 | 2 |
| *hsa-miR-1229* | 7 | 2 | 1 | 1 | 9 | 1 | 1 | 2 | 1 | 0 |
| *hsa-miR-1231* | 0 | 1 | 0 | 0 | 0 | 0 | 0 | 0 | 0 | 0 |
| *hsa-miR-1234* | 3 | 2 | 0 | 2 | 1 | 0 | 0 | 1 | 0 | 0 |
| *hsa-miR-1237* | 0 | 0 | 0 | 2 | 0 | 0 | 1 | 0 | 0 | 0 |
| *hsa-miR-124* | 0 | 2 | 4 | 0 | 0 | 9 | 9 | 3 | 4 | 9 |
| *hsa-miR-1243* | 0 | 0 | 0 | 0 | 3 | 0 | 0 | 0 | 0 | 0 |
| *hsa-miR-1244* | 6 | 34 | 4 | 2 | 5 | 0 | 3 | 3 | 0 | 1 |
| *hsa-miR-1245* | 3 | 9 | 1 | 2 | 7 | 4 | 4 | 6 | 3 | 5 |
| *hsa-miR-1246* | 27 | 77 | 5 | 381 | 127 | 1 | 0 | 0 | 0 | 1 |
| *hsa-miR-1247* | 26 | 2 | 1 | 12 | 111 | 221 | 398 | 378 | 294 | 217 |
| *hsa-miR-1248* | 19 | 15 | 10 | 1 | 6 | 1 | 0 | 0 | 0 | 0 |
| *hsa-miR-1249* | 29 | 24 | 5 | 20 | 17 | 40 | 52 | 30 | 53 | 32 |
| *hsa-miR-1250* | 20 | 8 | 3 | 15 | 9 | 12 | 14 | 18 | 15 | 21 |
| *hsa-miR-1252* | 4 | 10 | 2 | 1 | 10 | 1 | 1 | 1 | 0 | 0 |
| *hsa-miR-1254* | 91 | 175 | 30 | 70 | 90 | 1 | 8 | 6 | 9 | 6 |
| *hsa-miR-1255a* | 109 | 404 | 18 | 105 | 282 | 93 | 61 | 43 | 69 | 53 |
| *hsa-miR-1255b* | 15 | 63 | 8 | 32 | 75 | 28 | 82 | 41 | 80 | 38 |
| *hsa-miR-1256* | 4 | 9 | 0 | 4 | 6 | 5 | 7 | 1 | 2 | 3 |
| *hsa-miR-1258* | 0 | 0 | 0 | 2 | 0 | 10 | 1 | 11 | 13 | 17 |
| *hsa-miR-125a-3p* | 95 | 60 | 90 | 292 | 151 | 36 | 32 | 44 | 51 | 69 |
| *hsa-miR-125a-5p* | 16058 | 2731 | 4702 | 8945 | 2791 | 6104 | 8700 | 8042 | 9151 | 8902 |
| *hsa-miR-125b* | 1943 | 3335 | 87 | 1879 | 4159 | 25408 | 37437 | 38951 | 35716 | 32090 |
| *hsa-miR-125b-1** | 3 | 0 | 1 | 2 | 15 | 37 | 26 | 18 | 12 | 31 |
| *hsa-miR-125b-2** | 4 | 16 | 1 | 29 | 30 | 189 | 158 | 195 | 119 | 125 |
| *hsa-miR-126* | 167 | 30 | 35 | 144 | 88 | 86 | 136 | 158 | 109 | 127 |
| *hsa-miR-126** | 601 | 153 | 165 | 345 | 276 | 369 | 547 | 871 | 490 | 521 |
| *hsa-miR-1260* | 1 | 0 | 3 | 0 | 0 | 1 | 0 | 0 | 0 | 0 |
| *hsa-miR-1260b* | 7 | 11 | 0 | 4 | 31 | 5 | 2 | 6 | 5 | 9 |
| *hsa-miR-1262* | 36 | 13 | 5 | 8 | 68 | 60 | 95 | 99 | 95 | 131 |
| *hsa-miR-1265* | 0 | 0 | 0 | 0 | 0 | 4 | 14 | 10 | 10 | 1 |
| *hsa-miR-1266* | 69 | 55 | 18 | 28 | 48 | 9 | 18 | 9 | 4 | 4 |
| *hsa-miR-1267* | 0 | 0 | 0 | 0 | 1 | 0 | 0 | 0 | 0 | 0 |
| *hsa-miR-1268* | 572 | 35 | 75 | 716 | 474 | 14 | 21 | 13 | 5 | 12 |
| *hsa-miR-1269* | 1041 | 7 | 1600 | 346 | 12464 | 105 | 27 | 37 | 19 | 58 |
| *hsa-miR-1270* | 161 | 39 | 39 | 121 | 8 | 30 | 52 | 32 | 52 | 56 |
| *hsa-miR-1271* | 32 | 128 | 27 | 164 | 85 | 104 | 118 | 113 | 94 | 115 |
| *hsa-miR-1272* | 0 | 2 | 0 | 2 | 0 | 0 | 0 | 0 | 0 | 1 |
| *hsa-miR-1273* | 1 | 0 | 0 | 0 | 2 | 0 | 0 | 0 | 0 | 0 |
| *hsa-miR-1273c* | 10 | 14 | 1 | 5 | 10 | 4 | 12 | 6 | 8 | 3 |
| *hsa-miR-1273d* | 6 | 0 | 3 | 6 | 2 | 0 | 0 | 0 | 0 | 0 |
| *hsa-miR-1273e* | 0 | 2 | 2 | 0 | 2 | 0 | 0 | 0 | 0 | 0 |
| *hsa-miR-127-3p* | 1242 | 753 | 93 | 693 | 666 | 1599 | 2812 | 2045 | 1998 | 2014 |
| *hsa-miR-1274a* | 0 | 0 | 0 | 0 | 1 | 0 | 0 | 0 | 0 | 0 |
| *hsa-miR-1274b* | 6 | 6 | 26 | 9 | 46 | 11 | 3 | 3 | 2 | 8 |
| *hsa-miR-1275* | 9 | 1 | 12 | 12 | 34 | 6 | 7 | 3 | 2 | 3 |
| *hsa-miR-127-5p* | 20 | 10 | 5 | 28 | 24 | 42 | 54 | 33 | 30 | 59 |
| *hsa-miR-1276* | 22 | 4 | 2 | 20 | 2 | 3 | 0 | 2 | 0 | 0 |
| *hsa-miR-1277* | 270 | 279 | 87 | 30 | 94 | 176 | 93 | 115 | 69 | 95 |
| *hsa-miR-1278* | 98 | 278 | 41 | 70 | 243 | 57 | 49 | 67 | 51 | 60 |
| *hsa-miR-128* | 9714 | 6400 | 4848 | 6079 | 3106 | 3330 | 3461 | 3954 | 3973 | 4188 |
| *hsa-miR-1283* | 136 | 2 | 0 | 931 | 108 | 0 | 0 | 0 | 0 | 0 |
| *hsa-miR-1284* | 29 | 13 | 8 | 24 | 46 | 9 | 8 | 10 | 11 | 6 |
| *hsa-miR-1285* | 95 | 226 | 61 | 120 | 114 | 26 | 39 | 59 | 45 | 42 |
| *hsa-miR-1286* | 4 | 1 | 1 | 4 | 0 | 0 | 2 | 0 | 0 | 1 |
| *hsa-miR-1287* | 224 | 26 | 134 | 458 | 55 | 68 | 93 | 61 | 78 | 98 |
| *hsa-miR-1288* | 2 | 0 | 1 | 2 | 0 | 0 | 0 | 0 | 1 | 1 |
| *hsa-miR-1289* | 19 | 14 | 8 | 2 | 1 | 2 | 4 | 3 | 0 | 0 |
| *hsa-miR-129** | 0 | 5 | 0 | 5 | 1 | 16 | 21 | 36 | 24 | 28 |
| *hsa-miR-1291* | 195 | 202 | 99 | 119 | 229 | 138 | 181 | 185 | 190 | 151 |
| *hsa-miR-1292* | 75 | 128 | 34 | 84 | 69 | 8 | 3 | 7 | 6 | 4 |
| *hsa-miR-1293* | 13 | 92 | 6 | 5 | 17 | 0 | 0 | 0 | 0 | 0 |
| *hsa-miR-129-3p* | 105 | 76 | 3 | 99 | 66 | 116 | 146 | 280 | 182 | 236 |
| *hsa-miR-1294* | 14 | 19 | 4 | 5 | 4 | 26 | 46 | 38 | 52 | 29 |
| *hsa-miR-1295* | 4 | 1 | 7 | 2 | 4 | 0 | 1 | 0 | 1 | 1 |
| *hsa-miR-129-5p* | 9 | 10 | 0 | 1 | 3 | 5 | 5 | 11 | 4 | 7 |
| *hsa-miR-1296* | 12 | 1 | 9 | 30 | 18 | 48 | 18 | 28 | 16 | 29 |
| *hsa-miR-1298* | 5 | 0 | 0 | 0 | 7 | 18 | 17 | 21 | 16 | 22 |
| *hsa-miR-1299* | 1 | 36 | 291 | 66 | 13 | 46 | 105 | 247 | 91 | 294 |
| *hsa-miR-1301* | 350 | 311 | 358 | 1137 | 583 | 125 | 147 | 115 | 134 | 124 |
| *hsa-miR-1302* | 1 | 2 | 5 | 0 | 0 | 1 | 0 | 1 | 1 | 1 |
| *hsa-miR-1303* | 7 | 19 | 7 | 24 | 39 | 8 | 0 | 3 | 3 | 5 |
| *hsa-miR-1304* | 8 | 23 | 1 | 10 | 7 | 3 | 5 | 6 | 7 | 5 |
| *hsa-miR-1306* | 94 | 49 | 26 | 64 | 28 | 32 | 41 | 16 | 39 | 40 |
| *hsa-miR-1307* | 11597 | 5435 | 4603 | 16262 | 9046 | 594 | 632 | 636 | 717 | 1261 |
| *hsa-miR-130a* | 1463 | 1489 | 173 | 1500 | 1613 | 3539 | 4154 | 4482 | 3877 | 3871 |
| *hsa-miR-130a** | 0 | 2 | 1 | 1 | 7 | 1 | 9 | 3 | 2 | 1 |
| *hsa-miR-130b* | 1794 | 1359 | 427 | 915 | 837 | 133 | 112 | 202 | 144 | 167 |
| *hsa-miR-130b** | 20 | 24 | 9 | 14 | 12 | 3 | 5 | 3 | 0 | 5 |
| *hsa-miR-132* | 36 | 75 | 30 | 133 | 207 | 66 | 160 | 276 | 146 | 203 |
| *hsa-miR-132** | 27 | 30 | 10 | 31 | 47 | 44 | 138 | 118 | 128 | 128 |
| *hsa-miR-1321* | 0 | 0 | 1 | 0 | 0 | 0 | 0 | 0 | 0 | 0 |
| *hsa-miR-1323* | 14159 | 59 | 1081 | 55798 | 601 | 9 | 11 | 11 | 8 | 15 |
| *hsa-miR-133a* | 88 | 25 | 14 | 80 | 134 | 5513 | 6472 | 6261 | 5164 | 7087 |
| *hsa-miR-133b* | 6 | 2 | 0 | 7 | 10 | 661 | 647 | 701 | 517 | 740 |
| *hsa-miR-134* | 78 | 358 | 22 | 65 | 108 | 81 | 144 | 211 | 107 | 173 |
| *hsa-miR-135a* | 25 | 0 | 1 | 9 | 9 | 11 | 4 | 11 | 3 | 8 |
| *hsa-miR-135a** | 2 | 0 | 0 | 0 | 0 | 0 | 0 | 0 | 0 | 1 |
| *hsa-miR-135b* | 133 | 283 | 34 | 471 | 527 | 4 | 5 | 3 | 1 | 3 |
| *hsa-miR-135b** | 5 | 3 | 2 | 1 | 3 | 0 | 0 | 0 | 0 | 0 |
| *hsa-miR-136* | 103 | 54 | 3 | 9 | 29 | 120 | 151 | 163 | 130 | 109 |
| *hsa-miR-136** | 18 | 14 | 6 | 21 | 22 | 46 | 59 | 76 | 38 | 50 |
| *hsa-miR-137* | 0 | 0 | 0 | 0 | 0 | 2 | 1 | 1 | 2 | 4 |
| *hsa-miR-138* | 388 | 83 | 13 | 1351 | 133 | 20 | 22 | 11 | 17 | 16 |
| *hsa-miR-138-1** | 3 | 3 | 0 | 15 | 6 | 1 | 0 | 0 | 0 | 1 |
| *hsa-miR-139-3p* | 402 | 87 | 35 | 260 | 221 | 1372 | 1875 | 1341 | 1967 | 1653 |
| *hsa-miR-139-5p* | 217 | 90 | 27 | 254 | 265 | 2797 | 3948 | 2540 | 3387 | 3041 |
| *hsa-miR-140-3p* | 181014 | 77206 | 30852 | 136896 | 67241 | 213612 | 259892 | 237348 | 238076 | 287930 |
| *hsa-miR-140-5p* | 5 | 7 | 10 | 14 | 9 | 16 | 23 | 29 | 10 | 19 |
| *hsa-miR-141* | 1313 | 496 | 1295 | 618 | 1440 | 37 | 23 | 56 | 11 | 23 |
| *hsa-miR-141** | 21 | 28 | 16 | 5 | 60 | 0 | 0 | 1 | 0 | 1 |
| *hsa-miR-142-3p* | 121 | 133 | 13 | 204 | 638 | 142 | 234 | 403 | 172 | 263 |
| *hsa-miR-142-5p* | 3225 | 2579 | 223 | 4994 | 7965 | 4367 | 6114 | 7984 | 4807 | 6400 |
| *hsa-miR-143* | 56506 | 10301 | 7284 | 29304 | 15371 | 378086 | 366485 | 402356 | 286728 | 524554 |
| *hsa-miR-143** | 2255 | 138 | 475 | 978 | 698 | 5375 | 6899 | 6477 | 1696 | 8731 |
| *hsa-miR-144* | 146 | 66 | 48 | 78 | 28 | 753 | 466 | 677 | 333 | 545 |
| *hsa-miR-144** | 448 | 249 | 186 | 215 | 118 | 2592 | 2898 | 2436 | 2447 | 1753 |
| *hsa-miR-145* | 8223 | 3676 | 1209 | 9845 | 5005 | 219925 | 218864 | 235700 | 185159 | 279606 |
| *hsa-miR-145** | 246 | 93 | 37 | 85 | 98 | 2034 | 1861 | 2173 | 1783 | 2353 |
| *hsa-miR-1468* | 6 | 0 | 6 | 7 | 15 | 5 | 5 | 7 | 8 | 8 |
| *hsa-miR-146a* | 2920 | 368 | 41 | 2816 | 2799 | 274 | 397 | 541 | 318 | 364 |
| *hsa-miR-146a** | 7 | 1 | 0 | 0 | 0 | 0 | 1 | 2 | 0 | 0 |
| *hsa-miR-146b-3p* | 7 | 50 | 2 | 14 | 22 | 12 | 50 | 109 | 35 | 77 |
| *hsa-miR-146b-5p* | 5669 | 37226 | 2982 | 17921 | 48034 | 2690 | 10595 | 15025 | 9940 | 9988 |
| *hsa-miR-147b* | 7 | 18 | 1 | 4 | 14 | 1 | 1 | 0 | 0 | 0 |
| *hsa-miR-148a* | 9151 | 4714 | 420 | 2985 | 11528 | 3444 | 3840 | 4469 | 3089 | 3302 |
| *hsa-miR-148a** | 34 | 5 | 1 | 14 | 37 | 5 | 8 | 10 | 6 | 7 |
| *hsa-miR-148b* | 4325 | 2372 | 4867 | 4001 | 3330 | 1600 | 1467 | 1754 | 1357 | 1480 |
| *hsa-miR-148b** | 150 | 36 | 23 | 32 | 22 | 14 | 16 | 15 | 6 | 22 |
| *hsa-miR-149* | 118 | 19 | 354 | 373 | 163 | 13 | 23 | 16 | 13 | 18 |
| *hsa-miR-149** | 25 | 1 | 49 | 36 | 20 | 6 | 5 | 0 | 2 | 3 |
| *hsa-miR-150* | 402 | 123 | 22 | 1546 | 795 | 515 | 644 | 783 | 689 | 619 |
| *hsa-miR-150** | 64 | 9 | 3 | 68 | 41 | 29 | 54 | 29 | 45 | 38 |
| *hsa-miR-151-3p* | 10650 | 3528 | 3638 | 9925 | 1979 | 734 | 985 | 1019 | 757 | 864 |
| *hsa-miR-151-5p* | 3964 | 1993 | 1480 | 2683 | 759 | 851 | 790 | 954 | 835 | 690 |
| *hsa-miR-152* | 5978 | 7798 | 1025 | 2048 | 4635 | 11651 | 15875 | 12245 | 12689 | 12085 |
| *hsa-miR-153* | 4 | 1 | 0 | 1 | 0 | 2 | 4 | 2 | 1 | 2 |
| *hsa-miR-1537* | 9 | 8 | 5 | 7 | 6 | 3 | 9 | 12 | 5 | 11 |
| *hsa-miR-154* | 61 | 104 | 10 | 16 | 39 | 81 | 142 | 181 | 167 | 125 |
| *hsa-miR-154** | 8 | 140 | 1 | 5 | 10 | 15 | 23 | 26 | 9 | 10 |
| *hsa-miR-155* | 623 | 1157 | 61 | 2493 | 2211 | 142 | 276 | 368 | 234 | 317 |
| *hsa-miR-155** | 0 | 2 | 1 | 2 | 7 | 3 | 2 | 4 | 1 | 5 |
| *hsa-miR-15a* | 3618 | 3722 | 3793 | 3102 | 1861 | 1851 | 2343 | 2890 | 2272 | 2363 |
| *hsa-miR-15a** | 3 | 1 | 16 | 14 | 5 | 1 | 3 | 1 | 1 | 3 |
| *hsa-miR-15b* | 1915 | 2497 | 3876 | 8056 | 2891 | 3425 | 4967 | 5882 | 4347 | 4365 |
| *hsa-miR-15b** | 145 | 185 | 83 | 100 | 41 | 31 | 39 | 57 | 23 | 41 |
| *hsa-miR-16* | 10550 | 13801 | 15615 | 19236 | 13588 | 15190 | 23278 | 21807 | 22793 | 17736 |
| *hsa-miR-16-1** | 31 | 8 | 10 | 12 | 6 | 2 | 3 | 5 | 0 | 5 |
| *hsa-miR-16-2** | 70 | 117 | 56 | 78 | 61 | 26 | 79 | 40 | 59 | 27 |
| *hsa-miR-17* | 1048 | 2163 | 491 | 1205 | 2001 | 446 | 797 | 756 | 580 | 621 |
| *hsa-miR-17** | 666 | 1519 | 183 | 361 | 456 | 193 | 187 | 281 | 210 | 257 |
| *hsa-miR-181a* | 28837 | 17210 | 3443 | 15284 | 13569 | 3969 | 4877 | 6072 | 4524 | 6856 |
| *hsa-miR-181a** | 266 | 150 | 40 | 281 | 215 | 47 | 100 | 135 | 71 | 109 |
| *hsa-miR-181a-2** | 940 | 569 | 153 | 328 | 374 | 219 | 212 | 314 | 232 | 231 |
| *hsa-miR-181b* | 23161 | 14937 | 2729 | 5880 | 8044 | 2178 | 2502 | 3235 | 2510 | 3575 |
| *hsa-miR-181c* | 629 | 444 | 804 | 645 | 567 | 347 | 280 | 265 | 193 | 290 |
| *hsa-miR-181c** | 101 | 34 | 316 | 213 | 91 | 52 | 42 | 42 | 33 | 39 |
| *hsa-miR-181d* | 4051 | 3447 | 6949 | 4003 | 3514 | 1865 | 1729 | 1592 | 1853 | 2238 |
| *hsa-miR-182* | 2121 | 588 | 1896 | 1253 | 244 | 95 | 112 | 95 | 87 | 38 |
| *hsa-miR-182** | 3 | 3 | 6 | 4 | 0 | 0 | 0 | 0 | 0 | 0 |
| *hsa-miR-183* | 981 | 186 | 924 | 571 | 98 | 33 | 54 | 45 | 32 | 25 |
| *hsa-miR-183** | 129 | 46 | 271 | 303 | 42 | 4 | 2 | 1 | 2 | 3 |
| *hsa-miR-184* | 429 | 92 | 56 | 201 | 56 | 67 | 148 | 181 | 122 | 67 |
| *hsa-miR-185* | 32975 | 40963 | 15007 | 51076 | 16434 | 33013 | 39062 | 32020 | 41642 | 29437 |
| *hsa-miR-185** | 48 | 5 | 28 | 25 | 29 | 28 | 42 | 21 | 6 | 22 |
| *hsa-miR-186* | 2164 | 1093 | 1301 | 2834 | 804 | 1533 | 2122 | 1944 | 1624 | 1687 |
| *hsa-miR-186** | 114 | 135 | 228 | 39 | 39 | 6 | 15 | 20 | 9 | 27 |
| *hsa-miR-187* | 877 | 200 | 201 | 610 | 271 | 391 | 312 | 563 | 265 | 627 |
| *hsa-miR-187** | 23 | 5 | 3 | 24 | 5 | 4 | 8 | 9 | 6 | 10 |
| *hsa-miR-188-3p* | 0 | 0 | 0 | 0 | 0 | 4 | 0 | 0 | 0 | 1 |
| *hsa-miR-188-5p* | 9 | 0 | 7 | 0 | 6 | 10 | 4 | 4 | 0 | 5 |
| *hsa-miR-18a* | 87 | 58 | 24 | 37 | 98 | 30 | 39 | 37 | 14 | 47 |
| *hsa-miR-18a** | 10 | 19 | 6 | 26 | 44 | 9 | 22 | 9 | 8 | 8 |
| *hsa-miR-18b* | 4 | 0 | 3 | 3 | 4 | 4 | 6 | 5 | 5 | 3 |
| *hsa-miR-18b** | 0 | 0 | 0 | 0 | 2 | 0 | 7 | 1 | 2 | 3 |
| *hsa-miR-190* | 2 | 8 | 6 | 1 | 0 | 15 | 3 | 12 | 8 | 4 |
| *hsa-miR-1908* | 9 | 8 | 0 | 0 | 0 | 5 | 1 | 4 | 6 | 5 |
| *hsa-miR-1909** | 0 | 1 | 0 | 0 | 0 | 0 | 0 | 0 | 0 | 0 |
| *hsa-miR-190b* | 7 | 9 | 5 | 7 | 10 | 4 | 7 | 8 | 10 | 12 |
| *hsa-miR-191* | 197801 | 103623 | 146647 | 307039 | 330304 | 48359 | 53461 | 71927 | 48303 | 61866 |
| *hsa-miR-191** | 74 | 2 | 46 | 53 | 43 | 4 | 9 | 8 | 5 | 14 |
| *hsa-miR-1910* | 0 | 1 | 0 | 1 | 2 | 0 | 0 | 0 | 0 | 0 |
| *hsa-miR-1911* | 0 | 0 | 8 | 1 | 14 | 0 | 0 | 0 | 0 | 0 |
| *hsa-miR-1912* | 0 | 0 | 0 | 0 | 0 | 2 | 0 | 2 | 0 | 0 |
| *hsa-miR-1914* | 4 | 0 | 0 | 0 | 0 | 0 | 0 | 0 | 1 | 0 |
| *hsa-miR-1914** | 5 | 0 | 0 | 5 | 0 | 0 | 0 | 0 | 0 | 0 |
| *hsa-miR-1915* | 1 | 2 | 0 | 0 | 4 | 0 | 0 | 0 | 0 | 0 |
| *hsa-miR-1915** | 2 | 1 | 0 | 0 | 7 | 0 | 0 | 0 | 0 | 0 |
| *hsa-miR-192* | 17510 | 7490 | 6530 | 31685 | 23986 | 7530 | 9005 | 10250 | 9519 | 9008 |
| *hsa-miR-192** | 0 | 0 | 0 | 7 | 10 | 0 | 1 | 0 | 2 | 2 |
| *hsa-miR-193a-3p* | 546 | 1161 | 678 | 4613 | 11341 | 1826 | 1523 | 3523 | 1118 | 2915 |
| *hsa-miR-193a-5p* | 396 | 1695 | 102 | 776 | 1266 | 940 | 655 | 830 | 1171 | 1141 |
| *hsa-miR-193b* | 3029 | 4253 | 2001 | 2548 | 3850 | 2584 | 2491 | 2523 | 2326 | 2699 |
| *hsa-miR-193b** | 896 | 1922 | 839 | 1130 | 887 | 1330 | 1564 | 967 | 1655 | 1494 |
| *hsa-miR-194* | 65 | 64 | 41 | 199 | 352 | 42 | 100 | 81 | 70 | 74 |
| *hsa-miR-194** | 0 | 0 | 0 | 3 | 3 | 0 | 0 | 0 | 1 | 0 |
| *hsa-miR-195* | 481 | 141 | 89 | 600 | 490 | 2560 | 2041 | 3663 | 2120 | 3242 |
| *hsa-miR-195** | 2 | 0 | 0 | 1 | 0 | 1 | 6 | 1 | 2 | 3 |
| *hsa-miR-196a* | 16 | 542 | 4 | 53 | 72 | 75 | 134 | 155 | 162 | 147 |
| *hsa-miR-196b* | 416 | 165 | 39 | 267 | 1447 | 370 | 388 | 658 | 423 | 460 |
| *hsa-miR-196b** | 36 | 9 | 3 | 18 | 42 | 37 | 41 | 53 | 28 | 36 |
| *hsa-miR-197* | 413 | 478 | 262 | 691 | 1196 | 403 | 494 | 583 | 509 | 630 |
| *hsa-miR-1972* | 0 | 0 | 2 | 0 | 0 | 0 | 0 | 0 | 0 | 0 |
| *hsa-miR-1976* | 0 | 0 | 3 | 5 | 1 | 0 | 3 | 4 | 0 | 1 |
| *hsa-miR-199a-3p* | 107708 | 110520 | 6465 | 37503 | 71181 | 225914 | 274491 | 262783 | 228056 | 254245 |
| *hsa-miR-199a-5p* | 342 | 461 | 39 | 421 | 389 | 802 | 1125 | 1452 | 931 | 987 |
| *hsa-miR-199b-3p* | 107705 | 110520 | 6465 | 37499 | 71177 | 225910 | 274481 | 262772 | 228046 | 254241 |
| *hsa-miR-199b-5p* | 424 | 353 | 69 | 438 | 674 | 1565 | 1620 | 2770 | 1762 | 1794 |
| *hsa-miR-19a* | 13 | 36 | 6 | 17 | 26 | 5 | 19 | 17 | 11 | 11 |
| *hsa-miR-19a** | 4 | 1 | 0 | 2 | 1 | 1 | 1 | 4 | 0 | 1 |
| *hsa-miR-19b* | 565 | 1091 | 694 | 1952 | 2551 | 454 | 554 | 1009 | 428 | 644 |
| *hsa-miR-19b-1** | 0 | 2 | 1 | 1 | 5 | 1 | 0 | 0 | 0 | 1 |
| *hsa-miR-19b-2** | 0 | 0 | 0 | 0 | 1 | 0 | 0 | 0 | 0 | 0 |
| *hsa-miR-200a* | 20759 | 1831 | 31731 | 12467 | 6784 | 634 | 137 | 291 | 121 | 223 |
| *hsa-miR-200a** | 56 | 13 | 417 | 119 | 154 | 0 | 1 | 4 | 1 | 1 |
| *hsa-miR-200b* | 10171 | 831 | 17475 | 11237 | 5026 | 375 | 130 | 232 | 116 | 186 |
| *hsa-miR-200b** | 1687 | 443 | 6646 | 4742 | 3812 | 182 | 60 | 157 | 51 | 86 |
| *hsa-miR-200c* | 40881 | 12323 | 34671 | 48462 | 76255 | 1150 | 443 | 911 | 499 | 732 |
| *hsa-miR-200c** | 12 | 8 | 23 | 11 | 66 | 2 | 0 | 1 | 0 | 0 |
| *hsa-miR-202* | 0 | 1 | 0 | 4 | 1 | 10 | 10 | 19 | 3 | 16 |
| *hsa-miR-202** | 1 | 17 | 3 | 31 | 61 | 141 | 51 | 405 | 59 | 197 |
| *hsa-miR-203* | 3567 | 4565 | 801 | 6870 | 71611 | 239 | 251 | 244 | 212 | 238 |
| *hsa-miR-204* | 11 | 1 | 1 | 49 | 23 | 265 | 822 | 879 | 723 | 750 |
| *hsa-miR-205* | 51953 | 26407 | 36197 | 41796 | 35256 | 2360 | 1151 | 1747 | 912 | 1123 |
| *hsa-miR-205** | 36 | 35 | 38 | 11 | 24 | 2 | 2 | 1 | 0 | 1 |
| *hsa-miR-206* | 30 | 22 | 2 | 9 | 7 | 97 | 423 | 833 | 371 | 1486 |
| *hsa-miR-208a* | 0 | 0 | 0 | 0 | 0 | 1 | 0 | 0 | 0 | 0 |
| *hsa-miR-208b* | 0 | 0 | 1 | 1 | 0 | 0 | 0 | 0 | 0 | 0 |
| *hsa-miR-20a* | 367 | 717 | 171 | 365 | 563 | 140 | 319 | 332 | 238 | 210 |
| *hsa-miR-20a** | 5 | 3 | 6 | 5 | 7 | 4 | 5 | 7 | 3 | 3 |
| *hsa-miR-20b* | 16 | 2 | 3 | 27 | 40 | 19 | 42 | 32 | 40 | 38 |
| *hsa-miR-20b** | 40 | 4 | 2 | 22 | 37 | 9 | 18 | 17 | 16 | 16 |
| *hsa-miR-21* | 478660 | 619533 | 116727 | 271059 | 584836 | 49020 | 42321 | 53481 | 34761 | 52051 |
| *hsa-miR-21** | 495 | 575 | 106 | 274 | 717 | 102 | 188 | 224 | 139 | 308 |
| *hsa-miR-210* | 4768 | 5938 | 2111 | 1576 | 1310 | 110 | 100 | 110 | 127 | 153 |
| *hsa-miR-211* | 0 | 0 | 0 | 1 | 1 | 0 | 0 | 0 | 0 | 0 |
| *hsa-miR-2110* | 547 | 280 | 320 | 957 | 470 | 210 | 209 | 182 | 236 | 216 |
| *hsa-miR-2114* | 1 | 1 | 0 | 1 | 5 | 7 | 9 | 16 | 5 | 5 |
| *hsa-miR-2114** | 0 | 0 | 0 | 1 | 4 | 2 | 4 | 5 | 5 | 4 |
| *hsa-miR-2115* | 0 | 0 | 0 | 1 | 0 | 0 | 3 | 0 | 0 | 1 |
| *hsa-miR-2115** | 0 | 1 | 0 | 1 | 0 | 0 | 5 | 5 | 1 | 5 |
| *hsa-miR-2116* | 0 | 3 | 0 | 0 | 1 | 0 | 0 | 1 | 0 | 0 |
| *hsa-miR-2116** | 5 | 8 | 4 | 111 | 2 | 3 | 3 | 2 | 6 | 3 |
| *hsa-miR-2117* | 0 | 1 | 0 | 0 | 0 | 0 | 0 | 0 | 0 | 0 |
| *hsa-miR-212* | 18 | 38 | 7 | 34 | 65 | 47 | 77 | 186 | 84 | 170 |
| *hsa-miR-214* | 195 | 331 | 21 | 281 | 293 | 505 | 758 | 993 | 577 | 863 |
| *hsa-miR-214** | 6 | 7 | 1 | 6 | 20 | 16 | 21 | 27 | 17 | 20 |
| *hsa-miR-215* | 224 | 47 | 24 | 168 | 51 | 17 | 16 | 15 | 10 | 16 |
| *hsa-miR-216a* | 0 | 1 | 0 | 0 | 0 | 0 | 0 | 0 | 0 | 0 |
| *hsa-miR-216b* | 4 | 24 | 0 | 0 | 9 | 0 | 2 | 0 | 0 | 0 |
| *hsa-miR-217* | 15 | 57 | 0 | 1 | 10 | 1 | 3 | 11 | 2 | 8 |
| *hsa-miR-218* | 27 | 0 | 22 | 14 | 3 | 26 | 37 | 61 | 40 | 51 |
| *hsa-miR-218-1** | 0 | 0 | 0 | 3 | 2 | 29 | 22 | 42 | 20 | 28 |
| *hsa-miR-218-2** | 8 | 0 | 4 | 1 | 0 | 0 | 1 | 2 | 0 | 2 |
| *hsa-miR-219-1-3p* | 8 | 6 | 12 | 32 | 50 | 10 | 10 | 6 | 6 | 6 |
| *hsa-miR-219-5p* | 13 | 3 | 40 | 31 | 88 | 7 | 11 | 13 | 4 | 10 |
| *hsa-miR-22* | 8063 | 5345 | 3575 | 9266 | 12754 | 8902 | 9238 | 12359 | 8213 | 9494 |
| *hsa-miR-22** | 504 | 681 | 293 | 523 | 742 | 313 | 325 | 520 | 233 | 516 |
| *hsa-miR-221* | 32084 | 24476 | 1162 | 13484 | 36641 | 54587 | 55621 | 40196 | 44417 | 48187 |
| *hsa-miR-221** | 2775 | 5507 | 150 | 1173 | 4757 | 9481 | 7200 | 7507 | 7288 | 8643 |
| *hsa-miR-222* | 15317 | 21302 | 1576 | 25251 | 32264 | 39481 | 32548 | 29514 | 29375 | 32003 |
| *hsa-miR-222** | 2 | 0 | 0 | 1 | 4 | 7 | 6 | 3 | 7 | 11 |
| *hsa-miR-223* | 402 | 1725 | 33 | 2018 | 2589 | 2711 | 7160 | 11403 | 6519 | 10171 |
| *hsa-miR-223** | 18 | 105 | 1 | 50 | 54 | 64 | 183 | 191 | 186 | 313 |
| *hsa-miR-224* | 3271 | 3751 | 1191 | 309 | 4694 | 99 | 143 | 91 | 135 | 76 |
| *hsa-miR-224** | 97 | 47 | 167 | 49 | 674 | 11 | 21 | 22 | 15 | 17 |
| *hsa-miR-2276* | 2 | 1 | 0 | 10 | 3 | 0 | 0 | 0 | 0 | 1 |
| *hsa-miR-2277-3p* | 0 | 0 | 4 | 9 | 2 | 1 | 0 | 2 | 0 | 2 |
| *hsa-miR-2277-5p* | 2 | 10 | 0 | 0 | 4 | 0 | 0 | 0 | 1 | 0 |
| *hsa-miR-2278* | 0 | 2 | 1 | 4 | 1 | 1 | 1 | 0 | 0 | 0 |
| *hsa-miR-2355-3p* | 20 | 36 | 6 | 13 | 37 | 11 | 16 | 19 | 18 | 16 |
| *hsa-miR-2355-5p* | 22 | 71 | 11 | 89 | 118 | 86 | 71 | 74 | 68 | 93 |
| *hsa-miR-23a* | 31767 | 84067 | 26146 | 62746 | 53765 | 60191 | 54548 | 65961 | 49200 | 54820 |
| *hsa-miR-23a** | 20 | 222 | 16 | 34 | 24 | 62 | 71 | 52 | 51 | 33 |
| *hsa-miR-23b* | 15960 | 5690 | 3354 | 24924 | 16680 | 69649 | 61956 | 60368 | 56879 | 54318 |
| *hsa-miR-23b** | 2157 | 1088 | 269 | 1038 | 893 | 3131 | 3347 | 2713 | 3497 | 3418 |
| *hsa-miR-23c* | 0 | 0 | 0 | 1 | 1 | 1 | 1 | 4 | 4 | 3 |
| *hsa-miR-24* | 18653 | 24230 | 6752 | 15141 | 11854 | 14654 | 13478 | 16728 | 11542 | 14957 |
| *hsa-miR-24-1** | 18 | 7 | 4 | 7 | 32 | 48 | 30 | 62 | 37 | 39 |
| *hsa-miR-24-2** | 103 | 192 | 60 | 116 | 122 | 91 | 90 | 99 | 84 | 74 |
| *hsa-miR-25* | 37015 | 43869 | 18636 | 41005 | 29442 | 20056 | 41970 | 32365 | 38148 | 27565 |
| *hsa-miR-25** | 1369 | 1453 | 885 | 2525 | 637 | 285 | 352 | 333 | 324 | 344 |
| *hsa-miR-26a* | 32920 | 17423 | 12478 | 21871 | 26402 | 28872 | 31590 | 42102 | 31697 | 40126 |
| *hsa-miR-26a-1** | 4 | 2 | 1 | 6 | 9 | 7 | 2 | 4 | 0 | 2 |
| *hsa-miR-26a-2** | 5 | 5 | 4 | 0 | 9 | 5 | 6 | 6 | 7 | 9 |
| *hsa-miR-26b* | 40190 | 30950 | 44176 | 26225 | 35545 | 36844 | 33844 | 49914 | 33008 | 43563 |
| *hsa-miR-26b** | 1 | 1 | 13 | 4 | 13 | 8 | 6 | 2 | 12 | 4 |
| *hsa-miR-27a* | 8845 | 1862 | 6986 | 12514 | 8435 | 9992 | 6366 | 7623 | 4139 | 7657 |
| *hsa-miR-27a** | 21 | 169 | 6 | 8 | 16 | 41 | 47 | 30 | 45 | 45 |
| *hsa-miR-27b* | 13540 | 2801 | 5772 | 13840 | 9077 | 14536 | 11410 | 11790 | 8245 | 11885 |
| *hsa-miR-27b** | 495 | 169 | 112 | 439 | 193 | 452 | 562 | 530 | 533 | 620 |
| *hsa-miR-28-3p* | 1763 | 894 | 870 | 1336 | 818 | 2211 | 2801 | 2054 | 2493 | 2861 |
| *hsa-miR-28-5p* | 524 | 162 | 297 | 349 | 256 | 718 | 787 | 771 | 738 | 836 |
| *hsa-miR-296-3p* | 1 | 0 | 7 | 148 | 5 | 1 | 5 | 3 | 1 | 3 |
| *hsa-miR-296-5p* | 19 | 1 | 12 | 83 | 16 | 71 | 60 | 29 | 39 | 58 |
| *hsa-miR-299-3p* | 8 | 17 | 2 | 5 | 4 | 12 | 25 | 13 | 21 | 14 |
| *hsa-miR-299-5p* | 53 | 66 | 9 | 87 | 124 | 222 | 195 | 365 | 257 | 268 |
| *hsa-miR-29a* | 111873 | 163120 | 14950 | 65009 | 85293 | 181853 | 207090 | 189257 | 177869 | 181224 |
| *hsa-miR-29a** | 89 | 162 | 12 | 18 | 65 | 71 | 79 | 78 | 59 | 64 |
| *hsa-miR-29b* | 19071 | 4705 | 7553 | 8456 | 9550 | 5393 | 5549 | 5952 | 4323 | 5363 |
| *hsa-miR-29b-1** | 21 | 30 | 5 | 7 | 5 | 3 | 7 | 10 | 5 | 24 |
| *hsa-miR-29b-2** | 257 | 20 | 135 | 128 | 189 | 45 | 59 | 52 | 55 | 62 |
| *hsa-miR-29c* | 87026 | 4652 | 44534 | 68729 | 52226 | 36789 | 32953 | 41942 | 28014 | 34884 |
| *hsa-miR-29c** | 1103 | 53 | 842 | 1195 | 669 | 343 | 430 | 459 | 346 | 371 |
| *hsa-miR-300* | 0 | 0 | 0 | 0 | 0 | 0 | 1 | 0 | 0 | 0 |
| *hsa-miR-301a* | 71 | 22 | 47 | 37 | 24 | 15 | 11 | 15 | 6 | 30 |
| *hsa-miR-301b* | 92 | 15 | 22 | 22 | 16 | 0 | 0 | 1 | 0 | 1 |
| *hsa-miR-302b* | 0 | 0 | 0 | 0 | 0 | 1 | 0 | 0 | 0 | 0 |
| *hsa-miR-302d* | 0 | 1 | 0 | 0 | 0 | 0 | 0 | 0 | 0 | 0 |
| *hsa-miR-3065-3p* | 4 | 7 | 4 | 10 | 334 | 1 | 4 | 4 | 0 | 3 |
| *hsa-miR-3065-5p* | 8 | 61 | 4 | 8 | 561 | 2 | 3 | 7 | 5 | 3 |
| *hsa-miR-3074* | 0 | 0 | 0 | 2 | 0 | 0 | 0 | 0 | 0 | 1 |
| *hsa-miR-30a* | 14543 | 19973 | 1615 | 7864 | 4167 | 32867 | 41802 | 47485 | 36157 | 35970 |
| *hsa-miR-30a** | 1225 | 1100 | 148 | 679 | 431 | 3866 | 4592 | 4820 | 3715 | 3873 |
| *hsa-miR-30b* | 1603 | 1010 | 2638 | 2810 | 3054 | 1815 | 2140 | 3081 | 1776 | 2496 |
| *hsa-miR-30b** | 214 | 261 | 143 | 125 | 268 | 148 | 239 | 173 | 253 | 187 |
| *hsa-miR-30c* | 2136 | 1336 | 3714 | 5450 | 4320 | 3798 | 4628 | 6408 | 3939 | 5276 |
| *hsa-miR-30c-1** | 96 | 53 | 142 | 136 | 109 | 66 | 79 | 77 | 71 | 60 |
| *hsa-miR-30c-2** | 393 | 321 | 72 | 310 | 142 | 1180 | 1417 | 1374 | 1331 | 1100 |
| *hsa-miR-30d* | 46441 | 19905 | 22052 | 51544 | 21117 | 10212 | 13686 | 12804 | 12096 | 14059 |
| *hsa-miR-30d** | 11 | 3 | 6 | 16 | 8 | 1 | 5 | 5 | 1 | 2 |
| *hsa-miR-30e* | 4762 | 2530 | 2911 | 2745 | 2375 | 1760 | 2225 | 2628 | 1625 | 2386 |
| *hsa-miR-30e** | 695 | 275 | 625 | 757 | 773 | 463 | 592 | 508 | 358 | 497 |
| *hsa-miR-31* | 37311 | 448 | 1197 | 37688 | 47 | 949 | 315 | 780 | 288 | 722 |
| *hsa-miR-31** | 576 | 7 | 331 | 4196 | 17 | 61 | 24 | 174 | 24 | 93 |
| *hsa-miR-3115* | 4 | 0 | 2 | 0 | 2 | 0 | 0 | 0 | 0 | 0 |
| *hsa-miR-3116* | 6 | 21 | 1 | 2 | 14 | 5 | 2 | 4 | 2 | 5 |
| *hsa-miR-3117* | 11 | 6 | 2 | 13 | 7 | 0 | 4 | 2 | 1 | 4 |
| *hsa-miR-3118* | 0 | 0 | 0 | 0 | 4 | 0 | 0 | 0 | 0 | 0 |
| *hsa-miR-3120* | 0 | 0 | 0 | 0 | 1 | 0 | 2 | 2 | 3 | 2 |
| *hsa-miR-3121* | 7 | 12 | 4 | 17 | 15 | 4 | 4 | 2 | 4 | 5 |
| *hsa-miR-3122* | 2 | 1 | 0 | 2 | 1 | 1 | 0 | 1 | 1 | 0 |
| *hsa-miR-3124* | 22 | 9 | 9 | 30 | 16 | 9 | 13 | 10 | 4 | 14 |
| *hsa-miR-3126-3p* | 1 | 0 | 0 | 0 | 0 | 0 | 1 | 0 | 0 | 1 |
| *hsa-miR-3126-5p* | 5 | 0 | 1 | 3 | 8 | 2 | 1 | 6 | 2 | 4 |
| *hsa-miR-3127* | 11 | 11 | 25 | 61 | 17 | 10 | 9 | 13 | 13 | 24 |
| *hsa-miR-3128* | 4 | 12 | 0 | 10 | 10 | 2 | 5 | 7 | 1 | 3 |
| *hsa-miR-3129* | 8 | 66 | 0 | 7 | 8 | 13 | 9 | 7 | 14 | 8 |
| *hsa-miR-3130-3p* | 6 | 32 | 21 | 8 | 17 | 3 | 9 | 8 | 9 | 13 |
| *hsa-miR-3130-5p* | 1 | 0 | 0 | 2 | 0 | 2 | 1 | 1 | 0 | 0 |
| *hsa-miR-3131* | 6 | 1 | 33 | 89 | 0 | 4 | 0 | 1 | 0 | 2 |
| *hsa-miR-3132* | 0 | 1 | 0 | 0 | 0 | 0 | 0 | 0 | 0 | 2 |
| *hsa-miR-3133* | 1 | 6 | 1 | 1 | 6 | 0 | 1 | 0 | 0 | 1 |
| *hsa-miR-3134* | 1 | 2 | 2 | 2 | 1 | 0 | 0 | 1 | 2 | 0 |
| *hsa-miR-3135* | 2 | 0 | 2 | 1 | 1 | 7 | 6 | 4 | 3 | 5 |
| *hsa-miR-3136* | 5 | 11 | 3 | 1 | 19 | 0 | 3 | 1 | 0 | 0 |
| *hsa-miR-3138* | 8 | 10 | 9 | 31 | 48 | 8 | 1 | 7 | 9 | 7 |
| *hsa-miR-3139* | 6 | 2 | 0 | 0 | 2 | 2 | 0 | 1 | 1 | 0 |
| *hsa-miR-3140* | 2 | 6 | 5 | 8 | 12 | 3 | 6 | 1 | 4 | 1 |
| *hsa-miR-3141* | 10 | 2 | 1 | 26 | 12 | 0 | 1 | 2 | 2 | 2 |
| *hsa-miR-3143* | 2 | 15 | 2 | 14 | 4 | 6 | 2 | 9 | 10 | 5 |
| *hsa-miR-3144-3p* | 0 | 4 | 3 | 4 | 10 | 0 | 2 | 0 | 0 | 0 |
| *hsa-miR-3144-5p* | 0 | 28 | 17 | 23 | 33 | 8 | 1 | 1 | 1 | 3 |
| *hsa-miR-3145* | 6 | 4 | 1 | 7 | 9 | 0 | 0 | 0 | 0 | 0 |
| *hsa-miR-3146* | 3 | 7 | 0 | 0 | 6 | 2 | 1 | 1 | 0 | 2 |
| *hsa-miR-3148* | 0 | 4 | 0 | 0 | 0 | 0 | 0 | 0 | 0 | 0 |
| *hsa-miR-3149* | 7 | 5 | 0 | 0 | 4 | 3 | 0 | 3 | 0 | 1 |
| *hsa-miR-3150* | 1 | 1 | 0 | 0 | 1 | 0 | 0 | 0 | 0 | 0 |
| *hsa-miR-3150b* | 9 | 18 | 1 | 32 | 47 | 3 | 5 | 0 | 2 | 6 |
| *hsa-miR-3151* | 0 | 1 | 0 | 0 | 0 | 2 | 1 | 4 | 0 | 3 |
| *hsa-miR-3152* | 0 | 0 | 0 | 1 | 0 | 0 | 4 | 1 | 3 | 1 |
| *hsa-miR-3154* | 2 | 2 | 0 | 8 | 14 | 16 | 32 | 29 | 29 | 27 |
| *hsa-miR-3155* | 0 | 1 | 1 | 0 | 0 | 0 | 0 | 2 | 0 | 0 |
| *hsa-miR-3156* | 6 | 3 | 1 | 0 | 1 | 0 | 0 | 0 | 0 | 0 |
| *hsa-miR-3157* | 0 | 3 | 2 | 4 | 4 | 0 | 2 | 1 | 1 | 3 |
| *hsa-miR-3158* | 5 | 0 | 4 | 6 | 1 | 1 | 1 | 1 | 1 | 5 |
| *hsa-miR-3159* | 1 | 0 | 1 | 0 | 6 | 0 | 0 | 1 | 1 | 0 |
| *hsa-miR-3160* | 1 | 1 | 3 | 0 | 5 | 0 | 0 | 2 | 2 | 1 |
| *hsa-miR-3161* | 0 | 0 | 0 | 0 | 2 | 0 | 0 | 1 | 0 | 0 |
| *hsa-miR-3162* | 1 | 2 | 0 | 1 | 9 | 0 | 0 | 0 | 0 | 1 |
| *hsa-miR-3163* | 0 | 2 | 0 | 0 | 2 | 5 | 2 | 4 | 3 | 1 |
| *hsa-miR-3164* | 5 | 55 | 134 | 13 | 22 | 14 | 7 | 10 | 12 | 11 |
| *hsa-miR-3165* | 2 | 28 | 0 | 2 | 9 | 1 | 1 | 1 | 0 | 0 |
| *hsa-miR-3166* | 0 | 1 | 0 | 0 | 0 | 1 | 0 | 0 | 0 | 0 |
| *hsa-miR-3167* | 0 | 0 | 0 | 0 | 0 | 0 | 0 | 0 | 0 | 2 |
| *hsa-miR-3169* | 0 | 0 | 0 | 0 | 1 | 0 | 0 | 0 | 0 | 0 |
| *hsa-miR-3170* | 6 | 5 | 4 | 9 | 4 | 2 | 3 | 2 | 0 | 0 |
| *hsa-miR-3171* | 0 | 0 | 0 | 0 | 36 | 1 | 0 | 0 | 0 | 0 |
| *hsa-miR-3173* | 4 | 1 | 0 | 2 | 11 | 0 | 1 | 0 | 0 | 1 |
| *hsa-miR-3174* | 11 | 16 | 9 | 16 | 3 | 1 | 3 | 4 | 1 | 3 |
| *hsa-miR-3175* | 34 | 3 | 3 | 9 | 13 | 8 | 4 | 5 | 3 | 2 |
| *hsa-miR-3176* | 2 | 0 | 0 | 5 | 0 | 0 | 0 | 0 | 0 | 0 |
| *hsa-miR-3177* | 12 | 3 | 2 | 10 | 4 | 0 | 1 | 1 | 4 | 1 |
| *hsa-miR-3179* | 2 | 11 | 0 | 16 | 57 | 7 | 6 | 7 | 8 | 3 |
| *hsa-miR-3180* | 2 | 0 | 0 | 6 | 106 | 0 | 0 | 0 | 0 | 0 |
| *hsa-miR-3180-3p* | 2 | 0 | 0 | 6 | 105 | 0 | 0 | 0 | 0 | 0 |
| *hsa-miR-3180-5p* | 0 | 0 | 1 | 8 | 89 | 0 | 0 | 0 | 0 | 1 |
| *hsa-miR-3183* | 4 | 1 | 1 | 0 | 0 | 1 | 0 | 1 | 0 | 0 |
| *hsa-miR-3186-3p* | 0 | 0 | 0 | 0 | 0 | 0 | 0 | 2 | 1 | 1 |
| *hsa-miR-3186-5p* | 1 | 0 | 0 | 0 | 0 | 0 | 0 | 0 | 0 | 0 |
| *hsa-miR-3187* | 5 | 1 | 4 | 10 | 5 | 1 | 1 | 0 | 0 | 0 |
| *hsa-miR-3188* | 5 | 2 | 1 | 10 | 2 | 4 | 0 | 5 | 7 | 2 |
| *hsa-miR-3189* | 17 | 0 | 4 | 4 | 2 | 1 | 0 | 0 | 0 | 0 |
| *hsa-miR-3190* | 43 | 19 | 19 | 55 | 42 | 22 | 12 | 22 | 24 | 38 |
| *hsa-miR-3191* | 2 | 2 | 2 | 0 | 0 | 2 | 5 | 0 | 0 | 1 |
| *hsa-miR-3192* | 24 | 39 | 15 | 19 | 23 | 29 | 33 | 34 | 35 | 28 |
| *hsa-miR-3193* | 39 | 23 | 19 | 12 | 4 | 3 | 3 | 6 | 1 | 2 |
| *hsa-miR-3198* | 0 | 6 | 3 | 8 | 3 | 0 | 1 | 0 | 0 | 0 |
| *hsa-miR-3199* | 4 | 3 | 1 | 4 | 3 | 9 | 5 | 10 | 3 | 9 |
| *hsa-miR-32* | 203 | 98 | 86 | 86 | 72 | 54 | 28 | 41 | 29 | 33 |
| *hsa-miR-32** | 8 | 4 | 10 | 11 | 11 | 1 | 1 | 3 | 1 | 1 |
| *hsa-miR-3200-3p* | 15 | 12 | 61 | 94 | 44 | 13 | 31 | 16 | 20 | 15 |
| *hsa-miR-3200-5p* | 5 | 12 | 13 | 16 | 25 | 8 | 32 | 19 | 28 | 13 |
| *hsa-miR-3202* | 10 | 6 | 2 | 8 | 14 | 4 | 9 | 5 | 5 | 10 |
| *hsa-miR-320a* | 126343 | 150035 | 198492 | 300609 | 152798 | 284777 | 353197 | 194219 | 331289 | 227923 |
| *hsa-miR-320b* | 1773 | 3008 | 9145 | 15562 | 6405 | 5162 | 5354 | 3901 | 5494 | 4172 |
| *hsa-miR-320c* | 166 | 320 | 2373 | 4490 | 2191 | 290 | 419 | 253 | 301 | 208 |
| *hsa-miR-320d* | 53 | 53 | 384 | 814 | 436 | 74 | 101 | 57 | 68 | 43 |
| *hsa-miR-320e* | 0 | 0 | 1 | 1 | 0 | 0 | 0 | 0 | 0 | 0 |
| *hsa-miR-323-3p* | 59 | 405 | 17 | 94 | 46 | 45 | 48 | 70 | 50 | 58 |
| *hsa-miR-323-5p* | 4 | 21 | 0 | 3 | 0 | 2 | 0 | 0 | 4 | 2 |
| *hsa-miR-323b-3p* | 28 | 45 | 0 | 7 | 7 | 5 | 13 | 10 | 7 | 12 |
| *hsa-miR-324-3p* | 57 | 13 | 70 | 122 | 91 | 19 | 28 | 25 | 17 | 26 |
| *hsa-miR-324-5p* | 652 | 170 | 570 | 747 | 699 | 151 | 209 | 197 | 170 | 262 |
| *hsa-miR-326* | 2 | 0 | 1 | 1 | 4 | 2 | 3 | 1 | 1 | 1 |
| *hsa-miR-328* | 30 | 9 | 19 | 49 | 141 | 109 | 90 | 102 | 111 | 92 |
| *hsa-miR-329* | 17 | 50 | 2 | 10 | 12 | 40 | 36 | 40 | 30 | 32 |
| *hsa-miR-330-3p* | 7441 | 2832 | 3573 | 11944 | 1078 | 1373 | 1530 | 1259 | 1590 | 1610 |
| *hsa-miR-330-5p* | 13 | 2 | 8 | 11 | 3 | 1 | 0 | 1 | 1 | 1 |
| *hsa-miR-331-3p* | 1131 | 63 | 1651 | 432 | 545 | 375 | 154 | 140 | 41 | 810 |
| *hsa-miR-331-5p* | 24 | 10 | 5 | 20 | 20 | 18 | 11 | 9 | 8 | 10 |
| *hsa-miR-335* | 1243 | 547 | 160 | 278 | 2740 | 109 | 219 | 180 | 158 | 98 |
| *hsa-miR-335** | 110 | 92 | 8 | 16 | 244 | 4 | 5 | 0 | 2 | 2 |
| *hsa-miR-337-3p* | 10 | 14 | 4 | 15 | 30 | 53 | 71 | 94 | 49 | 72 |
| *hsa-miR-337-5p* | 2 | 3 | 0 | 4 | 5 | 8 | 4 | 4 | 6 | 10 |
| *hsa-miR-338-3p* | 65 | 60 | 16 | 47 | 68 | 119 | 204 | 256 | 179 | 228 |
| *hsa-miR-338-5p* | 3 | 1 | 1 | 0 | 5 | 15 | 17 | 25 | 14 | 15 |
| *hsa-miR-339-3p* | 219 | 108 | 223 | 278 | 150 | 102 | 127 | 114 | 89 | 144 |
| *hsa-miR-339-5p* | 200 | 120 | 443 | 218 | 191 | 253 | 272 | 163 | 157 | 194 |
| *hsa-miR-33a* | 4919 | 618 | 1092 | 309 | 352 | 701 | 375 | 514 | 189 | 590 |
| *hsa-miR-33a** | 59 | 65 | 41 | 20 | 18 | 6 | 4 | 9 | 6 | 9 |
| *hsa-miR-33b* | 1 | 9 | 10 | 0 | 2 | 2 | 1 | 0 | 0 | 1 |
| *hsa-miR-33b** | 0 | 0 | 6 | 0 | 0 | 0 | 1 | 0 | 0 | 1 |
| *hsa-miR-340* | 4003 | 1163 | 1690 | 1655 | 3434 | 3063 | 2851 | 3865 | 2930 | 3966 |
| *hsa-miR-340** | 0 | 1 | 0 | 0 | 0 | 2 | 1 | 4 | 0 | 2 |
| *hsa-miR-342-3p* | 6333 | 4704 | 3588 | 13282 | 27546 | 5677 | 5934 | 8682 | 6176 | 7493 |
| *hsa-miR-342-5p* | 172 | 80 | 164 | 278 | 446 | 53 | 102 | 122 | 127 | 130 |
| *hsa-miR-345* | 222 | 40 | 82 | 107 | 110 | 39 | 36 | 52 | 25 | 45 |
| *hsa-miR-346* | 4 | 1 | 1 | 1 | 0 | 3 | 7 | 3 | 2 | 0 |
| *hsa-miR-34a* | 1828 | 377 | 907 | 741 | 1141 | 223 | 252 | 379 | 145 | 326 |
| *hsa-miR-34a** | 124 | 54 | 91 | 129 | 158 | 57 | 39 | 75 | 31 | 52 |
| *hsa-miR-34b* | 8 | 35 | 4 | 13 | 38 | 1 | 2 | 3 | 0 | 1 |
| *hsa-miR-34b** | 57 | 83 | 12 | 15 | 37 | 5 | 10 | 11 | 5 | 8 |
| *hsa-miR-34c-3p* | 3 | 11 | 2 | 7 | 34 | 0 | 0 | 0 | 1 | 0 |
| *hsa-miR-34c-5p* | 5084 | 6976 | 678 | 1033 | 1713 | 451 | 561 | 446 | 409 | 441 |
| *hsa-miR-3605-3p* | 1 | 1 | 1 | 0 | 3 | 1 | 0 | 0 | 2 | 1 |
| *hsa-miR-3605-5p* | 109 | 100 | 48 | 143 | 176 | 79 | 96 | 109 | 134 | 142 |
| *hsa-miR-3606* | 0 | 2 | 0 | 0 | 5 | 2 | 4 | 2 | 0 | 1 |
| *hsa-miR-3607-3p* | 4 | 3 | 8 | 8 | 13 | 1 | 0 | 0 | 0 | 0 |
| *hsa-miR-3607-5p* | 3 | 1 | 0 | 0 | 2 | 0 | 0 | 1 | 0 | 1 |
| *hsa-miR-3609* | 6 | 6 | 48 | 20 | 9 | 1 | 0 | 2 | 1 | 1 |
| *hsa-miR-3610* | 9 | 4 | 3 | 4 | 2 | 0 | 0 | 0 | 0 | 2 |
| *hsa-miR-3611* | 1 | 0 | 0 | 0 | 0 | 0 | 0 | 0 | 1 | 0 |
| *hsa-miR-3612* | 0 | 0 | 0 | 0 | 0 | 0 | 1 | 3 | 1 | 1 |
| *hsa-miR-3613-3p* | 73 | 39 | 18 | 192 | 45 | 33 | 33 | 43 | 42 | 46 |
| *hsa-miR-3613-5p* | 25 | 18 | 10 | 12 | 12 | 3 | 9 | 5 | 6 | 10 |
| *hsa-miR-361-3p* | 161 | 226 | 79 | 142 | 172 | 86 | 101 | 168 | 128 | 174 |
| *hsa-miR-3614-3p* | 55 | 68 | 66 | 16 | 97 | 12 | 15 | 28 | 16 | 23 |
| *hsa-miR-3614-5p* | 73 | 43 | 46 | 95 | 141 | 11 | 35 | 22 | 16 | 35 |
| *hsa-miR-3615* | 75 | 6 | 19 | 25 | 14 | 20 | 10 | 16 | 4 | 15 |
| *hsa-miR-361-5p* | 456 | 277 | 435 | 770 | 980 | 267 | 222 | 441 | 245 | 382 |
| *hsa-miR-3616-3p* | 1 | 2 | 0 | 3 | 2 | 0 | 0 | 1 | 0 | 0 |
| *hsa-miR-3616-5p* | 1 | 3 | 0 | 1 | 0 | 0 | 0 | 0 | 0 | 0 |
| *hsa-miR-3617* | 4 | 1 | 0 | 1 | 2 | 5 | 2 | 17 | 1 | 7 |
| *hsa-miR-3618* | 1 | 0 | 0 | 0 | 0 | 0 | 0 | 0 | 0 | 0 |
| *hsa-miR-3620* | 6 | 1 | 2 | 8 | 9 | 0 | 2 | 1 | 0 | 0 |
| *hsa-miR-3622a-3p* | 0 | 0 | 0 | 0 | 0 | 0 | 0 | 1 | 1 | 1 |
| *hsa-miR-3622a-5p* | 0 | 0 | 0 | 0 | 0 | 3 | 7 | 3 | 5 | 4 |
| *hsa-miR-3622b-5p* | 0 | 0 | 0 | 0 | 1 | 0 | 0 | 0 | 0 | 0 |
| *hsa-miR-362-3p* | 65 | 55 | 32 | 29 | 125 | 133 | 109 | 185 | 74 | 125 |
| *hsa-miR-362-5p* | 158 | 63 | 51 | 67 | 88 | 32 | 58 | 72 | 36 | 71 |
| *hsa-miR-363* | 222 | 88 | 50 | 534 | 647 | 347 | 530 | 544 | 533 | 349 |
| *hsa-miR-363** | 3 | 1 | 2 | 7 | 12 | 7 | 17 | 5 | 16 | 13 |
| *hsa-miR-3646* | 2 | 1 | 1 | 10 | 0 | 0 | 0 | 0 | 0 | 0 |
| *hsa-miR-3647-3p* | 17 | 1 | 21 | 7 | 62 | 2 | 0 | 0 | 0 | 2 |
| *hsa-miR-3647-5p* | 1 | 0 | 0 | 0 | 1 | 0 | 0 | 0 | 0 | 0 |
| *hsa-miR-3648* | 9 | 28 | 25 | 21 | 43 | 1 | 0 | 0 | 3 | 4 |
| *hsa-miR-365* | 4013 | 5739 | 1413 | 1366 | 2605 | 6067 | 6559 | 5883 | 5815 | 6582 |
| *hsa-miR-365** | 104 | 382 | 22 | 197 | 79 | 261 | 205 | 144 | 229 | 252 |
| *hsa-miR-3651* | 6 | 3 | 2 | 5 | 6 | 1 | 0 | 1 | 0 | 0 |
| *hsa-miR-3652* | 4 | 0 | 0 | 0 | 0 | 0 | 0 | 0 | 0 | 0 |
| *hsa-miR-3653* | 6 | 3 | 12 | 10 | 10 | 1 | 1 | 0 | 0 | 2 |
| *hsa-miR-3654* | 26 | 14 | 2 | 0 | 0 | 0 | 0 | 1 | 0 | 0 |
| *hsa-miR-3656* | 0 | 0 | 0 | 0 | 0 | 6 | 19 | 8 | 6 | 8 |
| *hsa-miR-3657* | 1 | 1 | 0 | 0 | 2 | 0 | 0 | 0 | 0 | 0 |
| *hsa-miR-3659* | 2 | 0 | 0 | 0 | 1 | 0 | 1 | 0 | 1 | 0 |
| *hsa-miR-3660* | 4 | 119 | 0 | 3 | 126 | 0 | 2 | 2 | 2 | 1 |
| *hsa-miR-3661* | 3 | 2 | 0 | 0 | 0 | 0 | 0 | 1 | 0 | 0 |
| *hsa-miR-3662* | 2 | 3 | 0 | 10 | 1 | 0 | 0 | 0 | 0 | 1 |
| *hsa-miR-3663-3p* | 0 | 0 | 0 | 1 | 0 | 0 | 0 | 0 | 0 | 0 |
| *hsa-miR-3664* | 2 | 0 | 0 | 0 | 1 | 0 | 0 | 0 | 0 | 0 |
| *hsa-miR-3667-3p* | 1 | 0 | 0 | 3 | 0 | 0 | 0 | 0 | 0 | 0 |
| *hsa-miR-3667-5p* | 0 | 1 | 0 | 1 | 4 | 0 | 0 | 0 | 0 | 0 |
| *hsa-miR-3674* | 1 | 5 | 1 | 0 | 1 | 0 | 0 | 0 | 0 | 0 |
| *hsa-miR-3675-3p* | 0 | 0 | 0 | 2 | 0 | 0 | 0 | 0 | 0 | 0 |
| *hsa-miR-3675-5p* | 1 | 1 | 0 | 3 | 0 | 0 | 0 | 0 | 0 | 1 |
| *hsa-miR-3676* | 8 | 0 | 15 | 4 | 6 | 31 | 17 | 6 | 6 | 13 |
| *hsa-miR-3677* | 1 | 0 | 0 | 0 | 0 | 0 | 0 | 0 | 0 | 1 |
| *hsa-miR-3678-3p* | 1 | 0 | 0 | 0 | 4 | 0 | 0 | 0 | 1 | 1 |
| *hsa-miR-3680* | 0 | 0 | 0 | 0 | 2 | 0 | 0 | 1 | 0 | 0 |
| *hsa-miR-3682* | 1 | 0 | 0 | 0 | 6 | 0 | 0 | 2 | 3 | 2 |
| *hsa-miR-3683* | 0 | 0 | 0 | 0 | 0 | 0 | 0 | 0 | 0 | 3 |
| *hsa-miR-3684* | 3 | 5 | 0 | 1 | 3 | 1 | 1 | 4 | 1 | 0 |
| *hsa-miR-3685* | 1 | 1 | 0 | 1 | 0 | 0 | 0 | 1 | 0 | 0 |
| *hsa-miR-3687* | 73 | 198 | 94 | 90 | 80 | 3 | 2 | 6 | 4 | 6 |
| *hsa-miR-3688* | 0 | 0 | 0 | 0 | 1 | 1 | 1 | 1 | 2 | 1 |
| *hsa-miR-3689a-5p* | 0 | 0 | 0 | 0 | 0 | 0 | 0 | 1 | 0 | 0 |
| *hsa-miR-3689b* | 0 | 0 | 0 | 0 | 0 | 0 | 0 | 1 | 0 | 0 |
| *hsa-miR-3690* | 49 | 47 | 0 | 50 | 36 | 24 | 81 | 116 | 70 | 153 |
| *hsa-miR-3691* | 4 | 8 | 1 | 1 | 2 | 3 | 1 | 0 | 3 | 1 |
| *hsa-miR-3692** | 1 | 0 | 1 | 1 | 0 | 0 | 0 | 0 | 0 | 0 |
| *hsa-miR-369-3p* | 37 | 99 | 7 | 43 | 32 | 46 | 57 | 67 | 34 | 38 |
| *hsa-miR-369-5p* | 23 | 24 | 1 | 12 | 7 | 27 | 47 | 52 | 44 | 30 |
| *hsa-miR-370* | 0 | 0 | 0 | 8 | 1 | 3 | 1 | 2 | 2 | 1 |
| *hsa-miR-371-3p* | 3 | 0 | 0 | 81 | 4 | 0 | 0 | 0 | 0 | 0 |
| *hsa-miR-371-5p* | 1176 | 1 | 4 | 6061 | 30 | 6 | 10 | 8 | 7 | 5 |
| *hsa-miR-372* | 276 | 3 | 0 | 1729 | 4 | 1 | 0 | 2 | 2 | 1 |
| *hsa-miR-373* | 3 | 0 | 0 | 61 | 0 | 0 | 0 | 1 | 0 | 0 |
| *hsa-miR-373** | 0 | 0 | 0 | 4 | 0 | 0 | 0 | 0 | 0 | 0 |
| *hsa-miR-374a* | 815 | 331 | 222 | 550 | 910 | 602 | 605 | 871 | 574 | 600 |
| *hsa-miR-374a** | 734 | 466 | 451 | 280 | 832 | 1020 | 880 | 1340 | 817 | 996 |
| *hsa-miR-374b* | 1494 | 1512 | 673 | 3799 | 2449 | 4552 | 4392 | 4503 | 3860 | 3913 |
| *hsa-miR-374b** | 62 | 39 | 25 | 9 | 39 | 57 | 77 | 80 | 73 | 84 |
| *hsa-miR-374c* | 2 | 1 | 1 | 5 | 1 | 5 | 7 | 4 | 5 | 4 |
| *hsa-miR-375* | 78 | 283 | 4 | 8 | 19 | 185 | 54 | 60 | 44 | 51 |
| *hsa-miR-376a* | 10 | 57 | 3 | 9 | 19 | 19 | 11 | 32 | 17 | 18 |
| *hsa-miR-376a** | 2 | 1 | 0 | 0 | 1 | 2 | 4 | 0 | 2 | 1 |
| *hsa-miR-376b* | 28 | 163 | 2 | 11 | 21 | 41 | 35 | 58 | 42 | 36 |
| *hsa-miR-376c* | 122 | 486 | 39 | 112 | 285 | 648 | 527 | 1051 | 487 | 694 |
| *hsa-miR-377* | 4 | 9 | 4 | 12 | 14 | 9 | 11 | 20 | 7 | 8 |
| *hsa-miR-377** | 18 | 16 | 4 | 13 | 8 | 9 | 15 | 15 | 12 | 11 |
| *hsa-miR-378* | 36217 | 17743 | 24320 | 34157 | 14731 | 16350 | 15156 | 20246 | 14276 | 25054 |
| *hsa-miR-378** | 34 | 11 | 31 | 41 | 30 | 25 | 28 | 30 | 22 | 44 |
| *hsa-miR-378b* | 0 | 0 | 1 | 1 | 0 | 0 | 0 | 1 | 0 | 1 |
| *hsa-miR-378c* | 1783 | 215 | 1598 | 1326 | 450 | 1366 | 761 | 593 | 406 | 2084 |
| *hsa-miR-379* | 103 | 169 | 15 | 36 | 56 | 94 | 157 | 174 | 123 | 117 |
| *hsa-miR-379** | 0 | 4 | 0 | 2 | 4 | 4 | 5 | 6 | 3 | 2 |
| *hsa-miR-380* | 2 | 6 | 1 | 3 | 6 | 4 | 3 | 6 | 4 | 6 |
| *hsa-miR-380** | 0 | 3 | 0 | 0 | 0 | 0 | 0 | 0 | 0 | 0 |
| *hsa-miR-381* | 5 | 4 | 3 | 3 | 4 | 15 | 3 | 8 | 2 | 4 |
| *hsa-miR-382* | 260 | 531 | 60 | 149 | 323 | 370 | 573 | 555 | 435 | 399 |
| *hsa-miR-383* | 37 | 12 | 4 | 19 | 15 | 359 | 416 | 415 | 324 | 317 |
| *hsa-miR-3907* | 0 | 1 | 0 | 0 | 1 | 0 | 0 | 0 | 0 | 0 |
| *hsa-miR-3909* | 8 | 4 | 2 | 5 | 12 | 9 | 12 | 2 | 9 | 9 |
| *hsa-miR-3910* | 1 | 7 | 0 | 4 | 3 | 4 | 3 | 10 | 8 | 22 |
| *hsa-miR-3911* | 0 | 0 | 0 | 1 | 1 | 1 | 0 | 0 | 1 | 0 |
| *hsa-miR-3912* | 8 | 8 | 7 | 9 | 10 | 15 | 5 | 14 | 10 | 12 |
| *hsa-miR-3913* | 15 | 3 | 3 | 6 | 6 | 8 | 5 | 3 | 3 | 3 |
| *hsa-miR-3914* | 0 | 0 | 0 | 0 | 0 | 23 | 22 | 25 | 20 | 22 |
| *hsa-miR-3915* | 0 | 0 | 0 | 0 | 0 | 4 | 0 | 0 | 3 | 1 |
| *hsa-miR-3916* | 22 | 27 | 0 | 13 | 1 | 0 | 0 | 2 | 0 | 0 |
| *hsa-miR-3917* | 5 | 1 | 5 | 2 | 4 | 1 | 0 | 1 | 0 | 1 |
| *hsa-miR-3918* | 2 | 1 | 0 | 4 | 2 | 0 | 0 | 1 | 0 | 0 |
| *hsa-miR-3919* | 0 | 0 | 1 | 0 | 0 | 2 | 0 | 0 | 0 | 0 |
| *hsa-miR-3920* | 1 | 0 | 0 | 0 | 0 | 0 | 0 | 0 | 0 | 0 |
| *hsa-miR-3921* | 0 | 2 | 0 | 0 | 0 | 0 | 0 | 0 | 0 | 1 |
| *hsa-miR-3922* | 1 | 2 | 0 | 0 | 1 | 0 | 0 | 0 | 0 | 2 |
| *hsa-miR-3925* | 0 | 2 | 4 | 3 | 0 | 0 | 0 | 0 | 0 | 0 |
| *hsa-miR-3927* | 7 | 4 | 0 | 0 | 0 | 5 | 2 | 0 | 4 | 0 |
| *hsa-miR-3928* | 77 | 42 | 16 | 15 | 15 | 22 | 40 | 19 | 23 | 21 |
| *hsa-miR-3929* | 8 | 4 | 1 | 0 | 1 | 0 | 0 | 0 | 0 | 0 |
| *hsa-miR-3934* | 38 | 97 | 18 | 62 | 32 | 7 | 5 | 11 | 8 | 8 |
| *hsa-miR-3935* | 1 | 3 | 0 | 0 | 1 | 0 | 0 | 0 | 0 | 0 |
| *hsa-miR-3936* | 3 | 10 | 5 | 6 | 4 | 9 | 8 | 2 | 6 | 1 |
| *hsa-miR-3938* | 0 | 0 | 0 | 0 | 1 | 0 | 0 | 1 | 0 | 0 |
| *hsa-miR-3939* | 0 | 1 | 0 | 0 | 0 | 0 | 0 | 1 | 0 | 0 |
| *hsa-miR-3940* | 3 | 13 | 2 | 8 | 3 | 0 | 4 | 2 | 2 | 2 |
| *hsa-miR-3941* | 0 | 3 | 0 | 0 | 0 | 0 | 1 | 0 | 0 | 0 |
| *hsa-miR-3942* | 6 | 7 | 0 | 0 | 10 | 1 | 0 | 0 | 1 | 2 |
| *hsa-miR-3943* | 1 | 4 | 0 | 0 | 2 | 1 | 6 | 2 | 8 | 2 |
| *hsa-miR-3944* | 3 | 0 | 3 | 7 | 12 | 0 | 0 | 0 | 0 | 0 |
| *hsa-miR-3945* | 0 | 0 | 0 | 0 | 0 | 0 | 0 | 1 | 0 | 0 |
| *hsa-miR-409-3p* | 181 | 284 | 23 | 204 | 129 | 84 | 103 | 173 | 110 | 107 |
| *hsa-miR-409-5p* | 43 | 118 | 4 | 36 | 20 | 32 | 46 | 33 | 40 | 35 |
| *hsa-miR-410* | 10 | 43 | 1 | 12 | 19 | 13 | 8 | 22 | 14 | 11 |
| *hsa-miR-411* | 67 | 100 | 12 | 39 | 67 | 127 | 164 | 243 | 199 | 130 |
| *hsa-miR-411** | 3 | 18 | 1 | 12 | 7 | 6 | 15 | 9 | 3 | 10 |
| *hsa-miR-412* | 0 | 0 | 0 | 0 | 0 | 0 | 0 | 1 | 0 | 0 |
| *hsa-miR-421* | 293 | 85 | 86 | 255 | 191 | 89 | 120 | 166 | 117 | 183 |
| *hsa-miR-423-3p* | 2943 | 1523 | 1247 | 2973 | 1801 | 915 | 934 | 605 | 720 | 968 |
| *hsa-miR-423-5p* | 100447 | 133242 | 87372 | 141520 | 80870 | 113922 | 136099 | 64264 | 138902 | 130061 |
| *hsa-miR-424* | 2875 | 1330 | 192 | 1784 | 1382 | 2518 | 1880 | 3857 | 1895 | 3910 |
| *hsa-miR-424** | 731 | 161 | 30 | 754 | 319 | 479 | 531 | 598 | 477 | 806 |
| *hsa-miR-425* | 2509 | 1126 | 2212 | 4899 | 5788 | 499 | 672 | 508 | 666 | 720 |
| *hsa-miR-425** | 998 | 190 | 773 | 862 | 2253 | 260 | 292 | 240 | 189 | 360 |
| *hsa-miR-4286* | 72 | 9 | 103 | 57 | 80 | 23 | 29 | 15 | 9 | 19 |
| *hsa-miR-429* | 4033 | 187 | 3931 | 2070 | 938 | 107 | 31 | 69 | 33 | 45 |
| *hsa-miR-4306* | 0 | 0 | 0 | 0 | 0 | 0 | 1 | 0 | 0 | 0 |
| *hsa-miR-431* | 6 | 2 | 3 | 10 | 3 | 0 | 2 | 0 | 0 | 3 |
| *hsa-miR-431** | 4 | 1 | 1 | 1 | 1 | 1 | 4 | 1 | 5 | 5 |
| *hsa-miR-432* | 531 | 388 | 180 | 633 | 629 | 1008 | 1512 | 1405 | 1531 | 959 |
| *hsa-miR-432** | 0 | 0 | 0 | 0 | 0 | 0 | 2 | 0 | 1 | 0 |
| *hsa-miR-4326* | 8 | 8 | 2 | 19 | 6 | 2 | 1 | 7 | 4 | 4 |
| *hsa-miR-433* | 157 | 60 | 24 | 152 | 79 | 143 | 232 | 176 | 252 | 153 |
| *hsa-miR-448* | 0 | 2 | 37 | 0 | 93 | 2 | 1 | 3 | 2 | 0 |
| *hsa-miR-449a* | 53 | 107 | 20 | 20 | 47 | 6 | 3 | 2 | 5 | 5 |
| *hsa-miR-449b* | 0 | 69 | 7 | 8 | 21 | 0 | 1 | 0 | 1 | 0 |
| *hsa-miR-449b** | 0 | 0 | 0 | 0 | 1 | 0 | 0 | 0 | 1 | 0 |
| *hsa-miR-449c* | 34 | 104 | 55 | 101 | 37 | 3 | 0 | 1 | 2 | 2 |
| *hsa-miR-450a* | 80 | 14 | 10 | 87 | 32 | 45 | 55 | 71 | 49 | 55 |
| *hsa-miR-450b-3p* | 1 | 0 | 0 | 0 | 0 | 0 | 0 | 0 | 0 | 3 |
| *hsa-miR-450b-5p* | 80 | 9 | 2 | 66 | 19 | 24 | 26 | 34 | 22 | 36 |
| *hsa-miR-451* | 12736 | 11121 | 11366 | 45270 | 15374 | 103452 | 205716 | 187964 | 207236 | 128525 |
| *hsa-miR-452* | 3934 | 3838 | 2691 | 1200 | 12480 | 718 | 583 | 803 | 684 | 615 |
| *hsa-miR-452** | 18 | 5 | 15 | 3 | 73 | 4 | 5 | 3 | 2 | 3 |
| *hsa-miR-454* | 14 | 4 | 15 | 29 | 23 | 5 | 11 | 11 | 11 | 8 |
| *hsa-miR-454** | 16 | 8 | 17 | 44 | 31 | 12 | 8 | 17 | 10 | 10 |
| *hsa-miR-455-3p* | 777 | 277 | 76 | 1017 | 322 | 175 | 194 | 326 | 162 | 219 |
| *hsa-miR-455-5p* | 536 | 216 | 21 | 111 | 58 | 68 | 80 | 80 | 68 | 86 |
| *hsa-miR-466* | 2 | 0 | 0 | 5 | 372 | 0 | 0 | 0 | 0 | 0 |
| *hsa-miR-483-3p* | 659 | 20 | 1 | 82 | 6 | 4 | 5 | 10 | 9 | 7 |
| *hsa-miR-483-5p* | 4288 | 236 | 56 | 7080 | 75 | 145 | 160 | 181 | 180 | 147 |
| *hsa-miR-484* | 327 | 252 | 244 | 340 | 558 | 345 | 331 | 440 | 460 | 452 |
| *hsa-miR-485-3p* | 87 | 265 | 3 | 31 | 76 | 102 | 123 | 98 | 95 | 83 |
| *hsa-miR-485-5p* | 241 | 516 | 37 | 150 | 183 | 481 | 882 | 705 | 787 | 645 |
| *hsa-miR-486-3p* | 32 | 183 | 24 | 64 | 12 | 36 | 102 | 64 | 88 | 58 |
| *hsa-miR-486-5p* | 2068 | 5110 | 750 | 9693 | 1559 | 23011 | 40436 | 33733 | 51192 | 21855 |
| *hsa-miR-487a* | 1 | 20 | 2 | 7 | 8 | 4 | 2 | 10 | 6 | 2 |
| *hsa-miR-487b* | 301 | 550 | 48 | 290 | 340 | 362 | 524 | 722 | 491 | 477 |
| *hsa-miR-488** | 2 | 0 | 1 | 1 | 0 | 3 | 3 | 2 | 2 | 3 |
| *hsa-miR-489* | 0 | 3 | 20 | 14 | 95 | 2 | 7 | 12 | 5 | 1 |
| *hsa-miR-490-3p* | 5 | 0 | 0 | 5 | 4 | 95 | 218 | 143 | 186 | 169 |
| *hsa-miR-490-5p* | 1 | 0 | 0 | 1 | 0 | 12 | 4 | 5 | 2 | 42 |
| *hsa-miR-491-3p* | 1 | 0 | 1 | 3 | 0 | 1 | 3 | 4 | 0 | 1 |
| *hsa-miR-491-5p* | 24 | 3 | 10 | 34 | 4 | 8 | 13 | 18 | 17 | 10 |
| *hsa-miR-492* | 3 | 1 | 0 | 3 | 2 | 0 | 0 | 0 | 0 | 0 |
| *hsa-miR-493* | 12 | 12 | 2 | 12 | 14 | 12 | 15 | 10 | 6 | 11 |
| *hsa-miR-493** | 60 | 68 | 18 | 41 | 73 | 70 | 115 | 86 | 132 | 86 |
| *hsa-miR-494* | 78 | 509 | 12 | 98 | 159 | 126 | 172 | 252 | 179 | 175 |
| *hsa-miR-495* | 445 | 2435 | 87 | 765 | 1413 | 763 | 1031 | 1550 | 1099 | 1033 |
| *hsa-miR-496* | 12 | 37 | 6 | 31 | 9 | 6 | 5 | 3 | 4 | 7 |
| *hsa-miR-497* | 700 | 128 | 43 | 300 | 192 | 1193 | 1388 | 1562 | 1255 | 1613 |
| *hsa-miR-497** | 0 | 0 | 0 | 0 | 0 | 1 | 0 | 0 | 0 | 1 |
| *hsa-miR-498* | 99 | 0 | 9 | 431 | 19 | 0 | 0 | 0 | 0 | 0 |
| *hsa-miR-499-3p* | 0 | 0 | 0 | 0 | 0 | 1 | 0 | 0 | 0 | 0 |
| *hsa-miR-499-5p* | 108 | 158 | 146 | 73 | 179 | 454 | 276 | 373 | 265 | 326 |
| *hsa-miR-500a* | 12 | 8 | 7 | 22 | 40 | 11 | 5 | 8 | 2 | 4 |
| *hsa-miR-500a** | 391 | 94 | 517 | 713 | 736 | 204 | 159 | 250 | 116 | 207 |
| *hsa-miR-500b* | 12 | 8 | 7 | 22 | 40 | 11 | 5 | 8 | 2 | 4 |
| *hsa-miR-501-3p* | 128 | 40 | 159 | 182 | 139 | 40 | 30 | 56 | 22 | 31 |
| *hsa-miR-501-5p* | 19 | 7 | 5 | 12 | 25 | 5 | 5 | 5 | 8 | 4 |
| *hsa-miR-502-3p* | 400 | 95 | 542 | 761 | 765 | 218 | 186 | 271 | 140 | 229 |
| *hsa-miR-502-5p* | 3 | 0 | 2 | 6 | 13 | 3 | 7 | 5 | 6 | 6 |
| *hsa-miR-503* | 2547 | 251 | 70 | 1380 | 333 | 338 | 288 | 341 | 285 | 369 |
| *hsa-miR-504* | 39 | 0 | 3 | 6 | 22 | 84 | 59 | 45 | 27 | 115 |
| *hsa-miR-505* | 70 | 55 | 45 | 53 | 275 | 33 | 69 | 74 | 41 | 58 |
| *hsa-miR-505** | 203 | 101 | 46 | 106 | 203 | 118 | 122 | 79 | 120 | 107 |
| *hsa-miR-506* | 0 | 0 | 0 | 0 | 1 | 0 | 0 | 0 | 0 | 0 |
| *hsa-miR-508-3p* | 0 | 7 | 0 | 3 | 56 | 2 | 2 | 1 | 1 | 1 |
| *hsa-miR-508-5p* | 0 | 0 | 0 | 0 | 3 | 0 | 0 | 0 | 0 | 0 |
| *hsa-miR-509-3-5p* | 7 | 10 | 0 | 3 | 53 | 2 | 1 | 1 | 1 | 3 |
| *hsa-miR-509-3p* | 7 | 2 | 2 | 2 | 86 | 0 | 3 | 1 | 1 | 2 |
| *hsa-miR-509-5p* | 1 | 1 | 0 | 3 | 30 | 0 | 1 | 1 | 2 | 0 |
| *hsa-miR-511* | 1 | 4 | 0 | 0 | 1 | 3 | 2 | 0 | 1 | 1 |
| *hsa-miR-512-3p* | 128 | 0 | 9 | 186 | 5 | 0 | 0 | 0 | 0 | 0 |
| *hsa-miR-512-5p* | 144 | 0 | 20 | 764 | 26 | 0 | 0 | 0 | 0 | 0 |
| *hsa-miR-513a-3p* | 0 | 0 | 0 | 0 | 2 | 0 | 0 | 0 | 0 | 0 |
| *hsa-miR-513a-5p* | 3 | 2 | 0 | 4 | 17 | 0 | 1 | 1 | 0 | 0 |
| *hsa-miR-513b* | 0 | 0 | 0 | 1 | 7 | 0 | 0 | 0 | 0 | 0 |
| *hsa-miR-513c* | 1 | 5 | 0 | 20 | 13 | 1 | 0 | 1 | 0 | 0 |
| *hsa-miR-514* | 0 | 0 | 0 | 0 | 6 | 0 | 0 | 0 | 0 | 0 |
| *hsa-miR-514b-5p* | 0 | 0 | 0 | 0 | 5 | 0 | 0 | 0 | 0 | 0 |
| *hsa-miR-515-3p* | 73 | 1 | 3 | 380 | 7 | 0 | 0 | 0 | 0 | 0 |
| *hsa-miR-515-5p* | 285 | 0 | 26 | 1092 | 33 | 1 | 0 | 0 | 0 | 0 |
| *hsa-miR-516a-3p* | 2 | 0 | 0 | 14 | 1 | 0 | 0 | 0 | 0 | 0 |
| *hsa-miR-516a-5p* | 279 | 0 | 1 | 875 | 115 | 0 | 0 | 0 | 0 | 1 |
| *hsa-miR-516b* | 3251 | 11 | 27 | 6489 | 64 | 0 | 3 | 2 | 3 | 1 |
| *hsa-miR-516b** | 2 | 0 | 0 | 14 | 1 | 0 | 0 | 0 | 0 | 0 |
| *hsa-miR-517** | 3 | 0 | 0 | 69 | 0 | 0 | 0 | 0 | 0 | 0 |
| *hsa-miR-517a* | 572 | 2 | 22 | 2548 | 36 | 3 | 1 | 1 | 2 | 2 |
| *hsa-miR-517b* | 572 | 2 | 22 | 2548 | 36 | 3 | 1 | 1 | 2 | 2 |
| *hsa-miR-517c* | 57 | 1 | 0 | 215 | 0 | 0 | 0 | 0 | 0 | 0 |
| *hsa-miR-518a-3p* | 252 | 2 | 5 | 2774 | 10 | 0 | 0 | 0 | 0 | 0 |
| *hsa-miR-518a-5p* | 12 | 0 | 1 | 14 | 1 | 0 | 0 | 0 | 0 | 0 |
| *hsa-miR-518b* | 277 | 0 | 15 | 2380 | 36 | 0 | 0 | 0 | 0 | 0 |
| *hsa-miR-518c* | 72 | 0 | 5 | 676 | 5 | 0 | 0 | 0 | 0 | 1 |
| *hsa-miR-518c** | 67 | 0 | 1 | 169 | 4 | 0 | 0 | 0 | 0 | 0 |
| *hsa-miR-518d-3p* | 1 | 0 | 1 | 9 | 0 | 0 | 0 | 0 | 0 | 0 |
| *hsa-miR-518d-5p* | 16 | 0 | 2 | 61 | 3 | 0 | 0 | 0 | 0 | 0 |
| *hsa-miR-518e* | 123 | 2 | 0 | 1814 | 9 | 0 | 1 | 0 | 0 | 1 |
| *hsa-miR-518e** | 187 | 5 | 7 | 976 | 11 | 0 | 0 | 0 | 0 | 0 |
| *hsa-miR-518f* | 73 | 2 | 3 | 683 | 0 | 0 | 0 | 0 | 0 | 0 |
| *hsa-miR-518f** | 12 | 0 | 2 | 54 | 3 | 0 | 0 | 0 | 0 | 0 |
| *hsa-miR-519a* | 175 | 0 | 4 | 963 | 134 | 0 | 1 | 0 | 0 | 1 |
| *hsa-miR-519a** | 218 | 4 | 5 | 1071 | 45 | 0 | 0 | 0 | 0 | 0 |
| *hsa-miR-519b-3p* | 173 | 0 | 12 | 903 | 10 | 0 | 0 | 1 | 1 | 1 |
| *hsa-miR-519b-5p* | 187 | 5 | 7 | 976 | 11 | 0 | 0 | 0 | 0 | 0 |
| *hsa-miR-519c-3p* | 34 | 0 | 4 | 102 | 2 | 0 | 0 | 0 | 0 | 0 |
| *hsa-miR-519c-5p* | 187 | 5 | 7 | 976 | 11 | 0 | 0 | 0 | 0 | 0 |
| *hsa-miR-519d* | 55 | 0 | 9 | 1075 | 9 | 1 | 0 | 0 | 0 | 1 |
| *hsa-miR-519e* | 0 | 0 | 0 | 17 | 1 | 0 | 0 | 0 | 0 | 0 |
| *hsa-miR-519e** | 10 | 0 | 0 | 24 | 0 | 0 | 0 | 0 | 0 | 0 |
| *hsa-miR-520a-3p* | 241 | 0 | 6 | 803 | 22 | 0 | 0 | 1 | 0 | 0 |
| *hsa-miR-520a-5p* | 52 | 0 | 2 | 131 | 0 | 1 | 0 | 0 | 0 | 0 |
| *hsa-miR-520b* | 12 | 0 | 0 | 225 | 2 | 0 | 0 | 0 | 0 | 0 |
| *hsa-miR-520c-3p* | 37 | 0 | 0 | 338 | 6 | 0 | 0 | 0 | 0 | 0 |
| *hsa-miR-520c-5p* | 16 | 0 | 2 | 60 | 3 | 0 | 0 | 0 | 0 | 0 |
| *hsa-miR-520d-3p* | 43 | 0 | 1 | 126 | 1 | 0 | 0 | 0 | 0 | 0 |
| *hsa-miR-520d-5p* | 20 | 0 | 1 | 23 | 2 | 0 | 0 | 0 | 0 | 1 |
| *hsa-miR-520e* | 1 | 0 | 0 | 20 | 0 | 0 | 0 | 0 | 0 | 0 |
| *hsa-miR-520f* | 38 | 0 | 2 | 219 | 3 | 0 | 0 | 0 | 0 | 0 |
| *hsa-miR-520g* | 63 | 0 | 3 | 513 | 8 | 0 | 0 | 0 | 0 | 0 |
| *hsa-miR-520h* | 32 | 0 | 1 | 288 | 3 | 0 | 0 | 0 | 0 | 0 |
| *hsa-miR-521* | 67 | 0 | 1 | 506 | 61 | 0 | 0 | 1 | 0 | 0 |
| *hsa-miR-522* | 62 | 0 | 0 | 282 | 36 | 0 | 0 | 0 | 0 | 0 |
| *hsa-miR-522** | 187 | 5 | 7 | 975 | 10 | 0 | 0 | 0 | 0 | 0 |
| *hsa-miR-523* | 128 | 0 | 14 | 1680 | 12 | 0 | 1 | 0 | 0 | 0 |
| *hsa-miR-523** | 187 | 5 | 7 | 976 | 11 | 0 | 0 | 0 | 0 | 0 |
| *hsa-miR-524-3p* | 31 | 0 | 1 | 252 | 4 | 0 | 0 | 1 | 0 | 0 |
| *hsa-miR-524-5p* | 71 | 0 | 5 | 240 | 9 | 0 | 0 | 0 | 0 | 0 |
| *hsa-miR-525-3p* | 35 | 0 | 3 | 318 | 3 | 0 | 1 | 0 | 1 | 0 |
| *hsa-miR-525-5p* | 49 | 0 | 1 | 159 | 4 | 0 | 0 | 0 | 0 | 0 |
| *hsa-miR-526a* | 16 | 0 | 2 | 61 | 3 | 0 | 0 | 0 | 0 | 0 |
| *hsa-miR-526b* | 192 | 2 | 13 | 542 | 2 | 1 | 0 | 0 | 0 | 1 |
| *hsa-miR-526b** | 11 | 0 | 0 | 171 | 2 | 0 | 0 | 0 | 0 | 0 |
| *hsa-miR-527* | 10 | 0 | 1 | 13 | 1 | 0 | 0 | 0 | 0 | 0 |
| *hsa-miR-532-3p* | 563 | 304 | 236 | 305 | 360 | 303 | 393 | 465 | 359 | 366 |
| *hsa-miR-532-5p* | 1365 | 416 | 555 | 626 | 534 | 603 | 887 | 944 | 782 | 652 |
| *hsa-miR-539* | 5 | 21 | 0 | 0 | 8 | 6 | 10 | 9 | 3 | 10 |
| *hsa-miR-541** | 1 | 10 | 0 | 1 | 0 | 3 | 2 | 3 | 5 | 5 |
| *hsa-miR-542-3p* | 383 | 43 | 38 | 317 | 59 | 123 | 98 | 144 | 94 | 157 |
| *hsa-miR-542-5p* | 50 | 6 | 5 | 59 | 17 | 20 | 16 | 17 | 17 | 52 |
| *hsa-miR-543* | 189 | 619 | 7 | 85 | 35 | 118 | 179 | 123 | 175 | 111 |
| *hsa-miR-544* | 1 | 3 | 0 | 0 | 0 | 2 | 0 | 1 | 0 | 0 |
| *hsa-miR-544b* | 0 | 0 | 2 | 0 | 14 | 2 | 1 | 3 | 0 | 0 |
| *hsa-miR-545* | 1 | 3 | 1 | 0 | 1 | 2 | 1 | 0 | 0 | 1 |
| *hsa-miR-545** | 0 | 0 | 0 | 0 | 0 | 0 | 0 | 1 | 0 | 1 |
| *hsa-miR-548a-3p* | 11 | 65 | 3 | 11 | 15 | 7 | 9 | 9 | 4 | 4 |
| *hsa-miR-548a-5p* | 0 | 1 | 0 | 0 | 1 | 0 | 0 | 0 | 0 | 0 |
| *hsa-miR-548aa* | 10 | 115 | 2 | 4 | 14 | 6 | 9 | 8 | 10 | 9 |
| *hsa-miR-548b-3p* | 1 | 2 | 1 | 1 | 0 | 3 | 0 | 3 | 3 | 1 |
| *hsa-miR-548b-5p* | 2 | 1 | 2 | 0 | 1 | 0 | 0 | 1 | 0 | 1 |
| *hsa-miR-548c-5p* | 8 | 16 | 1 | 14 | 35 | 7 | 6 | 11 | 3 | 8 |
| *hsa-miR-548d-3p* | 0 | 4 | 0 | 0 | 12 | 2 | 1 | 1 | 0 | 0 |
| *hsa-miR-548d-5p* | 14 | 20 | 18 | 14 | 28 | 11 | 13 | 15 | 14 | 18 |
| *hsa-miR-548e* | 195 | 157 | 52 | 168 | 200 | 54 | 55 | 77 | 61 | 58 |
| *hsa-miR-548f* | 6 | 5 | 1 | 2 | 2 | 0 | 1 | 2 | 2 | 0 |
| *hsa-miR-548h* | 3 | 12 | 2 | 3 | 8 | 8 | 19 | 6 | 19 | 6 |
| *hsa-miR-548i* | 0 | 6 | 0 | 9 | 9 | 14 | 12 | 10 | 9 | 4 |
| *hsa-miR-548j* | 85 | 205 | 33 | 68 | 84 | 22 | 41 | 27 | 48 | 19 |
| *hsa-miR-548k* | 113 | 644 | 23 | 72 | 183 | 42 | 76 | 72 | 59 | 60 |
| *hsa-miR-548l* | 15 | 22 | 21 | 46 | 118 | 24 | 27 | 31 | 30 | 29 |
| *hsa-miR-548n* | 3 | 12 | 3 | 6 | 12 | 4 | 3 | 2 | 3 | 5 |
| *hsa-miR-548o* | 17 | 25 | 17 | 36 | 55 | 10 | 12 | 22 | 15 | 12 |
| *hsa-miR-548p* | 0 | 2 | 1 | 1 | 3 | 3 | 0 | 1 | 2 | 1 |
| *hsa-miR-548q* | 1 | 1 | 0 | 5 | 3 | 0 | 2 | 3 | 2 | 2 |
| *hsa-miR-548s* | 0 | 0 | 0 | 3 | 5 | 0 | 1 | 1 | 0 | 1 |
| *hsa-miR-548t* | 16 | 56 | 12 | 20 | 81 | 11 | 8 | 9 | 11 | 8 |
| *hsa-miR-548u* | 7 | 14 | 5 | 4 | 18 | 8 | 3 | 1 | 4 | 2 |
| *hsa-miR-548v* | 3 | 5 | 0 | 2 | 7 | 0 | 3 | 5 | 2 | 6 |
| *hsa-miR-548w* | 5 | 7 | 3 | 9 | 23 | 8 | 11 | 17 | 8 | 9 |
| *hsa-miR-548x* | 0 | 0 | 0 | 2 | 1986 | 0 | 0 | 0 | 0 | 0 |
| *hsa-miR-548y* | 0 | 0 | 0 | 0 | 8 | 0 | 0 | 0 | 0 | 0 |
| *hsa-miR-548z* | 0 | 1 | 1 | 0 | 3 | 0 | 0 | 2 | 1 | 0 |
| *hsa-miR-549* | 6 | 7 | 6 | 1 | 6 | 0 | 0 | 0 | 0 | 0 |
| *hsa-miR-550a* | 1 | 1 | 2 | 8 | 6 | 2 | 0 | 0 | 0 | 1 |
| *hsa-miR-550a** | 9 | 34 | 6 | 14 | 54 | 29 | 23 | 7 | 19 | 20 |
| *hsa-miR-550b* | 5 | 0 | 0 | 0 | 0 | 0 | 0 | 0 | 0 | 0 |
| *hsa-miR-551a* | 0 | 0 | 1 | 1 | 2 | 1 | 6 | 6 | 4 | 13 |
| *hsa-miR-551b* | 231 | 2 | 47 | 531 | 14 | 199 | 207 | 313 | 147 | 300 |
| *hsa-miR-551b** | 4 | 0 | 2 | 21 | 2 | 4 | 4 | 5 | 3 | 6 |
| *hsa-miR-552* | 4 | 0 | 3 | 0 | 0 | 0 | 1 | 1 | 0 | 0 |
| *hsa-miR-555* | 0 | 0 | 0 | 0 | 2 | 0 | 0 | 0 | 0 | 0 |
| *hsa-miR-556-3p* | 47 | 9 | 20 | 66 | 41 | 6 | 2 | 6 | 2 | 5 |
| *hsa-miR-556-5p* | 0 | 0 | 0 | 1 | 0 | 0 | 0 | 0 | 0 | 0 |
| *hsa-miR-559* | 0 | 0 | 0 | 0 | 1 | 0 | 0 | 0 | 0 | 0 |
| *hsa-miR-570* | 1 | 5 | 0 | 4 | 12 | 0 | 1 | 2 | 2 | 0 |
| *hsa-miR-573* | 2 | 4 | 0 | 1 | 0 | 0 | 0 | 0 | 0 | 0 |
| *hsa-miR-574-3p* | 520 | 828 | 147 | 677 | 1580 | 2095 | 2560 | 2024 | 1938 | 2115 |
| *hsa-miR-574-5p* | 148 | 167 | 70 | 209 | 469 | 75 | 187 | 132 | 192 | 199 |
| *hsa-miR-576-3p* | 18 | 28 | 24 | 32 | 72 | 15 | 21 | 23 | 13 | 18 |
| *hsa-miR-576-5p* | 45 | 130 | 73 | 190 | 714 | 133 | 150 | 180 | 116 | 144 |
| *hsa-miR-577* | 1 | 0 | 1 | 3 | 11 | 0 | 0 | 0 | 1 | 0 |
| *hsa-miR-579* | 1 | 0 | 0 | 1 | 2 | 0 | 0 | 1 | 0 | 0 |
| *hsa-miR-580* | 0 | 0 | 0 | 1 | 2 | 0 | 0 | 1 | 0 | 0 |
| *hsa-miR-581* | 0 | 1 | 0 | 1 | 0 | 0 | 0 | 0 | 1 | 0 |
| *hsa-miR-582-3p* | 77 | 159 | 13 | 31 | 15 | 15 | 29 | 34 | 21 | 35 |
| *hsa-miR-582-5p* | 50 | 181 | 83 | 95 | 36 | 98 | 95 | 162 | 106 | 112 |
| *hsa-miR-584* | 800 | 178 | 393 | 406 | 4778 | 206 | 297 | 218 | 260 | 209 |
| *hsa-miR-585* | 27 | 0 | 14 | 6 | 0 | 14 | 10 | 8 | 4 | 4 |
| *hsa-miR-588* | 0 | 0 | 0 | 0 | 0 | 0 | 0 | 0 | 1 | 0 |
| *hsa-miR-589* | 103 | 118 | 28 | 76 | 57 | 37 | 58 | 52 | 44 | 51 |
| *hsa-miR-589** | 17 | 53 | 16 | 53 | 25 | 34 | 62 | 60 | 38 | 71 |
| *hsa-miR-590-3p* | 27 | 37 | 19 | 28 | 20 | 13 | 15 | 26 | 11 | 21 |
| *hsa-miR-590-5p* | 115 | 101 | 55 | 65 | 73 | 47 | 59 | 68 | 50 | 56 |
| *hsa-miR-592* | 6 | 0 | 1 | 11 | 0 | 1 | 6 | 6 | 6 | 4 |
| *hsa-miR-597* | 0 | 1 | 0 | 0 | 0 | 0 | 0 | 0 | 0 | 0 |
| *hsa-miR-598* | 2127 | 448 | 2593 | 725 | 1889 | 1013 | 533 | 718 | 593 | 1005 |
| *hsa-miR-600* | 1 | 0 | 0 | 0 | 2 | 0 | 0 | 0 | 0 | 0 |
| *hsa-miR-605* | 0 | 0 | 1 | 1 | 0 | 2 | 1 | 5 | 1 | 2 |
| *hsa-miR-609* | 0 | 0 | 2 | 0 | 0 | 0 | 0 | 1 | 0 | 0 |
| *hsa-miR-610* | 0 | 0 | 0 | 0 | 0 | 1 | 0 | 0 | 0 | 0 |
| *hsa-miR-614* | 0 | 0 | 0 | 0 | 0 | 0 | 0 | 1 | 0 | 0 |
| *hsa-miR-615-3p* | 1 | 3 | 0 | 0 | 0 | 0 | 0 | 0 | 0 | 0 |
| *hsa-miR-615-5p* | 2 | 5 | 0 | 3 | 1 | 0 | 0 | 0 | 0 | 1 |
| *hsa-miR-616* | 5 | 1 | 4 | 3 | 7 | 1 | 1 | 3 | 0 | 1 |
| *hsa-miR-616** | 2 | 2 | 1 | 7 | 21 | 6 | 2 | 6 | 3 | 7 |
| *hsa-miR-618* | 5 | 0 | 7 | 9 | 0 | 1 | 5 | 9 | 6 | 4 |
| *hsa-miR-622* | 1 | 0 | 3 | 0 | 1 | 0 | 0 | 0 | 0 | 0 |
| *hsa-miR-624* | 0 | 2 | 0 | 0 | 3 | 0 | 1 | 3 | 1 | 0 |
| *hsa-miR-624** | 8 | 17 | 3 | 4 | 35 | 15 | 18 | 24 | 22 | 7 |
| *hsa-miR-625* | 57 | 31 | 72 | 136 | 69 | 14 | 21 | 23 | 20 | 22 |
| *hsa-miR-625** | 142 | 116 | 122 | 365 | 331 | 95 | 74 | 126 | 109 | 100 |
| *hsa-miR-627* | 2 | 6 | 0 | 0 | 3 | 0 | 1 | 0 | 1 | 1 |
| *hsa-miR-628-3p* | 2 | 2 | 2 | 6 | 7 | 12 | 11 | 14 | 13 | 33 |
| *hsa-miR-628-5p* | 12 | 13 | 3 | 14 | 16 | 23 | 14 | 33 | 24 | 31 |
| *hsa-miR-629* | 1479 | 1713 | 873 | 548 | 0 | 728 | 613 | 849 | 624 | 428 |
| *hsa-miR-629** | 7 | 14 | 4 | 6 | 79 | 5 | 2 | 4 | 3 | 4 |
| *hsa-miR-632* | 0 | 1 | 0 | 0 | 0 | 0 | 0 | 0 | 0 | 0 |
| *hsa-miR-636* | 0 | 1 | 2 | 3 | 8 | 0 | 3 | 1 | 1 | 2 |
| *hsa-miR-639* | 0 | 0 | 0 | 1 | 0 | 0 | 0 | 0 | 0 | 0 |
| *hsa-miR-641* | 13 | 17 | 7 | 21 | 89 | 16 | 21 | 9 | 22 | 26 |
| *hsa-miR-642a* | 3 | 1 | 0 | 4 | 2 | 0 | 0 | 1 | 0 | 3 |
| *hsa-miR-642b* | 0 | 0 | 2 | 3 | 0 | 0 | 0 | 0 | 0 | 0 |
| *hsa-miR-643* | 12 | 5 | 7 | 9 | 7 | 2 | 2 | 2 | 3 | 3 |
| *hsa-miR-651* | 14 | 22 | 15 | 15 | 25 | 6 | 7 | 4 | 3 | 2 |
| *hsa-miR-652* | 217 | 108 | 320 | 634 | 437 | 83 | 89 | 120 | 81 | 101 |
| *hsa-miR-653* | 2 | 2 | 11 | 8 | 11 | 1 | 1 | 3 | 1 | 0 |
| *hsa-miR-654-3p* | 19 | 10 | 8 | 11 | 41 | 43 | 51 | 81 | 34 | 64 |
| *hsa-miR-654-5p* | 12 | 21 | 2 | 10 | 3 | 5 | 13 | 7 | 6 | 7 |
| *hsa-miR-655* | 22 | 81 | 9 | 45 | 62 | 52 | 73 | 88 | 67 | 40 |
| *hsa-miR-656* | 5 | 5 | 0 | 0 | 8 | 12 | 12 | 17 | 6 | 17 |
| *hsa-miR-658* | 2 | 0 | 1 | 0 | 0 | 0 | 0 | 1 | 0 | 0 |
| *hsa-miR-660* | 726 | 289 | 453 | 444 | 1786 | 355 | 437 | 751 | 335 | 419 |
| *hsa-miR-663* | 0 | 6 | 1 | 1 | 1 | 1 | 0 | 0 | 1 | 0 |
| *hsa-miR-663b* | 1 | 5 | 2 | 1 | 6 | 1 | 0 | 0 | 0 | 0 |
| *hsa-miR-664* | 1254 | 821 | 2099 | 1927 | 2679 | 835 | 795 | 1141 | 799 | 920 |
| *hsa-miR-664** | 2868 | 845 | 1130 | 1040 | 1195 | 1341 | 1638 | 1480 | 1527 | 1499 |
| *hsa-miR-665* | 1 | 0 | 0 | 0 | 0 | 3 | 1 | 0 | 0 | 1 |
| *hsa-miR-668* | 2 | 2 | 0 | 2 | 0 | 0 | 1 | 1 | 0 | 2 |
| *hsa-miR-671-3p* | 9 | 3 | 5 | 7 | 5 | 0 | 0 | 3 | 0 | 2 |
| *hsa-miR-671-5p* | 15 | 14 | 10 | 10 | 38 | 0 | 0 | 1 | 1 | 1 |
| *hsa-miR-675* | 581 | 101 | 0 | 14 | 793 | 8 | 14 | 9 | 3 | 14 |
| *hsa-miR-675** | 56 | 14 | 1 | 2 | 43 | 1 | 4 | 1 | 3 | 7 |
| *hsa-miR-676* | 2 | 3 | 11 | 3 | 7 | 8 | 12 | 6 | 14 | 11 |
| *hsa-miR-676** | 1 | 2 | 1 | 0 | 1 | 2 | 2 | 1 | 1 | 6 |
| *hsa-miR-7* | 840 | 1457 | 107 | 329 | 928 | 122 | 174 | 178 | 143 | 163 |
| *hsa-miR-708* | 1042 | 5560 | 21 | 903 | 803 | 105 | 95 | 117 | 89 | 149 |
| *hsa-miR-708** | 89 | 264 | 0 | 19 | 20 | 9 | 7 | 11 | 9 | 8 |
| *hsa-miR-7-1** | 83 | 36 | 34 | 161 | 290 | 81 | 80 | 113 | 93 | 140 |
| *hsa-miR-7-2** | 0 | 2 | 4 | 7 | 2 | 2 | 2 | 6 | 5 | 9 |
| *hsa-miR-720* | 15 | 14 | 30 | 5 | 22 | 6 | 3 | 1 | 2 | 7 |
| *hsa-miR-744* | 6561 | 1629 | 2054 | 5016 | 1471 | 1858 | 1994 | 1421 | 1738 | 2061 |
| *hsa-miR-744** | 9 | 3 | 2 | 5 | 6 | 0 | 3 | 2 | 5 | 5 |
| *hsa-miR-758* | 1 | 1 | 0 | 0 | 1 | 0 | 0 | 0 | 0 | 1 |
| *hsa-miR-760* | 75 | 34 | 110 | 113 | 171 | 46 | 47 | 25 | 40 | 23 |
| *hsa-miR-765* | 32 | 5 | 26 | 68 | 80 | 9 | 23 | 20 | 27 | 13 |
| *hsa-miR-766* | 27 | 27 | 9 | 73 | 87 | 100 | 58 | 107 | 84 | 126 |
| *hsa-miR-767-3p* | 0 | 0 | 0 | 0 | 4 | 0 | 0 | 0 | 0 | 0 |
| *hsa-miR-767-5p* | 11 | 1 | 0 | 437 | 486 | 1 | 0 | 0 | 1 | 0 |
| *hsa-miR-769-3p* | 66 | 29 | 37 | 99 | 70 | 11 | 17 | 5 | 12 | 18 |
| *hsa-miR-769-5p* | 54 | 8 | 31 | 58 | 44 | 11 | 8 | 15 | 12 | 13 |
| *hsa-miR-770-5p* | 0 | 0 | 0 | 0 | 0 | 0 | 0 | 0 | 1 | 1 |
| *hsa-miR-873* | 1 | 0 | 0 | 4 | 6 | 50 | 24 | 20 | 2 | 47 |
| *hsa-miR-874* | 425 | 192 | 86 | 395 | 208 | 96 | 89 | 92 | 97 | 152 |
| *hsa-miR-876-3p* | 0 | 0 | 0 | 0 | 10 | 3 | 1 | 3 | 2 | 1 |
| *hsa-miR-876-5p* | 1 | 0 | 0 | 0 | 12 | 5 | 4 | 5 | 1 | 12 |
| *hsa-miR-877* | 550 | 130 | 415 | 536 | 463 | 75 | 100 | 75 | 132 | 89 |
| *hsa-miR-877** | 3 | 0 | 0 | 1 | 1 | 0 | 0 | 0 | 0 | 1 |
| *hsa-miR-885-3p* | 7 | 0 | 2 | 8 | 1 | 3 | 3 | 4 | 6 | 4 |
| *hsa-miR-885-5p* | 2 | 1 | 5 | 8 | 5 | 6 | 7 | 4 | 4 | 4 |
| *hsa-miR-887* | 46 | 17 | 10 | 87 | 84 | 387 | 226 | 303 | 274 | 501 |
| *hsa-miR-888* | 0 | 0 | 0 | 0 | 1 | 0 | 0 | 0 | 0 | 0 |
| *hsa-miR-889* | 421 | 1079 | 82 | 443 | 293 | 346 | 360 | 429 | 340 | 384 |
| *hsa-miR-890* | 0 | 3 | 0 | 0 | 1 | 0 | 0 | 0 | 0 | 0 |
| *hsa-miR-891a* | 0 | 1 | 0 | 0 | 0 | 1 | 0 | 1 | 1 | 2 |
| *hsa-miR-891b* | 0 | 0 | 0 | 0 | 0 | 1 | 0 | 0 | 0 | 0 |
| *hsa-miR-9* | 28 | 822 | 217 | 89 | 415 | 52 | 116 | 112 | 112 | 110 |
| *hsa-miR-9** | 10 | 303 | 40 | 35 | 150 | 23 | 35 | 25 | 30 | 17 |
| *hsa-miR-92a* | 14085 | 31752 | 11835 | 37280 | 38362 | 10813 | 14168 | 13657 | 14769 | 12064 |
| *hsa-miR-92a-1** | 325 | 914 | 234 | 356 | 397 | 183 | 348 | 193 | 411 | 486 |
| *hsa-miR-92a-2** | 2 | 0 | 0 | 0 | 1 | 1 | 1 | 1 | 0 | 0 |
| *hsa-miR-92b* | 1703 | 2966 | 336 | 1827 | 2497 | 1890 | 1470 | 1601 | 1442 | 2008 |
| *hsa-miR-92b** | 97 | 500 | 52 | 238 | 317 | 352 | 420 | 205 | 482 | 408 |
| *hsa-miR-93* | 5624 | 3880 | 7788 | 17794 | 7115 | 1500 | 2445 | 2729 | 2053 | 2228 |
| *hsa-miR-93** | 19 | 16 | 40 | 78 | 57 | 26 | 31 | 30 | 21 | 31 |
| *hsa-miR-933* | 1 | 0 | 0 | 2 | 2 | 0 | 0 | 0 | 0 | 1 |
| *hsa-miR-934* | 358 | 0 | 148 | 226 | 149 | 5 | 0 | 1 | 0 | 7 |
| *hsa-miR-935* | 5 | 5 | 1 | 5 | 10 | 15 | 28 | 11 | 17 | 8 |
| *hsa-miR-936* | 1 | 16 | 0 | 3 | 0 | 0 | 0 | 0 | 1 | 1 |
| *hsa-miR-937* | 2 | 6 | 0 | 0 | 2 | 0 | 0 | 0 | 1 | 0 |
| *hsa-miR-939* | 2 | 0 | 0 | 0 | 0 | 0 | 0 | 0 | 0 | 1 |
| *hsa-miR-940* | 255 | 117 | 75 | 137 | 91 | 12 | 19 | 12 | 11 | 18 |
| *hsa-miR-941* | 157 | 156 | 56 | 131 | 101 | 19 | 53 | 49 | 38 | 53 |
| *hsa-miR-942* | 15 | 69 | 9 | 30 | 34 | 9 | 18 | 19 | 22 | 17 |
| *hsa-miR-943* | 0 | 0 | 1 | 0 | 0 | 0 | 0 | 0 | 0 | 0 |
| *hsa-miR-944* | 3220 | 3381 | 3107 | 7859 | 7689 | 137 | 40 | 115 | 33 | 68 |
| *hsa-miR-95* | 156 | 60 | 295 | 415 | 215 | 247 | 161 | 283 | 169 | 165 |
| *hsa-miR-96* | 506 | 164 | 1866 | 614 | 215 | 60 | 48 | 43 | 45 | 36 |
| *hsa-miR-96** | 2 | 0 | 8 | 3 | 0 | 0 | 0 | 0 | 0 | 0 |
| *hsa-miR-98* | 8274 | 1256 | 9259 | 5031 | 10090 | 1867 | 1724 | 2116 | 1534 | 2028 |
| *hsa-miR-99a* | 351 | 890 | 5 | 334 | 485 | 6967 | 9226 | 10014 | 8360 | 9766 |
| *hsa-miR-99a** | 10 | 18 | 0 | 4 | 20 | 329 | 418 | 358 | 371 | 323 |
| *hsa-miR-99b* | 33170 | 4379 | 17214 | 69473 | 15507 | 12107 | 13427 | 15453 | 13361 | 17647 |
| *hsa-miR-99b** | 854 | 156 | 368 | 1164 | 361 | 243 | 368 | 231 | 311 | 275 |

| Table S2. The number of total reads and expressed reads of ‘new miRNA candidates’ in deep sequencing | | | | | | | | | | | | | |
| --- | --- | --- | --- | --- | --- | --- | --- | --- | --- | --- | --- | --- | --- |
| Sample |  |  |  | #1 | #2 | #3 | #4 | #5 | #6 | #7 | #8 | #9 | #10 |
| Number of unique ‘ new miRNA candidates ’ |  |  |  | 8 | 7 | 8 | 11 | 10 | 5 | 3 | 4 | 3 | 4 |
|  |  |  |  |  |  |  |  |  |  |  |  |  |  |
| Expressed reads | location | start | end |  |  |  |  |  |  |  |  |  |  |
| TGTTGGCAGGAGCAGAGGATGACT | 1p35.2 | 32258036 | 32258059 | 0 | 0 | 36 | 27 | 0 | 0 | 0 | 0 | 0 | 0 |
| CTGTGGGCTCCTGGGATGGTTC | 1p36.13 | 16847455 | 16847476 | 0 | 40 | 14 | 0 | 0 | 0 | 0 | 0 | 0 | 0 |
| TCTGAGACTAGAGCAAAGCCCTT | 1q42.3 | 236070089 | 236070111 | 19 | 0 | 14 | 0 | 0 | 0 | 0 | 0 | 0 | 0 |
| AAGAACCAAGAATGGGCTGCC | 2q13 | 113054941 | 113058961 | 0 | 76 | 0 | 26 | 0 | 0 | 0 | 0 | 0 | 11 |
| CACAGGCAGGAGACCCCACAG | 3p21.1 | 53797073 | 53797093 | 17 | 0 | 0 | 23 | 0 | 0 | 0 | 0 | 0 | 0 |
| TCGGGGAGATGAGAGACGTGTT | 6p21.1 | 42071665 | 42071686 | 0 | 0 | 37 | 61 | 53 | 8 | 0 | 0 | 0 | 0 |
| AGGGGAACGTGAGGAGAGCTGC | 6p21.33 | 31633509 | 31633530 | 0 | 0 | 0 | 0 | 0 | 0 | 35 | 0 | 84 | 0 |
| CCGGTCAGGGAATGAGGTTTTT | 6p22.1 | 28950036 | 28950057 | 0 | 0 | 49 | 35 | 91 | 42 | 0 | 0 | 0 | 0 |
| TCGGGCGGGAGTGGTGGCTTTT | 6p22.1 | 28918872 | 28918893 | 1290 | 3966 | 756 | 468 | 4224 | 1128 | 2466 | 936 | 2556 | 1488 |
| AAACTGGGCATAGCTGTACTTTT | 8q24.3 | 141538833 | 141538855 | 90 | 59 | 15 | 24 | 0 | 0 | 0 | 0 | 0 | 0 |
| TGGACACTGACCAGGACCCC | 9q34.3 | 139565972 | 139565991 | 33 | 0 | 0 | 29 | 15 | 0 | 0 | 0 | 0 | 0 |
| TGGGAGGAACAAGTATGCATT | 11p15.1 | 16984511 | 16984531 | 268 | 19 | 71 | 154 | 16 | 5 | 0 | 0 | 0 | 0 |
| TACTTACCTGTCCCCTACCCCAC | 12q13.13 | 53292686 | 53292708 | 0 | 0 | 0 | 15 | 17 | 0 | 0 | 0 | 0 | 0 |
| CCCTGCATCGTGGTGGACTGT | 13q32.1 | 97646109 | 97646129 | 0 | 0 | 0 | 0 | 14 | 0 | 0 | 10 | 0 | 0 |
| TTGTGGAAACAATGGTACGGCA | 15q21.1 | 45493421 | 45493442 | 12 | 91 | 0 | 0 | 36 | 31 | 36 | 37 | 38 | 31 |
| GAGTTAGCGGGGAGTGATATATT | 17p13.1 | 8042747 | 8042769 | 0 | 0 | 0 | 0 | 29 | 0 | 0 | 13 | 0 | 12 |
| GCAAAATGATGAGGTACCTGATA | 20p13 | 3194760 | 3194782 | 28 | 51 | 0 | 54 | 22 | 0 | 0 | 0 | 0 | 0 |

| Table S3. Top 21 enriched pathways regulated by *miR-195/497* (P < 1.0E-07) | | | |
| --- | --- | --- | --- |
| **KEGG** | **Annotations** | **Number of annotated genes** | **P-value** |
| **entry** |  |  |  |
| **number** |  |  |  |
| 5200 | Pathways in cancer | 104 | 7.94E-22 |
| 4010 | MAPK signaling pathway | 81 | 5.37E-16 |
| 4144 | Endocytosis | 64 | 2.45E-14 |
| 4910 | Insulin signaling pathway | 48 | 2.37E-12 |
| 4310 | Wnt signaling pathway | 51 | 3.51E-12 |
| 4360 | Axon guidance | 45 | 2.84E-11 |
| 5220 | Chronic myeloid leukemia | 31 | 3.08E-10 |
| 4722 | Neurotrophin signaling pathway | 42 | 4.92E-10 |
| 4810 | Regulation of actin cytoskeleton | 57 | 2.63E-09 |
| 4660 | T cell receptor signaling pathway | 37 | 3.13E-09 |
| 4730 | Long-term depression | 28 | 7.05E-09 |
| 5215 | Prostate cancer | 32 | 9.61E-09 |
| 5211 | Renal cell carcinoma | 28 | 9.63E-09 |
| 4070 | Phosphatidylinositol signaling system | 29 | 1.53E-08 |
| 4724 | Glutamatergic synapse | 39 | 1.62E-08 |
| 5221 | Acute myeloid leukemia | 24 | 3.59E-08 |
| 4912 | GnRH signaling pathway | 33 | 3.89E-08 |
| 4115 | p53 signaling pathway | 26 | 5.70E-08 |
| 4510 | Focal adhesion | 51 | 7.70E-08 |
| 4720 | Long-term potentiation | 26 | 7.81E-08 |
| 4012 | ErbB signaling pathway | 30 | 8.65E-08 |

KEGG = Kyoto Encyclopedia of Genes and Genomes.

Microarray data of bladder cancer were obtained from GEO database.

| Table S4. Upregulated target genes involved in the "Pathways in cancer" | | | | |  |
| --- | --- | --- | --- | --- | --- |
| **Entrez** | **Gene** | **Expression** | **Fold** | **Description** | **P-value** |
| **gene** | **symbol** |  | **change** |  |  |
| **ID** |  |  |  |  |  |
| 332 | *BIRC5* | up | 17.27 | baculoviral IAP repeat containing 5 | 0.00074 |
| 9134 | *CCNE2* | up | 11.01 | cyclin E1 | 0.00074 |
| 7476 | *WNT7A* | up | 9.69 | wingless-type MMTV integration site family, member 7A | 0.00084 |
| 898 | *CCNE1* | up | 6.65 | cyclin E2 | 0.00074 |
| 1871 | *E2F3* | up | 3.91 | E2F transcription factor 3 | 0.00074 |
| 7422 | *VEGFA* | up | 2.88 | vascular endothelial growth factor A | 0.00084 |
| 1282 | *COL4A1* | up | 2.80 | collagen, type IV, alpha 1 | 0.00525 |
| 7849 | *PAX8* | up | 2.66 | paired box 8 | 0.00074 |
| 2246 | *FGF1* | up | 2.63 | fibroblast growth factor 1 (acidic) | 0.00242 |
| 595 | *CCND1* | up | 2.53 | cyclin D1 | 0.01029 |
| 5979 | *RET* | up | 2.18 | ret proto-oncogene | 0.00084 |
| 112398 | *EGLN2* | up | 2.12 | egl nine homolog 2 (C. elegans) | 0.00160 |
| 1855 | *DVL1* | up | 2.11 | dishevelled, dsh homolog 1 (Drosophila) | 0.00084 |
| 10297 | *APC2* | up | 2.05 | adenomatosis polyposis coli 2 | 0.03173 |
| 8817 | *FGF18* | up | 1.95 | fibroblast growth factor 18 | 0.00207 |
| 3728 | *JUP* | up | 1.90 | junction plakoglobin | 0.03101 |
| 5604 | *MAP2K1* | up | 1.85 | mitogen-activated protein kinase kinase 1 | 0.00365 |
| 5915 | *RARB* | up | 1.79 | retinoic acid receptor, beta | 0.00084 |
| 7185 | *TRAF1* | up | 1.71 | TNF receptor-associated factor 1 | 0.00977 |
| 7170 | *TPM3* | up | 1.70 | tropomyosin 3 | 0.00084 |
| 2308 | *FOXO1* | up | 1.69 | forkhead box O1 | 0.00611 |
| 1147 | *CHUK* | up | 1.68 | conserved helix-loop-helix ubiquitous kinase | 0.00247 |
| 5296 | *PIK3R2* | up | 1.50 | phosphoinositide-3-kinase, regulatory subunit 2 (beta) | 0.02672 |
| 861 | *RUNX1* | up | 1.45 | runt-related transcription factor 1 | 0.00824 |
| 2885 | *GRB2* | up | 1.41 | growth factor receptor-bound protein 2provided | 0.00296 |
| 10342 | *TFG* | up | 1.31 | TRK-fused gene | 0.01190 |
| 5894 | *RAF1* | up | 1.28 | v-raf-1 murine leukemia viral oncogene homolog 1 | 0.03247 |

Microarray data of bladder cancer were obtained from GEO database.

Table S5. Insert 3’UTR sequence of *BIRC5* and *WNT7A*

*BIRC5* vector sequence

ggaaaggagatcaacattttcaaattagatgtttcaactgtgctcttgttttgtcttgaaagtggcaccagaggtgcttctgcctgtgcagcgggtgctgctggtaacagtggctgcttctctctctctctctcttttttgggggctcatttttgctgttttgattcccgggcttaccaggtgagaagtgagggagg

*WNT7A* vector sequence

ctgcgggctccctggcaggatgctgagcttgtcttttctgctgaggagggtacttttcctgggtttcctgcaggcatccgtgggggaaaaaaaatctctcagagccctcaactattctgttccacacccaatgctgctccaccctcccccagacacagcccaggtccctccgcggctggagcgaagccttctgcagcagg
